# Direct Readout of Multivalent Chromatin Reader-Nucleosome Interactions by Nucleosome Mass Spectrometry

**DOI:** 10.1021/acscentsci.5c00736

**Published:** 2026-02-05

**Authors:** Alexander S. Lee, Nickolas P. Fisher, Matthew R. Marunde, Pei Su, Laiba F. Khan, Ryan J. Ezell, Zachary B. Gillespie, Bria Graham, Hailey F. Taylor, Ugochi C. Onuoha, Taojunfeng Su, Kevin Jooß, Luis F. Schachner, Harrison A. Fuchs, Kelsey Noll, Matthew J. Meiners, Marcus A. Cheek, Jonathan M. Burg, Zu-Wen Sun, Catherine A. Musselman, Michael-Christopher Keogh, Neil L. Kelleher

**Affiliations:** † Departments of Chemistry and Molecular Biosciences, the Chemistry of Life Processes Institute, and the Proteomics Center of Excellence, 3270 Northwestern University, Evanston, Illinois 60208, United States; ‡ Simpson Querry Center for Epigenetics and Department of Biochemistry and Molecular Genetics, Northwestern University Feinberg School of Medicine, Chicago, Illinois 60611, United States; § EpiCypher, Inc., Research Triangle Park, Durham, North Carolina 27709, United States; ∥ Department of Biochemistry and Molecular Genetics, University of Colorado Anschutz Medical Campus, Aurora, Colorado 80045, United States

## Abstract

Histone post-translational modifications (PTMs) often serve as distinct recognition sites for the recruitment of chromatin-associated proteins (CAPs) for epigenome regulation. While CAP:PTM interactions are extensively studied using histone peptides, this cannot represent the regulatory potential of multisite binding on intact nucleosomes. To overcome this limitation, we applied Nucleosome Mass Spectrometry (Nuc-MS), a native Top-Down MS approach that enables the controlled disassembly and proteoform analysis of CAP:nucleosome (CAP:nuc) complexes. As proof of principle, we show the BPTF plant homeodomain (PHD)-bromodomain (BD) native tandem reader binds synergistically to both PTM classes in fully defined ([H3K4me3K9acK14acK18ac]_2_) nucleosomes. We then extend to explore the engagement of BRD4 (native BD1-BD2), DNMT3A-MPP8 (chimeric PWWP-CD), and *Populus trichocarpa* Short Half Life (PtSHL) (native bromodomain-adjacent homology (BAH-PHD) tandem readers with endogenous HeLa nucleosomes. In the resulting enrichment profiles, BRD4 favors di- and triacetylated histone H4 proteoforms, whereas DNMT3A-MPP8 and PtSHL recover distinct hypermethylated H3 proteoforms. Of note, PtSHL enriches a potential {H3K4me3K27me3} *cis* combinatorial that expands the biology of this bivalent signature previously only described in *trans*. By directly characterizing CAP:nuc complex composition, Nuc-MS informs on the nucleoforms driving binding and thus identifies primary candidates for direct biochemical, structural, and genomic studies.

## Graphical abstract

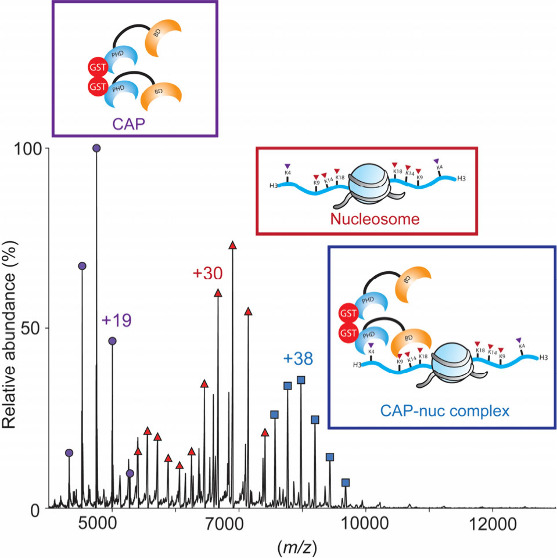

## 1 Introduction

Nucleosomes are the fundamental repeating units of eukaryotic genome organization with arrays forming higher-order chromatin.^1^ Canonical nucleosomes contain two copies of each core histone (H2A, H2B, H3, and H4), forming protein octamers wrapped by 147 bp of DNA.^1^ Within this unit, the globular histone-fold domains mediate extensive structural associations with each other and DNA, while highly charged N-terminal tails dynamically interact with various intra- and inter-nucleosomal surfaces.^1–3^ Introducing diversity to the repeating unit, each histone can be decorated with a myriad of post-translational modifications (PTMs), added and removed by a range of histone-modifying enzymes.^4^ Distinctly modified forms of histones resulting from PTMs and sequence variants are termed “proteoforms”^5^ and their assemblage in distinct nucleosomes, as nucleoforms.^6–8^ To mediate function, site-specific PTMs serve as recognition sites for chromatin-associated proteins (CAPs) through specialized reader domains (or more accurately PTM combinations by grouped domains).^9^ As an example, plant homeodomain (PHD) fingers bind unmodified and methylated lysines,^10–12^ whereas bromodomains (BDs) bind acetylated lysines.^13^ Understanding how CAPs interact with nucleosomes is critical because their regulated engagement at distinct chromatin locations supports the assembly of large nucleoprotein complexes to govern DNA-centric processes including replication, transcription, and repair.^14,15^ Insight thus informs on organismal development^16^ and how dysregulation can impact diseases including cancer^17^ and neurodegeneration.^18^

For decades, most studies that interrogate the binding potential between CAPs and histone PTMs have used histone peptide pulldowns or arrays, predictions from structures of related reader domains with histone peptides, or domain incubations with cell extracts followed by immunoblotting with PTM-specific antibodies.^19–26^ Such approaches are often undermined by reductive formats (minimal reader domains and histone peptides) that poorly represent the regulatory potential of a CAP:nucleosome (CAP:nuc) complex and anti-PTM antibodies of varying quality.^21,24,27^ In these approaches, histone peptides can inform on CAP engagement with combinatorial PTMs on the same histone tail (in *cis*) but not on interactions across nucleosomal histones (in *trans*).^28^ As an additional complexity, while the histone N-terminal tails are traditionally depicted as extending from the nucleosome core, current studies show they instead dynamically associate with nucleosomal DNA with profound regulatory potential.^29–33^ Histone peptides do not supply this nucleosomal DNA directly required by some reader domains, including PWWPs or certain BDs, for effective PTM engagement.^30,34–38^ Lastly, histone peptides cannot provide the diversity of “nonspecific” surfaces that support multivalent CAP:nuc engagement, such as the wrapping nucleosomal DNA and acidic patch.^14,26,39–44^ Semisynthetic PTM-defined nucleosomes provide the ability to interrogate CAP interactions with more representative targets but require direct site-specific chemical synthesis of each histone PTM and their assembly to combinatorial nucleosomes. As such, it is cost-prohibitive to address the immense potential diversity of histone proteoforms and nucleoforms in chromatin.^45–48^ Rather, an approach is needed to screen for the binding of CAPs (alone or within megadalton complexes) to nucleosomes absent *a priori* knowledge, and detect the enriched histone proteoforms and nucleoforms driving engagement for further study.

Mass Spectrometry (MS)-based proteomics has hugely contributed to our understanding of CAP:nuc interactions.^49–56^ However, current MS approaches often require that the nucleosomes and any associated CAPs be denatured and digested to short peptides prior to analysis (*aka*. bottom-up MS).^49–51^ This loses information on CAP complex composition and histone PTM co-occurrence (unless immediately adjacent). Due to this, we still have a limited understanding of how most histone proteoforms contribute to epigenetic regulation, cellular identity, and disease development.^57–59^ Understanding the role of combinatorial PTMs can reveal the crosstalk, where one PTM influences the deposition, recognition, or removal of another, that regulates *in vivo* CAP interactions.^57–62^ Advances in MS, such as Top-Down in which intact proteins are directly analyzed, can provide information on complete histone proteoforms including variant identity and distal PTM combinations (e.g., {H3.2K36me2K79me2}).^6,7,63–70^ Native Top-Down Mass Spectrometry (nTDMS) refers to the controlled disassembly of protein complexes and subsequent characterization of encompassed proteoforms.^71,72^ Nucleosome Mass Spectrometry (Nuc-MS) is a nTDMS approach that can provide information on protein composition and histone proteoforms from semisynthetic or endogenous nucleosomes.^6–8^ Such gains in proteomics capability alongside advances in genomics and protein engineering have driven the need for a new nomenclature to communicate proteoform and nucleoform level data and the degree of experimental certainty.^8^ This new nomenclature is used through the current study (Methods in the SI and Figure 1).

**Figure 1:**
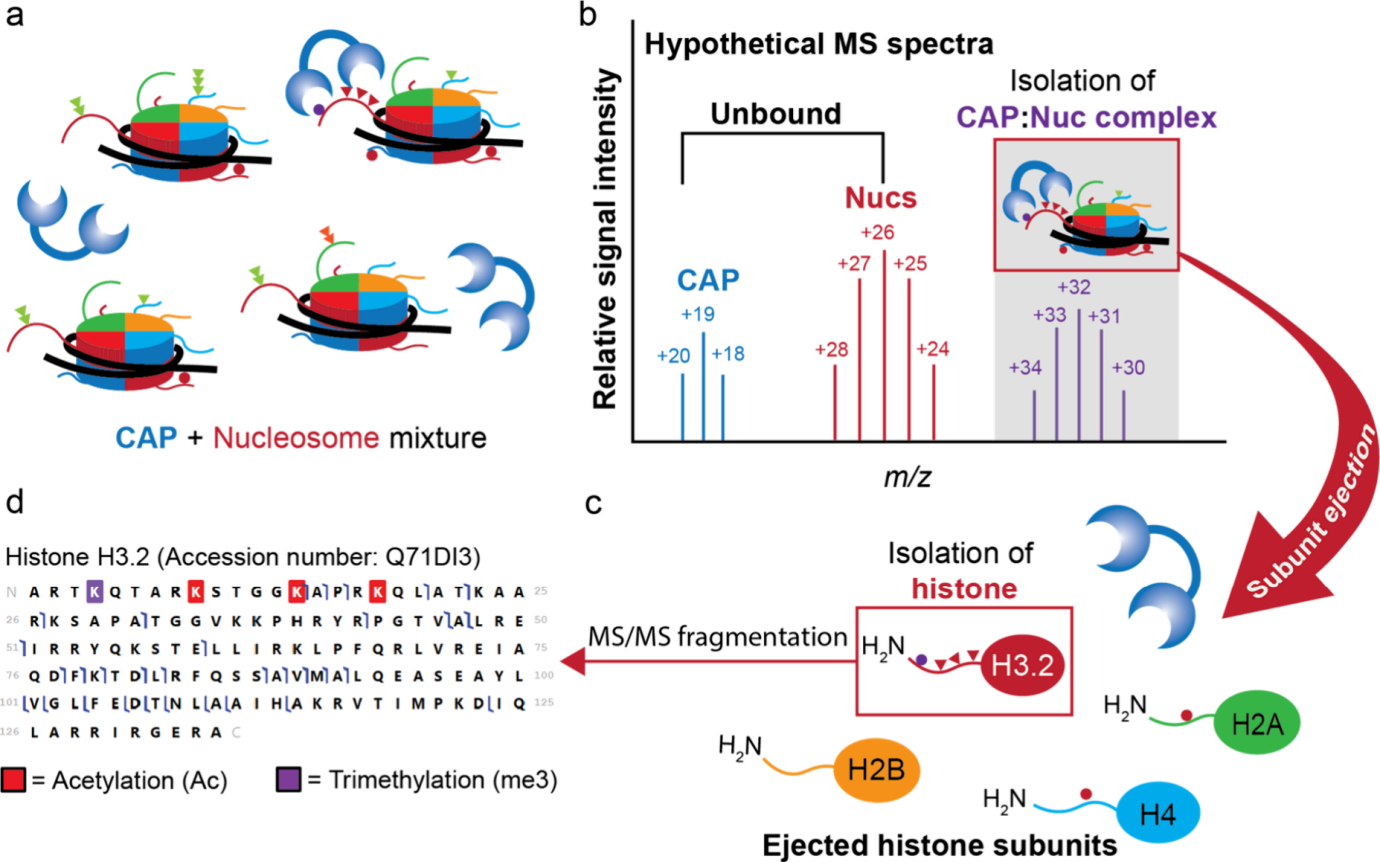
Nucleosome Mass Spectrometry (Nuc-MS) workflow for the direct analysis of CAP-associated nucleoforms. (a) Chromatin-associated proteins (CAPs) are mixed with Nucleosomes ([semisynthetic] or {endogenous} as in ref 8). (b) Native and intact CAP-nucleosome complexes are isolated (MS1). (c) These complexes are activated by collision with nitrogen gas to eject histone subunits (MS2). (d) The desired histone subunit is further isolated and fragmented (pseudo MS3) to determine protein sequence and PTM identity/position.

Here, we broaden the application of Nuc-MS by coupling the approach with CAP-mediated enrichment to (i) confirm the PTMs required for CAP interaction with fully defined nucleoforms, and (ii) identify the histone proteoform landscape after CAP-capture from a pool of endogenous nucleoforms. As proof of principle, we confirm the preference of the BPTF PHD-BD tandem reader for PTM-defined ([H3K4me3K9­acK14acK­18ac]_2_) nucleosomes^32^ and that loss-of-function mutations in either reader domain prevents stable complex formation. We then explored the binding of three distinct tandem reader domain CAPs with endogenous nucleosomes. Here, BRD4 BD1-BD2 enriches nucleosomes with acetylated H2A.Z and histone H4 proteoforms, including with a rare acetylation at H4K44; the DNMT3A-MPP8 chimeric PWWP-CD recovers ({H3K9me­3K36me3}); and the *Populus trichocarpa* Short Half Life (PtSHL) bromodomain-adjacent homology (BAH)-PHD potentially yields ({H3K4me­3K27me3}). The last was of particular note since this “bivalent” PTM combination (active H3K4me3 and repressive H3K27me3) is generally reported to coexist in nucleosomes in *trans* rather than on the same histone tail.^73,74^ These results highlight the potential of Nuc-MS as a tool to reveal the combined elements driving CAP:nuc interactions and suggest focused studies on novel aspects of epigenetic regulation.

## 2 Results and Discussion

The Nuc-MS workflow was developed on commercially available Orbitrap-based mass spectrometers and is composed of three MS elements to interrogate CAP:nuc interactions: (i) intact mass analysis of native CAP:nuc complexes (MS1); (ii) intact mass analysis of ejected histones (MS2); and (iii) tandem MS fragmentation data to assert the enriched histone proteoforms (MS3) (Figure 1). In this study, we used the Orbitrap Q-Exactive Ultra High Mass Range (UHMR) and Orbitrap Tribrid mass spectrometers because of their ability to analyze at high mass ranges (upward of 800 kDa), making them suitable to study intact CAP:nuc complexes (see Methods in the SI). In addition, the Orbitrap Tribrid series is capable of multiple MS/MS fragmentation approaches, supporting the in-depth characterization of histone proteoforms (see Methods in the SI). Of note, the initial experimental steps of CAP-mediated nucleosome enrichment with extensive washing reduce sample complexity and permit a focus on distinguishing the isobaric forms (molecules or ions with the same mass but different compositions: e.g., ac or me3 each add 42 Da) driving engagement. Further, we deliberately focus on tandem readers since combinatorial engagement will invariably be of the greatest affinity and is almost certainly the most biologically meaningful.^28,32,75^

To begin, we conducted a Luminex-based assay to confirm the binding preference of GST-BPTF PHD-BD (hereafter BPTF) for PTM-defined histone H3 peptides and semisynthetic nucleosomes (Figure S1).^76^ As expected, BPTF effectively bound H3_[1–20]_K4me3 and H3_[1–20]_K4me3K9­acK14­acK18ac peptides (with respective EC_50_^Rel^ values of 4.3 and 18.9 nM: Figure S1B and Tables S1–S2). However, in the nucleosome context, BPTF showed a strong preference for the ([H3K4me3K9ac­K14acK18ac]_2_) combinatorial (EC_50_^Rel^ 23.4 nM: Figure S1C and Tables S3–S4).^32,77^

We next used Nuc-MS (MS1) to examine this CAP:nuc interaction and determined that BPTF failed to stably engage ([unmodified]_2_), ([H3K4me3]_2_), or ([H3K4acK9ac­K14acK18ac]_2_) nucleosomes but effectively bound ([H3K4me3K9­acK14acK18ac]_2_) (Figure 2a–d).^32^ As predicted, loss-of-function alanine mutations^23,78^ within the BPTF PHD finger (W2891A, PHD*), bromodomain (N3007A, BD*), or both domains (PHD*BD*) abrogated this nucleosome complexation (Figure S3 and Table S1). By disrupting the BPTF:nuc complex inside the mass spectrometer (MS2), we validated the identity of each histone proteoform at the intact level and their PTMs by MS fragmentation (MS3) (Figure S2). Of note, this complex contained two copies of BPTF, where MS cannot distinguish two independent bindings from one dimerized via the N-terminal GST-tag.^32,79^ However, GST dimerization increases the avidity of GST-BPTF PHD-BD over 6xHis-BPTF PHD-BD for PTM-defined nucleosomes without impacting the specificity profile,^32^ a tag comparison observed with multiple other readers.^75^ This suggests the most probable mode of engagement as GST-dimerized BPTF bound to one nucleosomal H3 tail.

**Figure 2:**
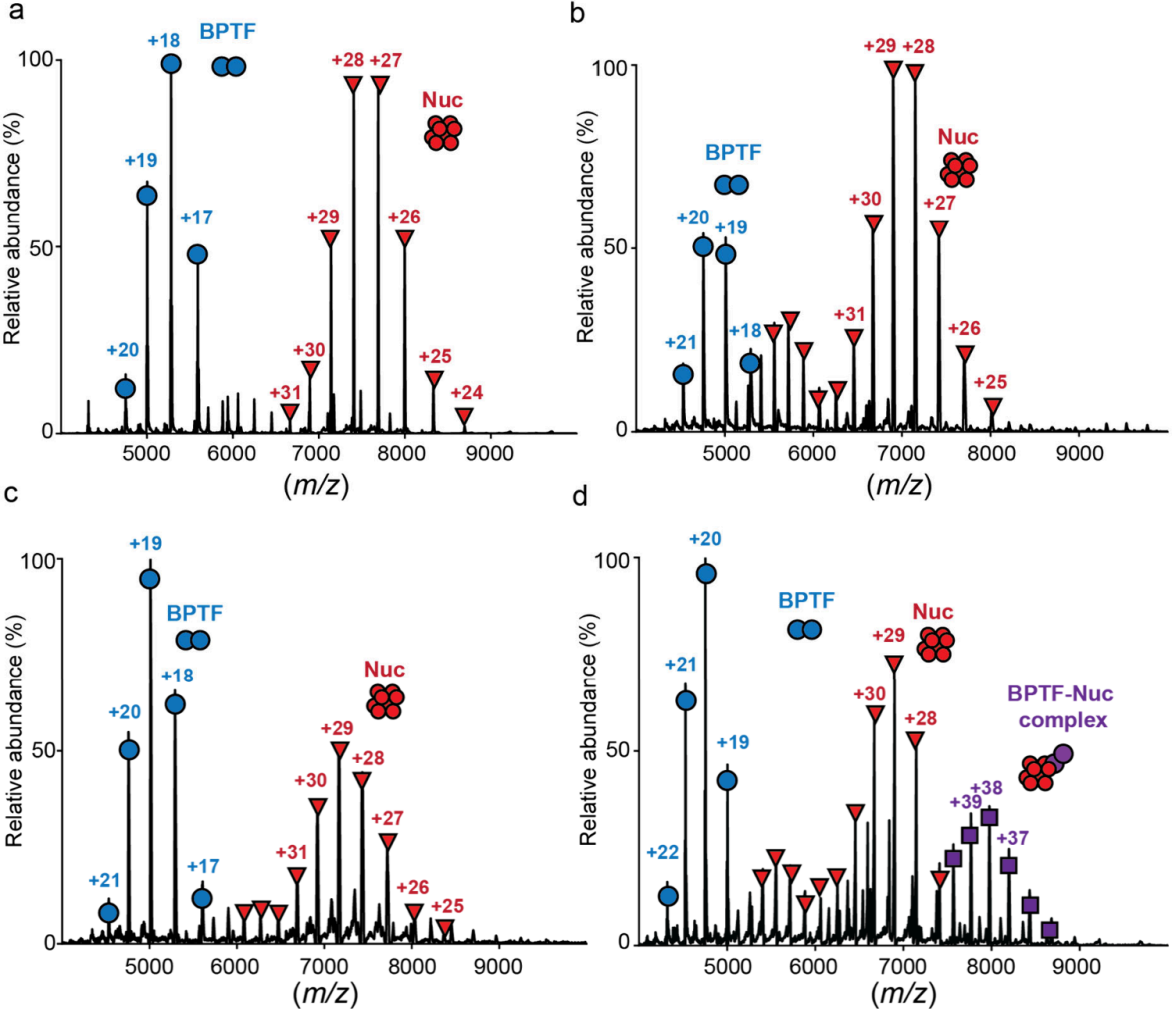
Nuc-MS analysis of BPTF PHD-BD binding to PTM-defined semisynthetic nucleoforms. (a–d) Native MS1 spectrum of intact GST-BPTF PHD-BD with ([unmodified]_2_) (a), ([H3K4me3]_2_) (b), ([H3K4acK9­acK14cK18ac]_2_) (c), or ([H3K4me3K­9acK14cK18ac]_2_) (d) mononucleosomes. The absence of complexation in (a) is reflected by charge state distributions at (+17 to +20) and (+24 to +38), respectively corresponding to a BPTF dimer (possibly via the N-terminal GST-tag^79^) and unmodified nucleosome. Complexation with ([H3K4me3K9­acK14cK18ac]_2_) in (d) is reflected by the appearance of a charge state at (+35 to +41). Representative of three independent experiments shown.

In the above, Nuc-MS can successfully detect multivalent CAP binding to fully defined semisynthetic nucleosomes and delineate the histone proteoforms that support complexation. We next challenged the approach to characterize CAP-enriched endogenous nucleosomes, hypothesizing that different tandem reader domains should recover distinct histone proteoform landscapes. To achieve this, we mixed each bead-immobilized CAP with a pool of endogenous nucleosomes liberated from HeLa chromatin by micrococcal nuclease (Methods in the SI and Figures S4–S5), washed extensively to remove unbound nucleoforms, and then performed Nuc-MS.

Bromodomain-containing protein 4 (BRD4) is a member of the Bromodomain and Extra-Terminal (BET) family and contains tandem BDs (BD1 and BD2) to mediate interactions with nucleosomes hyperacetylated at histones H3 and H4.^13,34,80–85^ BRD4 mediates roles in DNA repair, higher-order chromatin structure, and transcription,^81,84,86–92^ with dysfunction in a range of cancers,^93,94^ and BRD4-targeting therapeutics in active development.^95–102^ The 6xHis-BRD4 BD1-BD2 tandem reader (hereafter BRD4) (Figure S6) was found to enrich hyperacetylated H4 proteoforms with 0.5-, 2.6-, and 4.5-fold increases in the amount of mono-, di-, and triacetylated forms relative to bulk HeLa nucleosomes (Figure 3a). Tandem MS characterization of these isolated H4 proteoforms revealed multiple acetylations at K5, K8, K12, and K16 (reflective of active transcription and euchromatin^13,34,80–85,103^ ) in combination with K20me2 (Figures 3a and S7–S8). We additionally identified a {H4K20me2K44ac} proteoform (Figures 3c and S9) not previously linked to canonical BRD4 function but which may reflect a role in DNA damage and repair.^89,90,104,105^

**Figure 3:**
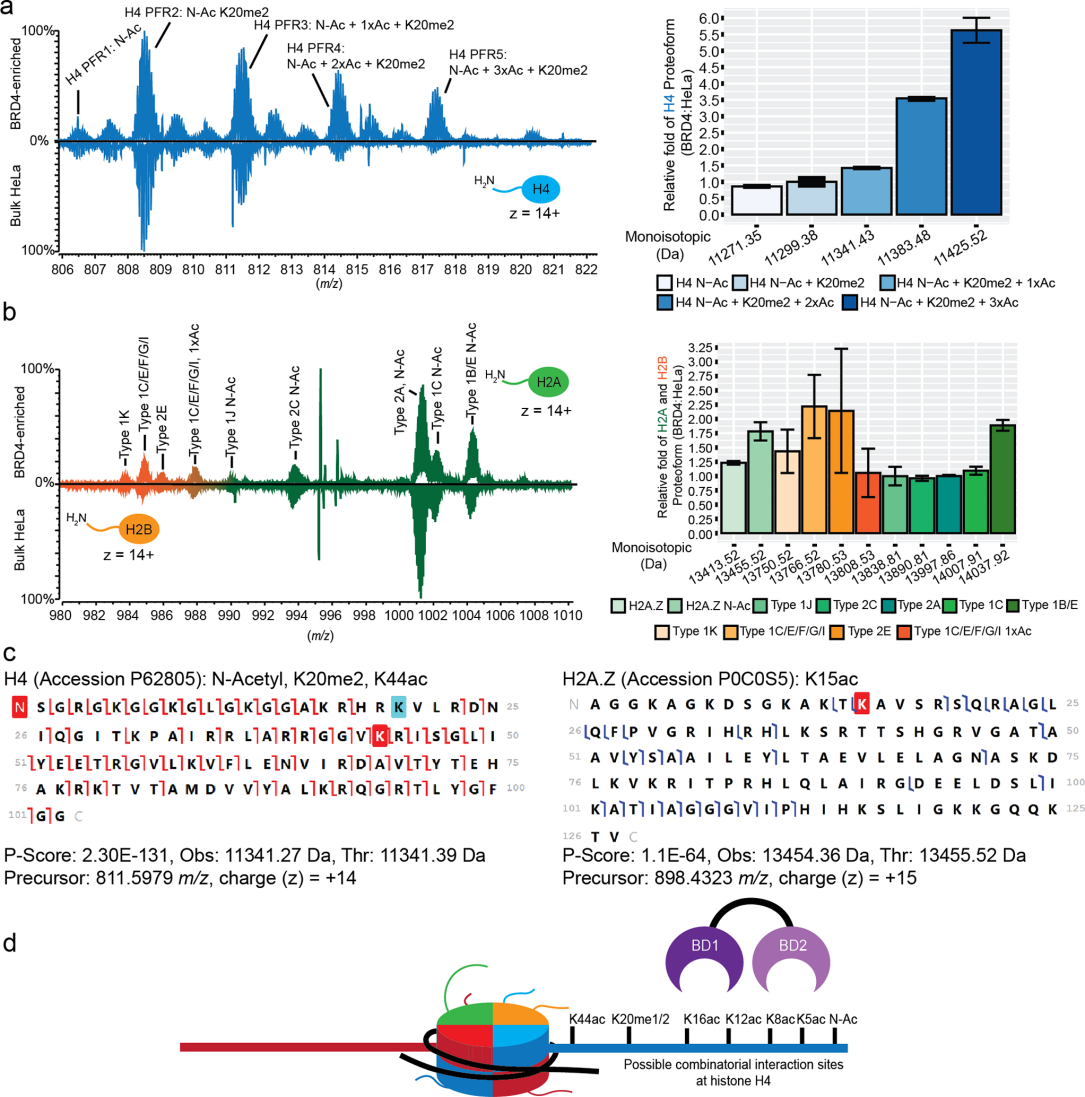
Nuc-MS analysis of BRD4-enriched endogenous nucleoforms. (a, b) Representative MS1 spectra of the histone H4 (a) and H2A/H2B (b) proteoform landscapes (left) and their quantification (right) after nucleosome enrichment by the native tandem reader BRD4 BD1-BD2. In each landscape, acetyl equivalents are represented as (nxAc) with BRD4-enriched nucleosomes above and bulk HeLa nucleosomes below. (c) Graphical fragment maps of {H4K20me2K44ac} (left) and {H2A.ZK15ac} (right). Red forward and reverse flags respectively represent *c* and *z* ions. Blue forward and reverse flags respectively represent *b* and *y* ions. (d) Model of possible BRD4 PTM-binding sites in nucleosome context. Key: Quantification of enrichment in bar graphs (a, b) was calculated by determining the relative abundances of each histone proteoform and normalizing to either H4: N-Acetyl + K20me2 or H2A Type 2A. The relative ratio of BRD4:HeLa bulk is the normalized values of each histone proteoform enriched by BRD4 divided by that in bulk HeLa. Error bars represent standard deviation from the mean.

On further analysis of the BRD4:nuc complex, proteoforms of H3.2 were consistently below the level required for confident tandem MS characterization (not shown), though all that crossed the detection threshold was unchanged relative to bulk HeLa nucleosomes (Figures 3a and S10). Examination of H2A and H2B proteoforms also identified no major differences relative to bulk (Figures 3b and S11–S12) with the exception (∼1.75-fold enriched) an acetylated form of H2A.Z {H2A.ZK15ac}, a variant often associated with gene transcription (Figures 3c and S13).^88,106–108^ Of note, this insight regarding BRD4 preferred nucleoforms, including ({H2A.ZK15ac}·­{H4K5acK12acK16­acK20me2K44ac}) was not previously identified by peptide-centric MS and other biochemical approaches.

We next expanded our study to the histone proteoform landscapes enriched by tandem reader domains that engage lysine methyl states. The DNMT3A-MPP8 reader fuses the H3K36me2/3 binding PWWP domain from *de novo* DNA methyltransferase 3A to the H3K9me3 binding chromodomain of M-phase Phosphoprotein 8 (MPP8) (Figure S14). This chimera is an effective tool for genomic and biochemical studies,^109,110^ and its combinatorial PTM target represents a “bivalent” state (repressive H3K9me3 coincident with transcriptionally active H3K36me3) marking poised transcriptional enhancers.^111^

Nuc-MS spectra of complexes assembled from DNMT3A-MPP8 and endogenous HeLa nucleosomes (Figure S4) identified a proteoform landscape ∼1.5-fold enriched for H3.2 containing four or more methyl equivalents (i.e., multiple additions of CH_3_ (+14) to the primary sequence mass) (Figure 4a). The correct assignment of PTMs within this highly complex MS2 spectrum is challenged by isobaric proteoforms, where a similar mass can represent different structures, such as acetylation [42.0106 Da] vs me3 [42.0471 Da].^63,112^ To address, we conducted denatured LC-Top-Down MS (dLC-TD-MS) targeted on 3×, 6×, and 9× methyl equivalents from this CAP:nuc pool (Figures S15–S16). We also performed a parallel reaction monitoring (PRM) workflow tailored for analysis of the potential proteoforms.^113^ In this manner, we can more confidently assign each proteoform from ever more complex mixtures.

**Figure 4:**
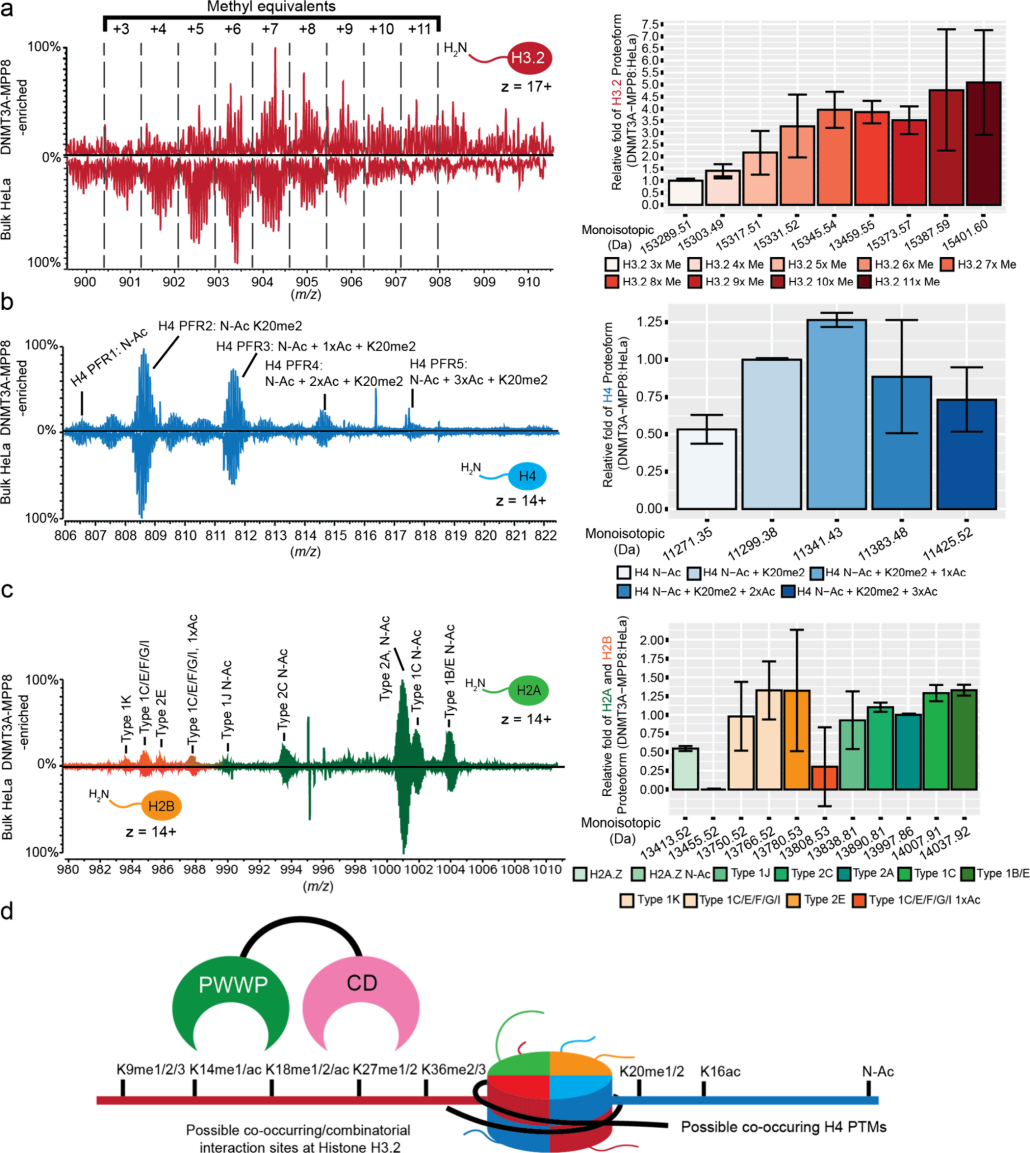
Nuc-MS analysis of DNMT3A-MPP8-enriched endogenous nucleoforms. (a–c) Representative MS1 spectra of the histone H3 (a), H4 (b), and H2A/H2B (c) proteoform landscapes (left) and their quantification (right) after nucleosome enrichment by the chimeric tandem reader DNMT3A-MPP8 PWWP-CD. In each landscape, acetyl and methyl equivalents are, respectively, represented as (nxAc) or (nxMe) with DNMT3A-MPP8 nucleosomes above and bulk HeLa mononucleosomes below. (d) Model of possible DNMT3A-MPP8 PTM-binding sites in nucleosome context. Key: Quantification of enrichment in bar graphs (a–c) was calculated by determining the relative abundances of each histone proteoform and normalizing to either H3.2: 3x Me; H4: N-Acetyl + K20me2; or H2A Type 2A. The relative ratio of DNMT3A-MPP8:HeLa bulk is the normalized values of each histone proteoform enriched by DNMT3A-MPP8 divided by that in bulk HeLa. Error bars represent standard deviation from the mean.

Analysis of the 3× methyl equivalent H3.2 proteoforms revealed a mix of {H3.2K9me1}, {H3.2K9me2}, and {H3.2K9me3}, with {H3.2K9me2} being the most abundant (Figures S15a,b and S16a,d). The 6× methyl equivalent proteoforms contained greater complexity, with differential methylations at K9, K14, K27, and K36, and the two most abundant proteoforms being {H3.2K9me2K36me3} and {H3.2K9me2K27me3} (Figures S15c and S16b,d). Finally, the 9× methyl equivalent H3.2 proteoform pool contained a mix of acetylations and methylations at K9, K14 and K27 (Figures S15d and S16c,d).^73,109,110,114^

Analyses of other histones in DNMT3A-MPP8 enriched nucleosomes revealed {H4K16acK20me2}, a PTM combinatorial associated with active transcription (Figures 4b and S17–S18),^62,65,66^ but no obvious difference in H2A or H2B proteoforms relative to bulk HeLa nucleosomes (Figures 4c and S19–S20). These findings (Figure 4d) are in line with prior orthogonal studies^73,109,110,114^ and validate the sensitivity of Nuc-MS as an approach to usefully interrogate CAP:endogenous nuc complexes.

In the final example, *Populus trichocarpa* Short Half Life (PtSHL) contains a bromodomain-adjacent homology (BAH) domain and PHD finger reader tandem that recognizes H3K4me3 and H3K27me3 with mutually exclusive binding of each PTM state important for SHL-mediated floral repression.^115^ In mammalian cells, H3K4me3 and H3K27me3 are usually nonoverlapping, respectively marking transcriptionally active promoters and polycomb repressed regions. An interesting exception is the canonical “bivalency” signature, where the functionally opposing PTMs coexist within the same nucleosome but on distinct histone H3 tails (a *trans* heterotypic). This holds potential to poise genes for rapid activation or repression during development from embryonic stem cells^55,74,116,117^ and could contribute to plasticity and therapeutic resistance in cancer cells.^118,119^

Nuc-MS of complexes assembled from GST-PtSHL BAH-PHD (hereafter PtSHL) (Figure S21) and endogenous HeLa nucleosomes revealed a H3.2 proteoform profile with increased methyl equivalents, while the H2A, H2B, and H4 profiles were similar to bulk HeLa (Figures 5, S4, and S22–S25). To dissect the H3.2 proteoform profile, we conducted dLC-TD-MS on 3×, 6×, and 9× methyl equivalents (Figures S26 and S27). Here, characterization of the 3× methyl equivalents revealed diverse acetylations and methylations at K4, K9, K14, K18, K23, and K27 (Figures S26a,b and S27a,d). The 6× methyl equivalents contained diverse acetylations and methylations, with the most abundant proteoform (27%) being {H3.2K9me2K27me3} (Figures S26c and S27b,d), but perhaps the most interesting being the *cis* bivalent {H3.2K4me2/­3K27me2/3} (Figures S26c and S27b,d). Lastly, the 9× methyl equivalent proteoforms included {H3.2K9me2K18­acK27me3}, {H3.2K4me­2K14acK18ac},^120,121^ {H3.2K4me2K14­acK27me3} and {H3.2K4me­2K18acK27me3} (Figures S26d and S27c,d). The PtSHL enrichment of {H3.2K4me2/3} and {H3.2K27me2/3} could reflect nucleoforms with each individual PTM^115^ or the bivalent signature in its canonical *trans* format.^55,74,122,123^ However, PtSHL enrichment of {H3.2K4me2/­3K27me2/3} was unexpected (since this PTM *cis* configuration had not (to our knowledge) been reported) but of high confidence since identified in the 6× and 9× methyl equivalent H3.2 proteoforms (Figures S26–S27).

**Figure 5:**
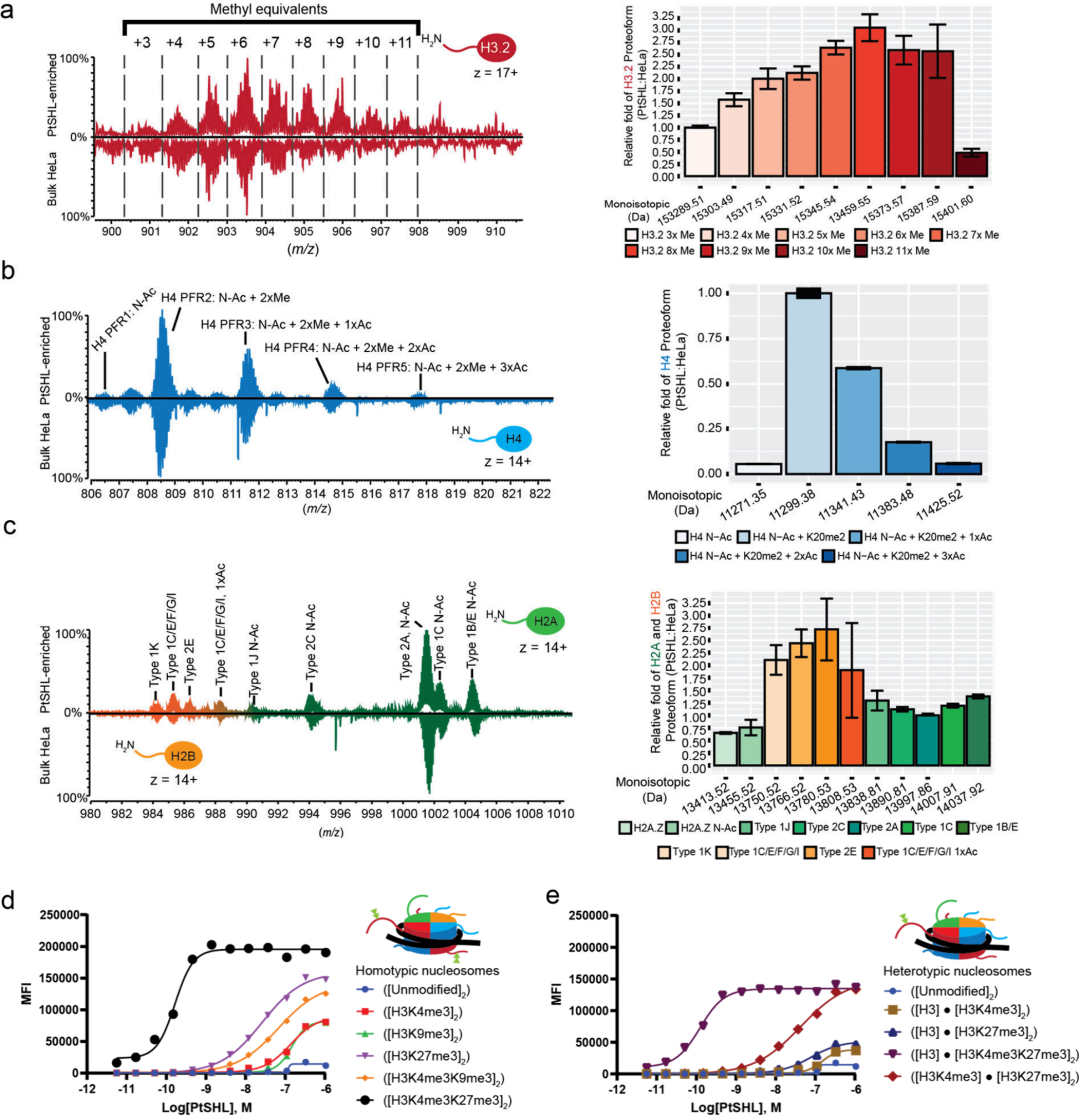
Nuc-MS analysis of PtSHL-enriched endogenous nucleoforms. (a–c) Representative MS1 spectra of the H3.2 (a), H4 (b), and H2A/H2B (c) proteoform landscapes (left) and their quantification (right) after nucleosome enrichment by the native tandem reader PtSHL BAH-PHD. In each landscape, acetyl and methyl equivalents are, respectively, represented as (nxAc) or (nxMe) with PtSHL-enriched above and bulk HeLa nucleosomes below. (d, e) Binding of GST-PtSHL to PTM-defined homotypic (d) or heterotypic (e) nucleosomes by Captify Luminex. Data (logarithmic transformation) is plotted as Median Fluorescence Intensity (MFI) as a function of GST-PtSHL molar concentration (M). Key: Quantification of enrichment in bar graphs (a–c) was calculated by determining the relative abundance of each histone proteoform and normalizing to either H3.2: 3x Me; H4: N-Acetyl + K20me2; or H2A Type 2A. The relative ratio of PtSHL:HeLa bulk is the normalized value of each histone proteoform enriched by PtSHL divided by that in bulk HeLa. Error bars represent standard deviation from the mean.

To further explore the PTM preference of PtSHL, we examined its binding to a multiplex panel of PTM defined nucleosomes by Captify Luminex.^75,76^ When titrated to homotypic targets, the tandem reader showed a profound (>150-fold) preference for ([H3K4me­3K27me3]_2_) over each comprising PTM (Figure 5d and Table S11). This could not distinguish engagement with the *cis* or *trans* configuration (since both are represented), so we examined the titration of PtSHL to heterotypic targets and observed a >350-fold preference for ([H3.2]·[H3.2K4­me3K27me3]) over ([H3.2K4me3]·­[H3.2K27me3]) or its comprising PTMs (Figure 5e and Table S11). This would suggest the ability of PtSHL to enrich even a small amount of its preferred *cis* bivalent target from bulk HeLa chromatin, allowing Nuc-MS to identify the novel PTM configuration. It remains to be determined if {H3.2K4me­3K27me3} represents either an underappreciated biological possibility, or major dysregulation of the chromatin landscape in HeLa cells after >70 years of accumulated genomic aberrations.^124,125^

## 3 Conclusions

Directly informing on the functional relationship between CAPs and the chromatin landscape is a profound challenge for existing methods,^8^ which are often unable to dissect the relative contribution of diverse histone proteoforms to CAP engagement. To address this, we applied Nuc-MS to investigate the proteoform landscape within four tandem-reader CAP:nuc complexes and demonstrated its practicality using commercially available Orbitrap-based mass spectrometers. This included proof of principle with fully defined nucleosomes (BPTF PHD-BD: ([H3K4me3K9­acK14acK18ac])_2_);^32^ a further exploration of reported capability with endogenous nucleosomes for BRD4 BD1-BD2: ({H2A.ZK15ac}·{H4K5acK12­acK16ac K20me2K44ac}) and DNMT3A-­MPP8 PWWP-CD: ({H3.2K9me2/­3K36me2/3}); and a new interrogation of PtSHL BAH-PHD: ({H3.2K4me2/­3K27me2/3}). In each case, we validated known interactions but also revealed novel histone proteoform compositions that would form a basis for follow-up genomic studies. The application of this Nuc-MS workflow to multisubunit CAP complexes and chromatin from healthy and disease states should offer insights to the importance of epigenetic (dys)­regulation.

In closing, while the capabilities of Nuc-MS are appreciated, its current limitations should also be acknowledged. This study used CAP-mediated affinity enrichment rather than resolving denatured histone proteoforms by relative hydrophobicity or electrophoretic mobility.^112,126^ As a result, isobaric histone proteoforms in the CAP:endogenous nuc pool can be challenging to distinguish, though the nonoverlapping charge state (*z*) distributions of native histone proteoforms can somewhat mitigate.^6,71,127,128^ As another limitation, Nuc-MS requires larger sample amounts (see Methods in the SI) than traditional peptide-centric MS-proteomics. This is not a limitation specific to Nuc-MS but rather because measurements of intact proteins and proteoforms have lower sensitively relative to peptides, which easily ionize due to their smaller size.^71,129,130^ Irrespective, Nuc-MS can be performed on the current generation of mass spectrometer instrumentation (Tables S5–S9), and continued technical advances will almost certainly reduce the input requirements.

## Supplementary Material





## Notes

Experimental methods, materials, and additional references pertaining to this study; Figure S1: Differential BPTF PHD-BD binding preference for histone peptides and nucleosomes; Figure S2: Nuc-MS characterization of BPTF bound ([H3K4me3K9acK14cK18ac]_2_) nucleosomes; Figure S3: Both domains in the BPTF PHD-BD tandem binding to semisynthetic ([H3K4me3K9acK14cK18ac]_2_) nucleosomes; Figures S4–S5: Purification and validation of CAP-endogenous nucleosomes; Figure S6: Characterization of 6xHis-BRD4 BD1-BD2 native tandem reader; Figure S7: Characterization of H4 proteoforms from BRD4 enriched endogenous nucleosomes; Figure S8: Tandem MS characterization of H4 proteoforms from BRD4 enriched endogenous nucleosomes; Figure S9: Tandem MS characterization of {H4K20me2K44ac} proteoform from BRD4-enriched endogenous nucleosomes; Figure S10: Relative quantification of H3.2 proteoforms from BRD4-enriched endogenous nucleosomes; Figure S11: Characterization of H2A and H2B proteoforms from BRD4-enriched endogenous nucleosomes; Figure S12: Tandem MS characterization of H2A and H2B proteoforms from BRD4-enriched endogenous nucleosomes; Figure S13: Acetylated histone variant H2A.Z enriched by BRD4; Figure S14: Characterization of GST-DNMT3A-MPP8 PWWP-CD chimeric tandem reader; Figure S15: LC-MS characterization of major H3 proteoforms from DNMT3A-MPP8-enriched endogenous nucleosomes; Figure S16: LC-MS characterization of additional H3 proteoforms from DNMT3A-MPP8-enriched endogenous nucleosomes; Figure S17: Characterization of H4 proteoforms from DNMT3A-MPP8 enriched endogenous nucleosomes by MS/MS fragmentation; Figure S18: Tandem MS characterization of H4 proteoforms from DNMT3A-MPP8-enriched endogenous nucleosomes; Figure S19: Characterization of H2A and H2B proteoforms from DNMT3A-MPP8 enriched endogenous nucleosomes; Figure S20: Tandem MS characterization of H2A and H2B proteoforms from DNMT3A-MPP8 enriched endogenous nucleosomes; Figure S21: Characterization of GST-PtSHL BAH-PHD native tandem reader; Figure S22: Characterization of H4 proteoforms from PtSHL enriched endogenous nucleosomes; Figure S23: Tandem MS characterization of H4 proteoforms from PtSHL enriched endogenous nucleosomes; Figure S24: Characterization of H2A and H2B proteoforms from PtSHL enriched endogenous nucleosomes; Figure S25: Tandem MS characterization of H2A and H2B proteoforms from PtSHL-enriched endogenous; Figure S26: Characterization of major H3 proteoforms from PtSHL-enriched endogenous nucleosomes; Figure S27: Characterization of additional H3 proteoforms from PtSHL-enriched endogenous nucleosomes. Table S1: Relative Effective Concentrations (EC_50_^Rel^) in Luminex assays to measure the interaction between GST-BPTF PHD-BD constructs and PTM-defined histone H3 peptides; Table S2: Averaged (AVG) and Standard Deviation (ST.DEV) of EC_50_^Rel^ Luminex assays to measure interaction between GST-BPTF PHD-BD constructs and PTM-defined histone H3 peptides; Table S3: EC_50_^Rel^ from Luminex assay between GST-tagged BPTF constructs and PTM-defined semisynthetic nucleosomes; Table S4: AVG and ST.DEV of EC_50_^Rel^ from Luminex assays between GST-tagged BPTF constructs and PTM-defined semisynthetic nucleosomes; Table S5: Settings used for intact nMS analysis of BPTF bound nucleosome complexes (MS1); Table S6: Settings used for intact nMS analysis of CAPs (MS1); Table S7: Settings used for MS/MS fragmentation analysis of CAPs (MS2); Table S8: Settings used for characterization of ejected intact histone proteoforms from CAP-nucleosome complexes (MS1); Table S9: Settings used for MS/MS fragmentation analysis of histone proteoforms (MS2); Table S10: Targeted Mass List for Parallel Reaction Monitoring of H3.2 proteoforms for denatured Top-Down LC-MS; Table S11: EC_50_^rel^ for PtSHL binding to defined homotypic and heterotypic nucleosomes (PDF)
Transparent Peer Review report available (DOCX)

## ⊥

Division of BioAnalytical Chemistry, Department of Chemistry and Pharmaceutical Sciences, Vrije Universiteit Amsterdam, Amsterdam 1081 HZ, Netherlands

## ¶

Centre for Analytical Sciences Amsterdam, Amsterdam 1081 HZ, Netherlands.

## ◆

Department of Microchemistry, Proteomics and Lipidomics, Genentech, South San Francisco, California 94080, United States.

## Abbreviations

AmAc: Ammonium acetate
BAH: Bromo adjacent homology
BD: Bromodomain
BRD4: Bromodomain-containing protein 4
BPTF: Bromodomain and PHD finger-containing transcription factor
CD: Chromodomain
CAP: Chromatin-associated protein
dLC-TD-MS: denatured LC-Top-Down MS
GST: Glutathione S-transferase
MNase: Miccrococal nuclease
nMS: native Mass Spectrometry
nTDMS: Native Top-Down Mass Spectrometry
Nuc-MS: Nucleosome Mass Spectrometry
PHD: Plant homeodomain
PtSHL: *Populus trichocarpa* Short Half-life
PTM: post-translational modification
