# Supplementary material for: Direct Readout of Multivalent Chromatin Reader-Nucleosome Interactions by Nucleosome Mass Spectrometry
Source: ACS Cent Sci. 2026 Feb 5;12(2):208–21. doi: 10.1021/acscentsci.5c00736 (PMC12947556; doi:10.1021/acscentsci.5c00736)
Supplement: Supplementary file 1 [file oc5c00736_si_001.docx]

**Supporting Information**

**Direct Readout of Multivalent Chromatin Reader-Nucleosome Interactions by Nucleosome Mass Spectrometry**

Alexander S. Lee^1,2^, Nickolas P. Fisher^1^, Matthew R. Marunde^3^, Pei Su^1^, Laiba F. Khan^3^, Ryan J. Ezell^3^, Zachary B. Gillespie^3^, Bria Graham^3^, Hailey F. Taylor^3^, Ugochi C. Onuoha^3^, Taojunfeng Su^1^, Kevin Jooß^1,4,5^, Luis F. Schachner^1,6^, Harrison A. Fuchs^7^, Kelsey Noll^3^, Matthew J. Meiners^3^, Marcus A. Cheek^3^, Jonathan M. Burg^3^, Zu-Wen Sun^3^, Catherine A. Musselman^7^, Michael-Christopher Keogh^3^ & Neil L. Kelleher^1,2*^

^1^Departments of Chemistry and Molecular Biosciences, the Chemistry of Life Processes Institute, and the Proteomics Center of Excellence, Northwestern University, Evanston, IL 60208, USA

^2^Simpson Querry Center for Epigenetics and Department of Biochemistry and Molecular Genetics, Northwestern University Feinberg School of Medicine, Chicago, IL 60611, USA

^3^EpiCypher, Inc., Research Triangle Park, Durham, NC 27709, USA

^4^Division of BioAnalytical Chemistry, Department of Chemistry and Pharmaceutical Sciences, Vrije Universiteit Amsterdam, Amsterdam, 1081 HZ, NL (Current)

^5^Centre for Analytical Sciences Amsterdam, Amsterdam, 1081 HZ, NL (Current)

^6^Department of Microchemistry, Proteomics and Lipidomics, Genentech, South San Francisco, CA 94080, USA (Current)

^7^Department of Biochemistry and Molecular Genetics, University of Colorado Anschutz Medical Campus, Aurora, CO 80045, USA

**^*^ Correspondence:** Prof. Dr. Neil Kelleher [[n-kelleher@northwestern.edu](mailto:n-kelleher@northwestern.edu)]

**Table of Contents: Total 49 pages**

- Experimental Methods and Materials
- Additional References
- **Figure S1:** Differential BPTF PHD-BD binding preference for histone peptides and nucleosomes.
- **Figure S2:** Nuc-MS characterization of BPTF-bound ([H3K4me3K9acK14cK18ac]_2_) nucleosomes.
- **Figure S3:** Both domains in the BPTF PHD-BD tandem are required for effective binding to semi-synthetic ([H3K4me3K9acK14cK18ac]_2_) nucleosomes.
- **Figure S4:** Extraction and affinity purification of CAPs complexed with endogenous nucleosomes.
- **Figure S5:** Purification of nucleosomal DNA from endogenous HeLa mononucleosomes.
- **Figure S6:** Characterization of 6xHis-BRD4 BD1-BD2 native tandem reader.
- **Figure S7:** Characterization of H4 proteoforms from BRD4-enriched endogenous nucleosomes.
- **Figure S8:** Tandem MS characterization of H4 proteoforms from BRD4-enriched endogenous nucleosomes.
- **Figure S9:** Tandem MS characterization of {H4K20me2K44ac} proteoform from BRD4-enriched endogenous nucleosomes.
- **Figure S10:** Relative quantification of H3.2 proteoforms from BRD4-enriched endogenous nucleosomes.
- **Figure S11:** Characterization of H2A and H2B proteoforms from BRD4-enriched endogenous nucleosomes.
- **Figure S12:** Tandem MS characterization of H2A and H2B proteoforms from BRD4-enriched endogenous nucleosomes.
- **Figure S13:** BRD4 enriches for acetylated histone variant H2A.Z.
- **Figure S14:** Characterization of GST-DNMT3A-MPP8 PWWP-CD chimeric tandem reader.
- **Figure S15:** LC-MS characterization of major H3 proteoforms from DNMT3A-MPP8-enriched endogenous nucleosomes.
- **Figure S16:** LC-MS characterization of additional H3 proteoforms from DNMT3A-MPP8-enriched endogenous nucleosomes.
- **Figure S17:** Characterization of H4 proteoforms from DNMT3A-MPP8-enriched endogenous nucleosomes by MS/MS fragmentation.
- **Figure S18:** Tandem MS characterization of H4 proteoforms from DNMT3A-MPP8-enriched endogenous nucleosomes.
- **Figure S19:** Characterization of H2A and H2B proteoforms from DNMT3A-MPP8-enriched endogenous nucleosomes.
- **Figure S20:** Tandem MS characterization of H2A and H2B proteoforms from DNMT3A-MPP8-enriched endogenous nucleosomes.
- **Figure S21:** Characterization of GST-PtSHL BAH-PHD native tandem reader.
- **Figure S22:** Characterization of H4 proteoforms from PtSHL-enriched endogenous nucleosomes.
- **Figure S23:** Tandem MS characterization of H4 proteoforms from PtSHL-enriched endogenous nucleosomes.
- **Figure S24:** Characterization of H2A and H2B proteoforms from PtSHL-enriched endogenous nucleosomes.
- **Figure S25:** Tandem MS characterization of H2A and H2B proteoforms from PtSHL-enriched endogenous nucleosomes.
- **Figure S26:** LC-MS characterization of major H3 proteoforms from PtSHL-enriched endogenous nucleosomes.
- **Figure S27:** LC-MS characterization of additional H3 proteoforms from PtSHL-enriched endogenous nucleosomes.
- **Table S1:** Relative Effective Concentration (EC_50_^Rel^) in Luminex assays to measure the interaction between GST-BPTF PHD-BD constructs and PTM-defined histone H3 peptides.
- **Table S2:** Averaged (AVG) and Standard Deviation (ST.DEV) of EC_50_^Rel^ Luminex assays to measure the interaction between GST-BPTF PHD-BD constructs and PTM-defined H3 peptides.
- **Table S3:** EC_50_^Rel^ from Luminex assay between GST-tagged BPTF constructs and PTM-defined semi-synthetic nucleosomes.
- **Table S4:** AVG and ST.DEV of EC_50_^Rel^ from Luminex assays between GST-tagged BPTF constructs and PTM-defined semi-synthetic nucleosomes.
- **Table S5:** Settings used for intact nMS analysis of BPTF-bound nucleosome complexes (MS1).
- **Table S6:** Settings used for intact nMS analysis of CAPs (MS1).
- **Table S7:** Settings used for MS/MS fragmentation analysis of CAPs (MS2).
- **Table S8:** Settings used for characterization of ejected intact histone proteoforms from CAP-nucleosome complexes (MS1).
- **Table S9:** Settings used for MS/MS fragmentation analysis of histone proteoforms (MS2).
- **Table S10:** Targeted Mass List for Parallel Reaction Monitoring of H3.2 proteoforms for denatured Top-Down LC-MS.
- **Table S11:** EC_50_^Rel^ for PtSHL binding to defined homotypic and heterotypic nucleosomes.

**Experimental Methods and Materials**

**Protein expression and purification of recombinant chromatin reader constructs.** Human BPTF (Uniprot Q12830) PHD finger-Bromodomain (PHD-BD) cDNA was generated by PCR amplification and cloned into pGEX6p downstream of an N-terminal GST-tag and PreScission Protease cleavage site. Single amino acid substitutions were generated by Q5-site-directed mutagenesis (*NEB*) to produce BPTF mutants PHD* (W2891A), BD* (N3007A) and PHD*BD* (W2891A, N3007A).^1^ Human DNMT3A (Uniprot Q9Y6K1) PWWP domain fused to Human MPP8 (Uniprot Q99549) Chromodomain, and *Populus trichocarpa* SHL (Uniprot Q9FEN9) Bromo-adjacent homology-PHD (BAH-PHD) cDNAs were generated by PCR amplification and cloned into pGEX-6P-2 downstream of an N-terminal GST-tag and PreScission Protease cleavage site. Human BRD4 (Uniprot O60855) BD1-BD2 cDNA was generated by PCR amplification and inserted by Gateway cloning (*Thermo Fisher Scientific*) to pDEST527 (*Addgene* 11518) downstream of an N-terminal 6x-Histidine (6xHis) tag.

Recombinant BPTF, BRD4, DNMT3A-MPP8 and PtSHL constructs were transformed to *E. coli* (T7 Express *lysY*) (*NEB*). Liquid bacterial cultures were grown to OD_600_ ~1, and protein expression induced using 0.8 mM IPTG at 18°C for 16 hrs in LB media. Cells were pelleted by centrifugation and lysed in lysis buffer (25 mM Tris, pH 8.0, 200 mM NaCl, 10% Glycerol, 0.5% CHAPS, 1x EDTA-free Protease Inhibitor Cocktail (PIC), 2.5 mg/mL Lysozyme, 0.5 mM DTT, 1 μL of Universal Nuclease (25 kU; *Pierce*) / mL of cell lysate). Cell lysates were incubated with glutathione sepharose 4B (*Cytiva*), and beads washed three times with lysis buffer. Beads were incubated in glutathione elution buffer (10 mM reduced L-glutathione and 50 mM Tris HCl, pH 8.0) to purify recombinant GST-tagged BPTF, DNMT3A-MPP8 and PtSHL tandem reader proteins. For purification of 6xHis-BRD4 BD1-BD2, cell lysates were mixed with Ni-NTA agarose (*Qiagen*) and beads washed three times using lysis buffer. Beads were incubated with lysis buffer supplemented with 300 mM Imidazole to purify 6xHis-BRD4. Purified chromatin-associated proteins were buffer exchanged to storage buffer (20 mM Tris pH 8.0, 200 mM NaCl, 20% Glycerol, 1 mM DTT) prior to aliquoting and freezing at -80^o^C.

**Semi-synthetic nucleosomes.** Fully defined semi-synthetic mononucleosomes (*EpiCypher*) were produced as previously.^2,3^ This study uses a nucleosome nomenclature recently devised for accurate scientific communication in the chromatin and epigenetic fields.^4^ Here ([H3K4me3]_2_) indicates a fully defined semi-synthetic homotypic nucleosome where other positions not denoted are understood to be definitively unmodified major histones. For native material, ({H3K4me3}) indicates a partially understood nucleoform where the named PTM is experimentally known to exist (as by immunoprecipitating H3K4me3 from a cell extract), but other sites of potential modification not denoted can be understood of undefined status.

**Cell Culture.** HeLa S3 cells (at confluence ~5-8x10^5^ cells/mL) were cultured in DMEM supplemented with 1% Penicillin / Streptomycin and 10% HyClone Bovine Calf Serum (*Cytiva*) in spinner flasks in 5% CO2 incubator at 37°C.

**Extraction and purification of endogenous mononucleosomes.** HeLa S3 cells (~ 1x10^8^ per pellet) were collected and lysed under hypotonic condition with 2x pelleted cell volume (PCV) buffer A (10 mM HEPES, pH 7.9, 10 mM KCl, 1.5 mM MgCl_2_, 340 mM sucrose, 10% Glycerol, 10 mM β-glycerophosphate, 5 mM sodium butyrate, 0.5 mM DTT, and 1x EDTA-free PIC). Buffer A supplemented with 0.2% Triton X-100 was added in volume equal to cell suspension, and incubated on ice for 15 mins. Cells were centrifuged at 1,300 x g for five mins to release nuclei. Pelleted nuclei were washed with 2x PCV of buffer A and centrifuged at 1,300 x g for 15 mins. Pelleted nuclei were resuspended in 2x PCV of buffer A. Nuclei suspension was supplemented with 2 mM CaCl and micrococcal nuclease (*NEB* M0247S) added (5 μL MNase / mL nuclei suspension). Suspension was incubated on a thermal mixer (*Eppendorf*) at 37°C for 30 mins at 600 RPM. Digestion was quenched by addition of 2 mM EDTA and 1 mM EGTA and incubated on ice for 10 minutes. Nuclei suspension was supplemented with 0.05% Triton X-100 followed by 100 mM KCl and 300 mM NaCl in a dropwise fashion and incubated on ice for 30 mins. Finally, nuclei suspension was clarified by centrifugation at 20,000 x g for 20 minutes at 4°C. The resulting supernatant containing soluble mononucleosomes (~ yield 1 mg / mL of nucleosome extract) were used for immunoprecipitation. DNA was purified from each mononucleosome extract (~10 μg) by Phenol-Chloroform extraction. Nucleosomal DNA (~150 bp) was resolved on a D1000 ScreenTape (*Agilent*) and analyzed on a TapeStation 4200 (*Agilent*).

**Purification of chromatin reader-bound endogenous nucleosome complexes.** Glutathione Sepharose 4B beads (*Cytiva*) (150 μL bead slurry) were saturated with excess GST-PtSHL (100 μg) in PtSHL binding buffer (20 mM Tris pH 7.5, 150 mM NaCl, 0.01% Tween-20, 0.01% BSA), or GST-DNMT3A-MPP8 (100 μg) in DNMT3A-MPP8 binding buffer (20 mM Tris pH 7.5, 150 mM NaCl, 0.01% NP-40, 0.01% BSA + 1 mM DTT). Ni-NTA agarose beads (*Qiagen*) (150 μL bead slurry) were saturated with excess 6xHis-BRD4 (100 μg) in BRD4 binding buffer (50 mM Tris-HCl pH 8.0, 100 mM NaCl, 0.1% NP-40). Beads were incubated at 4°C for two hrs with rotation. Beads were washed four times with 500 μL of respective binding buffer. CAP-bound beads were incubated with 200 μg of endogenous mononucleosomes in 200 μL of respective binding buffer and incubated at either 4°C overnight or 25°C for two hrs. Controls containing no CAPs were mixed with endogenous mononucleosomes to determine nonspecific binding to beads. Beads were washed three times with 500 μL of respective binding buffer to remove nonspecific interactions. CAP-endogenous nucleosome complexes were eluted under native conditions with either 150 μL of GST elution buffer (125 mM Tris-HCl pH 7.4, 50 mM reduced L-glutathione, 150 mM NaCl, 1 mM DTT, 1 mM EDTA) or His elution buffer (25 mM Tris pH 8.0, 100 mM NaCl, 200 mM Imidazole) on a thermal mixer at 37°C and 600 RPM for 30 mins. While the typical yield of enriched endogenous nucleosomes is highly reader domain-dependent (Typical yields of CAP-endogenous nucleosome complexes range between 50-100 μg of total protein as determined by Pierce BCA (*Thermo Fisher Scientific* 23225). Samples were adjusted to a final concentration of 1 µM for sufficient MS signal.

**Captify™ Luminex binding assays.** Assays to examine the interaction of tandem reader domains (the Queries) with biotinylated PTM-defined nucleosomes (the Targets) in multiplex Luminex panels were performed in 96-well plates (*GreinerBio* 655900) as previously.^5,6^All bead handling was performed under subdued lighting, with continuous mixing to ensure monodispersion during incubations, and magnetic capture for wash steps.

Avidin-conjugated MagPlex^®^ beads (*Luminex, Diasorin*) with spectrally distinct regions were used to assemble a multiplex panel with fully-defined biotinylated homo- and hetero- typic nucleosomes (**Figure 5d-e and S1**) as previously.^5,6^ Balance, nucleosome integrity and identity (Nucleosome : bead region) within the panel were confirmed using anti-dsDNA (*EMD Millipore* MAB030; 1/5, 1/50 and 1/500), anti-histone (*MilliporeSigma* MAB3422), anti-H3.1/2 (*Active Motif* 61629) and an appropriate anti-PTM if available.

For each analysis, 50 μL multiplexed bead panel (20,000 beads / mL / region; 1,000 beads / region) was combined with 50 μL tandem reader (concentrations as in **Figure 5d-e and S1**) in assay buffer (20 mM Tris pH 7.5, 250 mM NaCl, 0.01% BSA, 0.01% Tween-20, 1 mM DTT). The reaction plate was incubated for 60 mins, and beads washed for three cycles using 100 μL of assay buffer shaking for two mins between cycles. 100 μL of anti-Tag antibody [1:2,000 anti-GST (*Fortis* A190-122A)] was added to each well, incubated for 30 mins, and beads washed for three cycles. Anti-IgG PE [anti-Rabbit (*Biolegend* 406421)] was diluted 1:100 in assay buffer, added to each well, incubated for 30 mins, and beads washed for three cycles. Lastly, beads were resuspended in 100 μL assay buffer and fluorescence measured using the FlexMap3D Instrument (*Diasorin*).  Binding curves [Query:Target] were plotted in GraphPad Prism 10 (v10.10.0) using a non-linear 4PL curve fit and EC_50_^rel^values computed for specific comparisons.

**Immunoblotting.** Immunoblotting was used to determine pulldowns of reader-endogenous nucleosome complexes. Primary antibodies were: anti-GST tag (*Invitrogen* MA4-004, 1:1,000), anti-6xHis tag (*Invitrogen* 37-2900) and Anti-histone H3 (*Cell Signaling Technologies* 4499S, 1:2,000). Secondary antibodies were: anti-Mouse-HRP (*Jackson ImmunoResearch* 115-035-044, 1:2,000) and anti-Rabbit-HRP (*Jackson ImmunoResearch* 111-035-003, 1:2,000).

**Native Mass Spectrometry.** Recombinant BPTF proteins were incubated with PTM-defined semi synthetic nucleosomes in equimolar concentrations (1 µM) in binding buffer (20 mM HEPES pH 7.5, 250 mM NaCl, 0.01% BSA, 0.01% NP-40, 1 mM DTT) for 1 hr at 4°C. The mixture was subsequently desalted, and buffer exchanged using 30-kDa molecular-weight-cut-off (MWCO) spin filters (*MilliporeSigma* UFC503096) into 150 mM Ammonium acetate (AmAc) prepared in LC/MS grade Optima water (*Thermo Fisher Scientific* AAB-W6-4).^7^ Briefly, spin filters were first equilibrated with 500 µL water, and spun for 3 mins at 10,000 x *g*. Samples were loaded into equilibrated spin filter and centrifuged at 10,000 x *g* for 10 mins at 4°C. The buffer exchange process was repeated up to ten times to allow sufficient removal of MS-incompatible components. In each stage, the solution was spun down to <100 µL, and the filter replenished up to 500 µL with 150 mM AmAc. Buffer exchange of reader-endogenous nucleosome complexes into 150 mM AmAc were performed under the same procedure. Samples were concentrated to 1 µM (a concentration that consistently yielded a strong MS signal) and loaded into static NSI tips (*Thermo Fisher Scientific* ES380) for native electrospray ionization using a NanoSpray Flex Ion Source (*Thermo Fisher Scientific*). For PTM-defined semi-synthetic nucleosome and BPTF binding experiments, Nuc-MS analysis was performed on an Orbitrap Q Exactive Ultra High Mass Range (UHMR) MS (*Thermo Fisher Scientific*).^8^ For analysis of chromatin-associated proteins (CAPs) or CAP-endogenous nuc complexes, Nuc-MS analysis was performed on an Orbitrap Ascend Tribrid MS (*Thermo Fisher Scientific*). In-depth details pertaining to instrumentation and MS/MS parameters is in **Tables S5-9**.

**Targeted Denatured Liquid Chromatography Top-Down Mass Spectrometry (dLC-TD-MS).** CAP-enriched nucleosomes were buffer exchanged into 1% formic acid in LC/MS grade Optima water resulting in the dissociation of CAP-nucleosome complexes prior to dLC-TD-MS analysis. This was performed on a Vanquish Neo UPLC (*Thermo Fisher Scientific*) coupled to an Orbitrap Eclipse Tribrid Mass Spectrometer (*Thermo Fisher Scientific*). Reverse-phase separation of histones was performed using MAbPac RP column (1500 Å, 4 µm, 150 µm x 150 mm) (*Thermo Fisher Scientific*, ES907) using a 60 minute gradient comprised of mobile phase A (0.01% (v/v) formic acid in LC/MS grade Optima water) and mobile phase B (80% (v/v) LC/MS grade Optima Acetonitrile, 20% (v/v) LC/MS grade Optima water and 0.01% (v/v) formic acid) with a gradient of: 0-5 mins at 5% B, 6-45 mins at 20% B, 46-54 mins at 99% B, and finally 56-60 mins at 5% B) with a constant flow rate of 1 µL/min).

For targeted MS/MS analysis by parallel reaction monitoring (PRM) of 3x methyl, 6x methyl, and 9x methyl equivalent H3.2 proteoforms, a custom MS workflow was designed. The following global parameters were used: Pressure Mode: Low pressure, Infusion Mode: Liquid Chromatography, Expected LC Peak Width (s): 30, Advanced Peak Determination: True, Detector type: Orbitrap, Orbitrap resolution: 120000, Use Quadrupole Isolation: True, Scan range (*m/z*): 500-200, RF lens (%): 60, Normalized AGC Target (%): 1000, Absolute AGC Value: 4E6, Maximum Injection Time (ms): 100, Microscan: 1, Polarity: positive, and Source fragmentation: 15 V

For tandem MS/MS parameters: MS^n^ level: 2, Isolation Mode: Quadrupole, Isolation window (m/z): 0.8, ETD reagent target: 5e5, Max ETD reagent injection time (ms): 200, ETD reaction time: 14 ms, ETD supplemental activation: true, SA collision energy (%): 35, Detector Type: 60000, Mass Range: Normal, RF lens (%): 60, Normalized AGC target (%): 2000, Absolute AGC value: 1e6, Maximum injection time (ms): 1200, Microscans: 1, and Source fragmentation: 15 V. A targeted mass list comprised of desired histone H3.2 proteoforms was used for PRM **(Table S10)**.

**Mass Spectrometry data analysis.** FreeStyle (*Thermo* *Fisher*, v1.8) was used to obtain time-dependent acquisition of MS spectra. UniDec (v 6.0.4)^9^ was used to generate deconvoluted MS1 spectra. For data processing the following settings were used: *m/z* 4000-12000. For UniDec parameters the following settings were used: Charge range: 1-50+, Mass range: 5000-350,000 kDa, Sample mass every 10 Da, Smooth Nearby Points: Some, Suppress Artifacts: None, Peak FWHM: 0.85, Charge Smooth width: 1.0, Point Smooth Width: 1.0, and Maximum # of iterations: 100. TDValidator (*Proteinaceous*, v1.1.242332.1) was used to characterize intact masses of histone subunits at isotopically resolution (MS1) and fragmentation data of histones and reader proteins (MS2).^10^ The following TDValidator Precursor parameters for intact mass analysis were as follows: Max PPM Tolerance: 20.00, Sub PPM Tolerance: 20.00, Cluster Tolerance: 0.70, Charge Range: 5-50, Minimum Score: 0.30, signal-to-noise cutoff: 3.00, Mercury7 Limit: 0.0001, and Isotopically Resolved. Relative quantification of intact histone proteoforms were performed by extracting signal intensities (MS1) at intact precursor level and comparisons of histone proteoform between bulk HeLa and reader-enriched nucleosomes were performed using the same charge states (z). The following TDValidator Fragments parameters for high resolution fragmentation data were as follows: Max PPM tolerance: 10-15 ppm, Sub PPM tolerance: 5 ppm, Charge Range: 1-15, Minimum Score: 0.35-0.70, signal-to-noise cutoff: 3.00, Minimum Size: 1, Mercury7 Limit: 0.0001, and FDR Decoy Runs: 1000. ProSight Lite (*Proteinaceous*, v1.4) and TDValidator were used to match *b* and *y*, or *c* and *z* fragment ions corresponding to histone or reader sequences and generate P Scores for each histone proteoform. All mass spectrometry .raw files were uploaded to MassIVE (MSV000097336).

**Relative quantification of histone proteoforms enriched by CAPs.** Quantification of enrichment was calculated by determining the relative abundance of each histone proteoform based on intact mass. Each histone proteoform was normalized to either H3.2: 3xMe (at z = +17), H4: N-Acetyl + K20me2 (at z = +14) or H2A Type 2A (at z = +14). The relative ratio of CAP:HeLa bulk is the normalized values of each histone proteoform enriched by CAP divided by that in bulk HeLa.

**Relative quantification of histone H3.2 proteoforms enriched by CAPs.** Relative quantification of histone H3.2 proteoforms especially isobaric forms (*i.e.*, proteoforms with combinatorial PTMs that equate to the same mass) is particularly challenging due to spectral complexity. To address this, we conducted relative quantification using Proteoform Finder – a function in ProSight Native software tool (*Proteinaceous*, v1.0.24103).^11,12^ The function is described in greater detail elsewhere,^12^ but in brief provides relative quantification from a user-defined list of proteoforms based on fragment ions from tandem MS/MS fragmentation information from PRM-based MS experiments. Fragment ions are fitted against a theoretical isotopic distribution, and assigned a fit score between 0-1, where 1 represents a perfect match to the theoretical isotopic distribution though scores > 0.4 are considered well matched. Fragment ions that passed a minimum score cut off (≥ 0.4) were used to determine XIC (Extracted Ion Chromatogram) and AUC (Area under curve) of a histone proteoform, representing relative abundance. Proteoform Detection parameters used: Min Spectra Detected: 2, Global Time Min 0.00, Global Time Max (1000.00), Min Charge State: 1, Max Charge State: 25, Min Fit Score: 0.4, *m/z* Error Tolerance (ppm): 10.00, *m/z*: 3.00, Minimum Intensity: 100, Isotopomer Intensity Tolerance: 0.35, Max Isotopomers: 7, Min S/N: 1.00. Given the high level of complexity of combinatorial PTMs present in H3.2 proteoforms, a higher confidence list - where spectra were manually validated - was included in the Proteoform Finder analysis. Only *b* and *c* fragment ions located between histone H3.2 aa1-40 sequence (K4, K14, K18, K23, K27, K37, and K38) were used for the calculation of each H3.2 proteoform XIC and AUC. Percent relative abundances of each H3.2 proteoform was calculated by dividing each histone proteoform AUC by the total AUC of all histone proteoforms present.

**Safety Statement:** No unexpected or unusually high safety hazards were encountered.

**Additional References**

(1) Marunde, M. R.; Fuchs, H. A.; Burg, J. M.; Popova, I. K.; Vaidya, A.; Hall, N. W.; Weinzapfel, E. N.; Meiners, M. J.; Watson, R.; Gillespie, Z. B.; Taylor, H. F.; Mukhsinova, L.; Onuoha, U. C.; Howard, S. A.; Novitzky, K.; McAnarney, E. T.; Krajewski, K.; Cowles, M. W.; Cheek, M. A.; Sun, Z.-W.; Venters, B. J.; Keogh, M.-C.; Musselman, C. A. Nucleosome Conformation Dictates the Histone Code. *Elife* 2024, *13*. https://doi.org/10.7554/eLife.78866.

(2) Shah, R. N.; Grzybowski, A. T.; Cornett, E. M.; Johnstone, A. L.; Dickson, B. M.; Boone, B. A.; Cheek, M. A.; Cowles, M. W.; Maryanski, D.; Meiners, M. J.; Tiedemann, R. L.; Vaughan, R. M.; Arora, N.; Sun, Z. W.; Rothbart, S. B.; Keogh, M. C.; Ruthenburg, A. J. Examining the Roles of H3K4 Methylation States with Systematically Characterized Antibodies. *Mol Cell* 2018, *72* (1), 162-177.e7. https://doi.org/10.1016/j.molcel.2018.08.015.

(3) Marunde, M. R.; Popova, I. K.; Weinzapfel, E. N.; Keogh, M. C. The DCypher Approach to Interrogate Chromatin Reader Activity Against Posttranslational Modification-Defined Histone Peptides and Nucleosomes. In *Methods in Molecular Biology*; Humana Press Inc., 2022; Vol. 2458, pp 231–255. https://doi.org/10.1007/978-1-0716-2140-0_13.

(4) Keogh, M.-C.; Almouzni, G.; Andrews, A. J.; Armache, K.-J.; Arrowsmith, C. H.; Baek, S. H.; Bedford, M. T.; Bernstein, E.; Côté, J.; David, Y.; Denu, J. M.; Fierz, B.; Garcia, B. A.; Glass, K. C.; Gozani, O.; Helin, K.; Henikoff, S.; Jensen, O. N.; Josefowicz, S. Z.; Kelleher, N. L.; Kutateladze, T. G.; Lindner, H. H.; Lu, C.; Luger, K.; Mallick, P.; Musselman, C. A.; Muir, T. W.; Paša-Tolić, L.; Schneider, R.; Shi, X.; Shi, Y.; Sidoli, S.; Smith, L. M.; Tyler, J. K.; Wolberger, C.; Workman, J. L.; Strahl, B. D.; Young, N. L. A Needed Nomenclature for Nucleosomes. *Mol Cell* 2025, *85* (19), 3554–3561. https://doi.org/10.1016/j.molcel.2025.08.029.

(5) Hicks, C. W.; Rahman, S.; Gloor, S. L.; Fields, J. K.; Husby, N. L.; Vaidya, A.; Maier, K. E.; Morgan, M.; Keogh, M. C.; Wolberger, C. Ubiquitinated Histone H2B as Gatekeeper of the Nucleosome Acidic Patch. *Nucleic Acids Res* 2024, *52* (16), 9978–9995. https://doi.org/10.1093/nar/gkae698.

(6) Marunde, M. R.; Popova, I. K.; Hall, N. W.; Vaidya, A.; Bone, J. R.; Boone, B. A.; Brown, P. J.; Ezell, R. J.; Firestone, T. M.; Fuchs, H. A.; Gibson, E.; Gillespie, Z. B.; Gloor, S. L.; Hickman, A. R.; Howard, S. A.; Husby, N. L.; Hsiung, V. T.; Johnstone, A. L.; Khan, L. F.; Krajewski, K.; Lee, A. S.; McAnarney, E. T.; Maier, K. E.; Maryanski, D. N.; McCuiston, J. L.; Noll, K. E.; Novitzky, K.; Patteson, E. F.; Rodriguez, K. L.; Sanchez, J. C.; Schachner, L. F.; Smith, C. E.; Sun, L.; Taylor, H. F.; Watson, R.; Willis, H. E.; Musselman, C. A.; Venters, B. J.; Cheek, M. A.; Meiners, M. J.; Sun, Z.-W.; Kelleher, N. L.; Cowles, M. W.; Weinzapfel, E. N.; Keogh, M.-C.; Burg, J. M. Nucleosome Context Regulates Chromatin Reader Preference. *Nucleic Acids Res* 2025, *53* (20). https://doi.org/10.1093/nar/gkaf1061.

(7) Jooß, K.; McGee, J. P.; Melani, R. D.; Kelleher, N. L. Standard Procedures for Native CZE-MS of Proteins and Protein Complexes up to 800 KDa. *Electrophoresis* 2021, *42* (9–10), 1050–1059. https://doi.org/10.1002/elps.202000317.

(8) Schachner, L. F.; Ives, A. N.; McGee, J. P.; Melani, R. D.; Kafader, J. O.; Compton, P. D.; Patrie, S. M.; Kelleher, N. L. Standard Proteoforms and Their Complexes for Native Mass Spectrometry. *J Am Soc Mass Spectrom* 2019, *30* (7), 1190–1198. https://doi.org/10.1007/s13361-019-02191-w.

(9) Phung, W.; Bakalarski, C. E.; Hinkle, T. B.; Sandoval, W.; Marty, M. T. UniDec Processing Pipeline for Rapid Analysis of Biotherapeutic Mass Spectrometry Data. *Anal Chem* 2023, *95* (30), 11491–11498. https://doi.org/10.1021/acs.analchem.3c02010.

(10) Fornelli, L.; Srzentić, K.; Huguet, R.; Mullen, C.; Sharma, S.; Zabrouskov, V.; Fellers, R. T.; Durbin, K. R.; Compton, P. D.; Kelleher, N. L. Accurate Sequence Analysis of a Monoclonal Antibody by Top-Down and Middle-Down Orbitrap Mass Spectrometry Applying Multiple Ion Activation Techniques. *Anal Chem* 2018, *90* (14), 8421–8429. https://doi.org/10.1021/acs.analchem.8b00984.

(11) Durbin, K. R.; Robey, M. T.; Voong, L. N.; Fellers, R. T.; Lutomski, C. A.; El-Baba, T. J.; Robinson, C. V; Kelleher, N. L. ProSight Native: Defining Protein Complex Composition from Native Top-Down Mass Spectrometry Data. *J Proteome Res* 2023, *22* (8), 2660–2668. https://doi.org/10.1021/acs.jproteome.3c00171.

(12) Huang, C.-F.; Kline, J. T.; Negrão, F.; Robey, M. T.; Toby, T. K.; Durbin, K. R.; Fellers, R. T.; Friedewald, J. J.; Levitsky, J.; Abecassis, M. M. I.; Melani, R. D.; Kelleher, N. L.; Fornelli, L. Targeted Quantification of Proteoforms in Complex Samples by Proteoform Reaction Monitoring. *Anal Chem* 2024, *96* (8), 3578–3586. https://doi.org/10.1021/acs.analchem.3c05578.


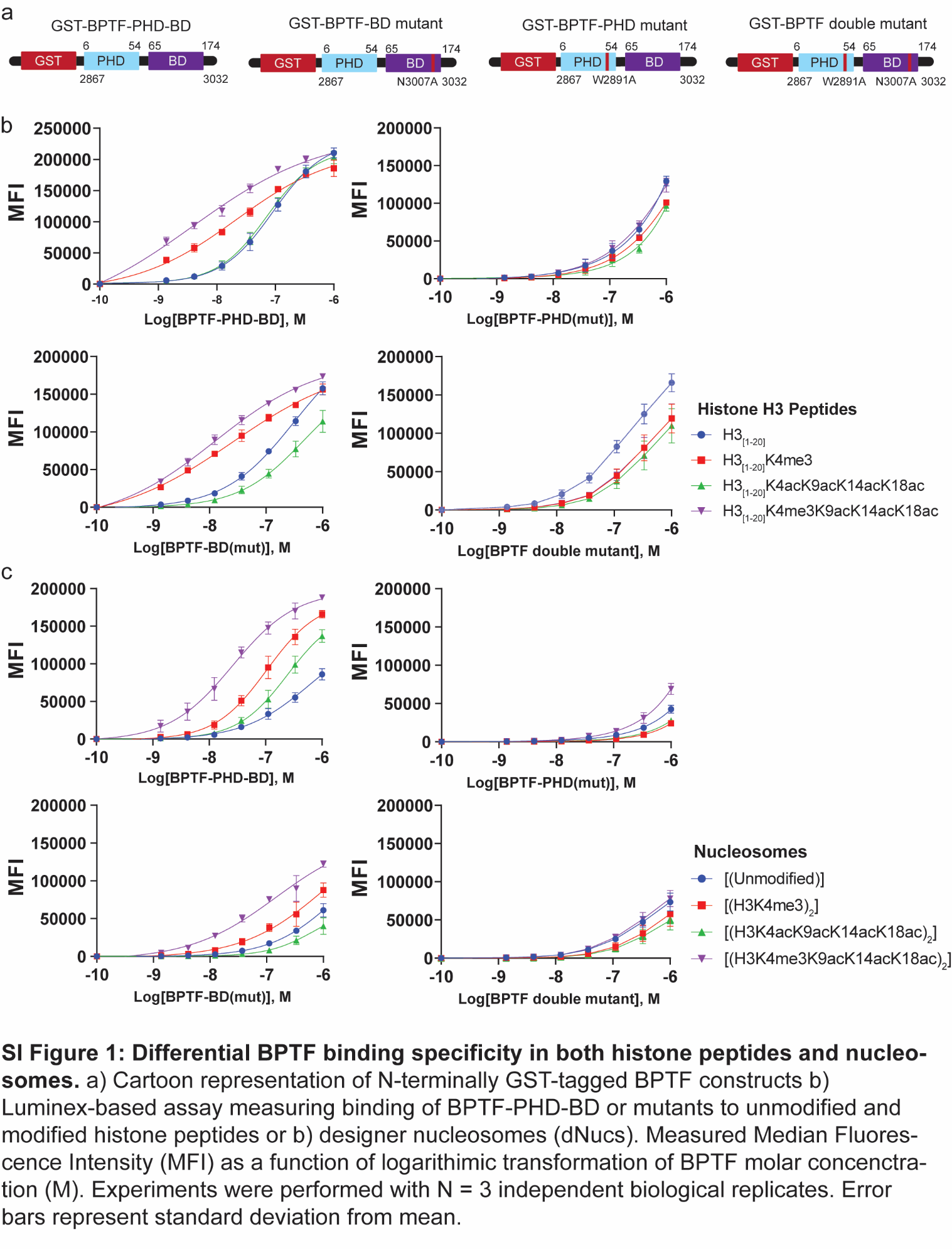


**Figure S1: Differential BPTF PHD-BD binding preference for histone peptides and nucleosomes.** **a)** N-terminally GST-tagged BPTF (Uniprot Q12830) constructs (including domain coverage and mutant position). **b-c)** Binding (by Captify Luminex assay) of each BPTF-PHD-BD (titrated Queries) to PTM-defined histone peptides **(b)** or semi-synthetic nucleosomes **(c)** (each fixed concentration Targets). Data is presented as Measured Median Fluorescence Intensity (MFI) as a function of logarithmic transformation of BPTF molar concentration (M). Experiments were performed with three independent biological replicates. Error bars represent standard deviation from mean.


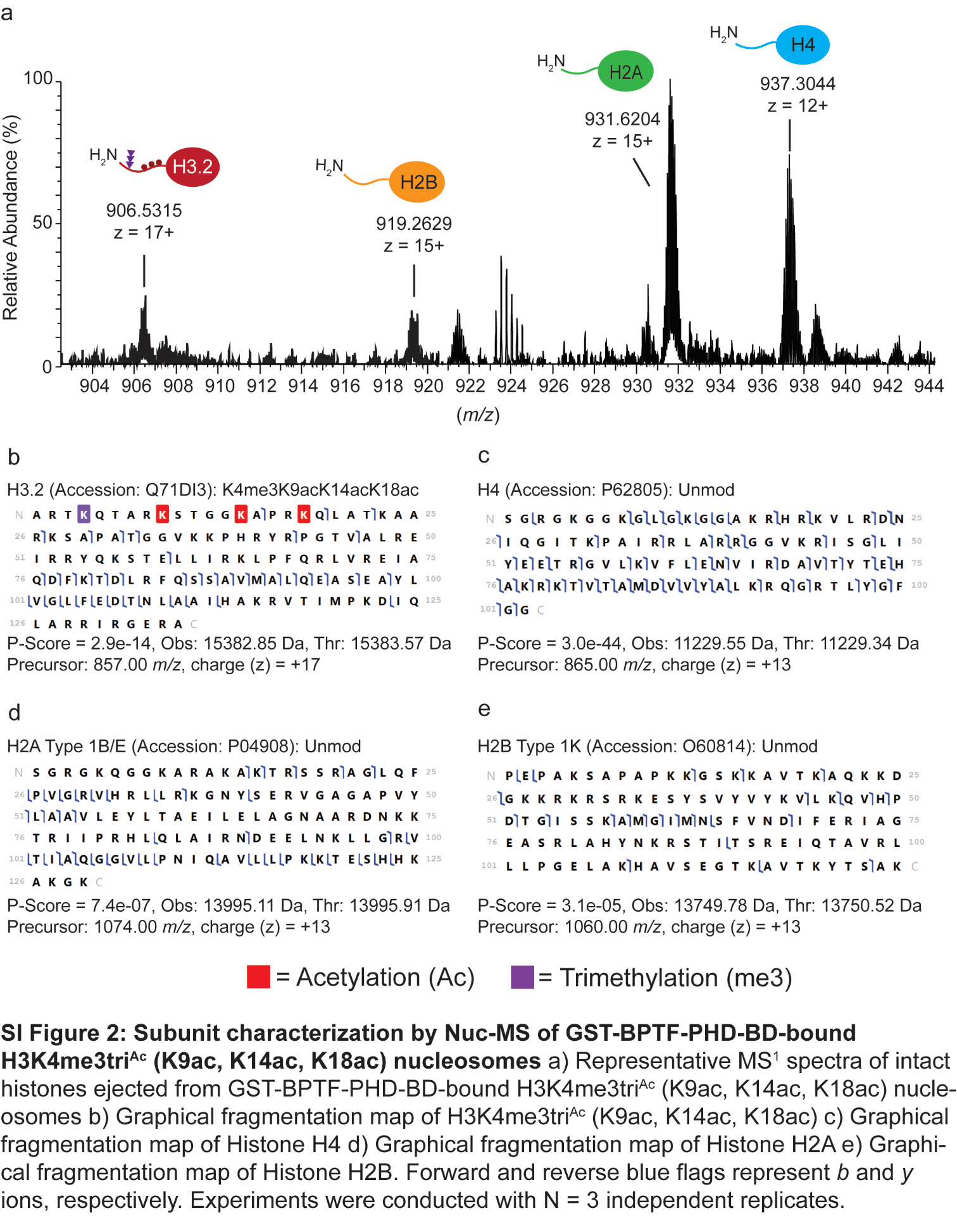


**Figure S2: Nuc-MS characterization of BPTF-bound ([H3K4me3K9acK14acK18ac]_2_) nucleosomes. a)** Representative MS1 spectra of intact histones ejected from GST-BPTF PHD-BD-bound ([H3K4me3K9acK14acK18ac]_2_) nucleosomes. **b-e)** Graphical fragment maps of the most abundant intact histone precursor ions: H3K4me3K9acK14acK18ac **(b)**, H4 **(c)**, H2A Type 1B/E **(d)**, and H2B Type 1K **(e)**. Forward and reverse blue flags respectively represent b and y ions resulting from higher-energy collisional dissociation (HCD). Experiments were conducted with three independent biological replicates. Fragments were manually validated using TDValidator and corresponding P-Scores were calculated using ProSight Lite.


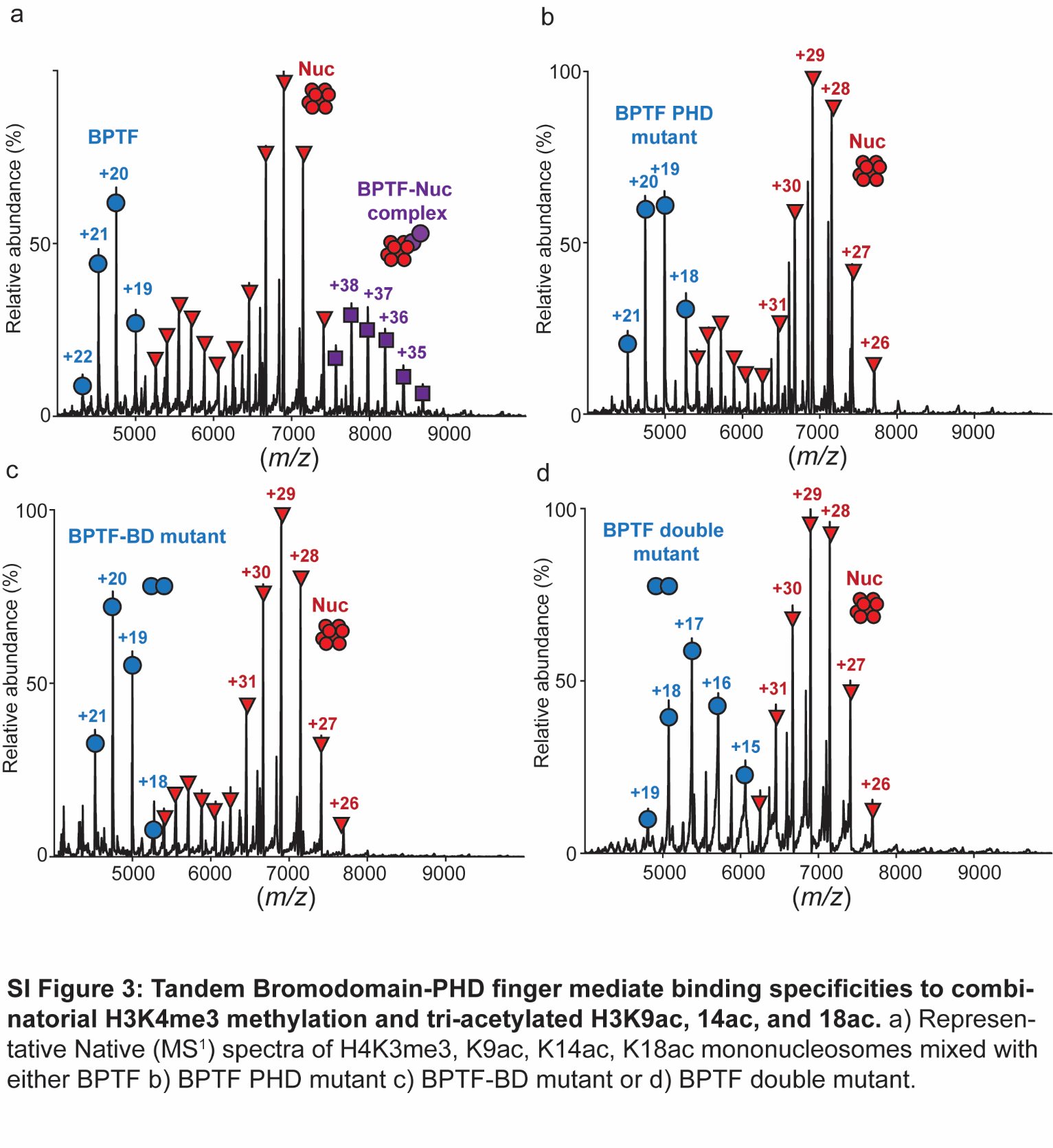


**Figure S3: Both domains in the BPTF PHD-BD tandem are required for effective binding to ([H3K4me3K9acK14acK18ac]_2_) nucleosomes. a-d)** Representative Native MS1 spectra of ([H3K4me3K9acK14acK18ac]_2_) mononucleosomes mixed with either BPTF PHD-BD **(a)** or the loss-of-function mutants PHD* **(b)**, BD* **(c)** or PHD*BD* **(d)**. Experiments were conducted with three independent biological replicates.


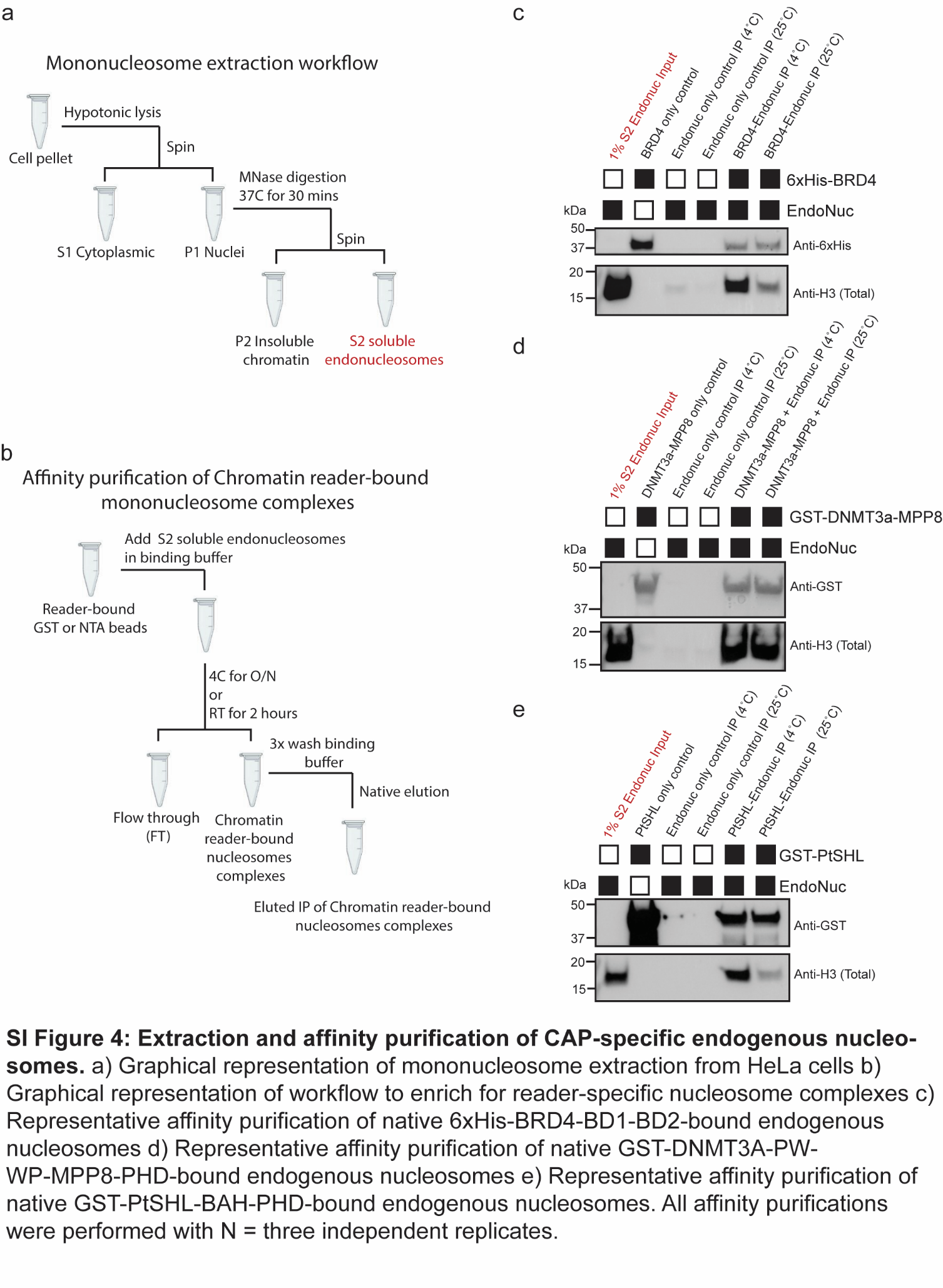


**Figure S4: Extraction and affinity purification of CAPs complexed with endogenous nucleosomes. a)** Graphical representation of mononucleosome extraction from HeLa cells. **b)** Graphical representation of workflow to enrich for CAP:nucleosome (CAP:nuc) complexes. **c-e)** Representative affinity purification of tandem reader bound endogenous nucleosomes: via 6xHis-BRD4 BD1-BD2 **(c)**, GST-DNMT3A-MPP8 PWWP -PHD **(d)**, or GST-PtSHL BAH-PHD **(e)**. All affinity purifications were performed with three independent biological replicates.


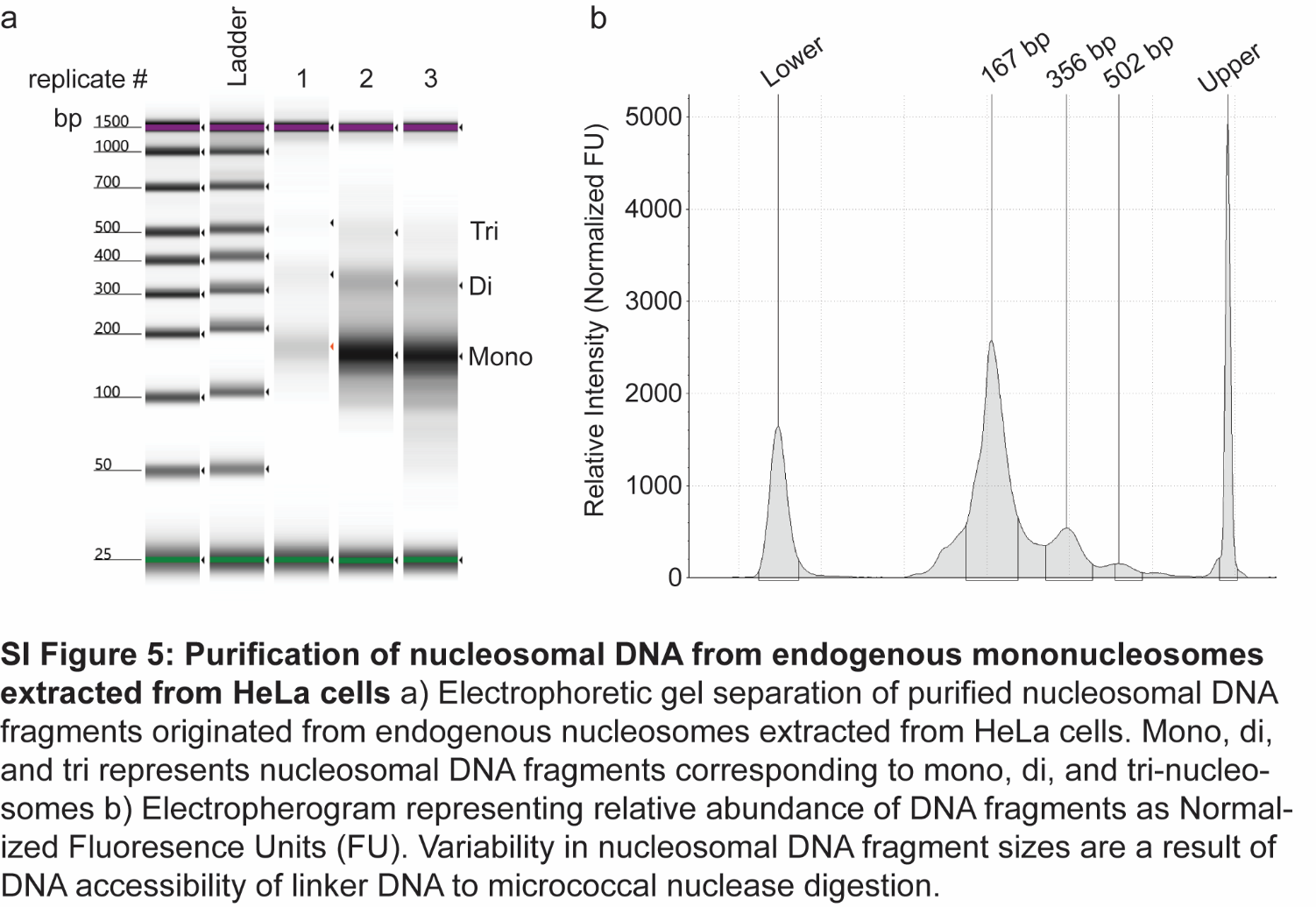


**Figure S5: Purification of nucleosomal DNA from endogenous HeLa mononucleosomes. a)** Electrophoretic gel separation of purified nucleosomal DNA fragments originated from endogenous nucleosomes (mono-, di- and tri-) extracted from HeLa cells. **b)** Relative abundance of DNA fragments as Normalized Fluoresence Units (FU).


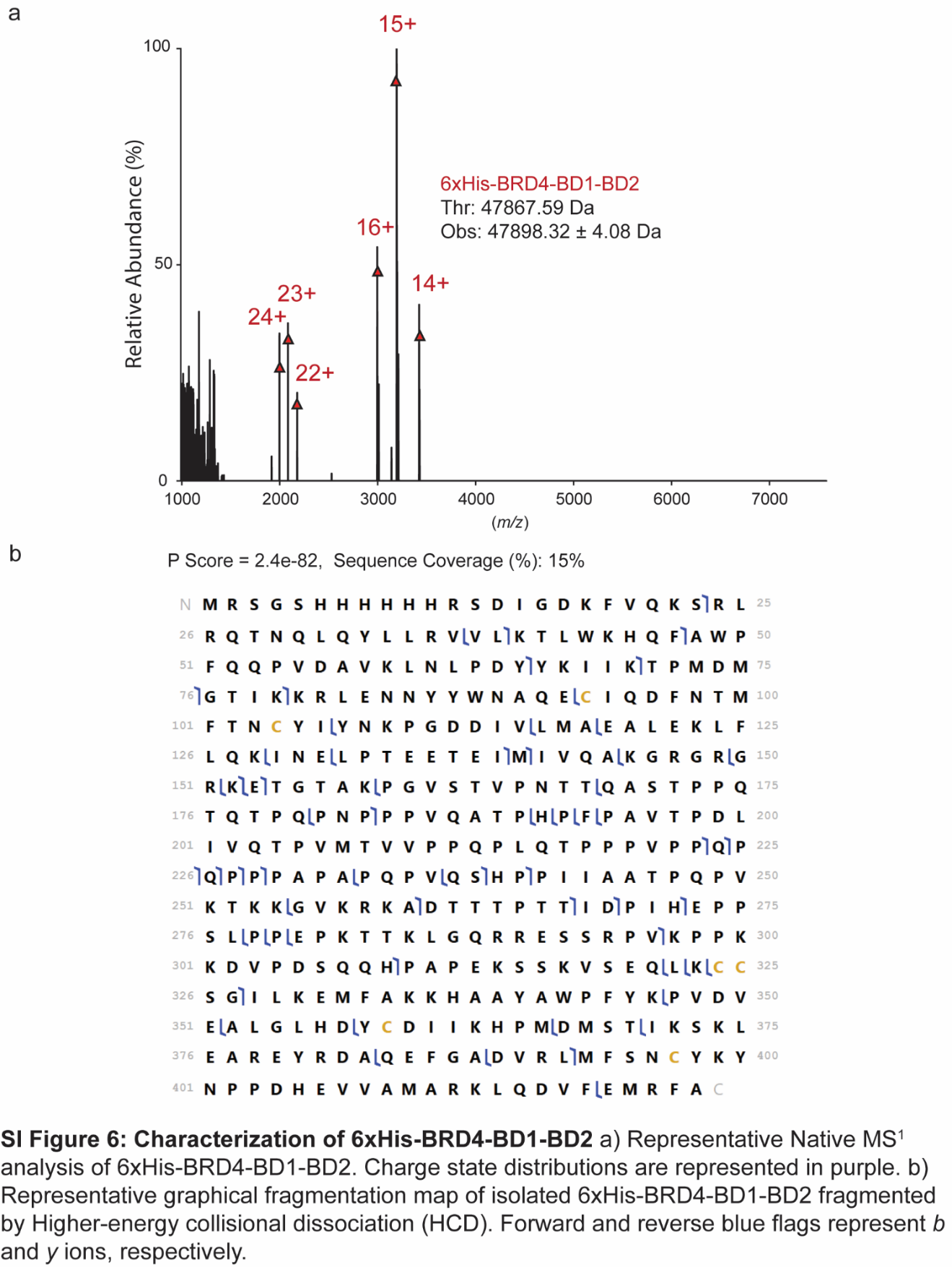


**Figure S6: Characterization of 6xHis-BRD4 BD1-BD2 native tandem reader. a)** Representative Native MS1 analysis of 6xHis-BRD4 BD1-BD2. Charge state distributions of intact 6xHis-BRD4 BD1-BD2 are represented in red (+14 to +16) and (+22 to +24). **b)** Representative graphical fragment map of isolated 6xHis-BRD4 BD1-BD2 (precursor ion: 3141.00 m/z) fragmented by higher-energy collisional dissociation (HCD). Forward and reverse blue flags respectively represent b and y ions. Experiments were conducted with three independent biological replicates. Fragments were manually validated using TDValidator and corresponding P-scores calculated using ProSight Lite.


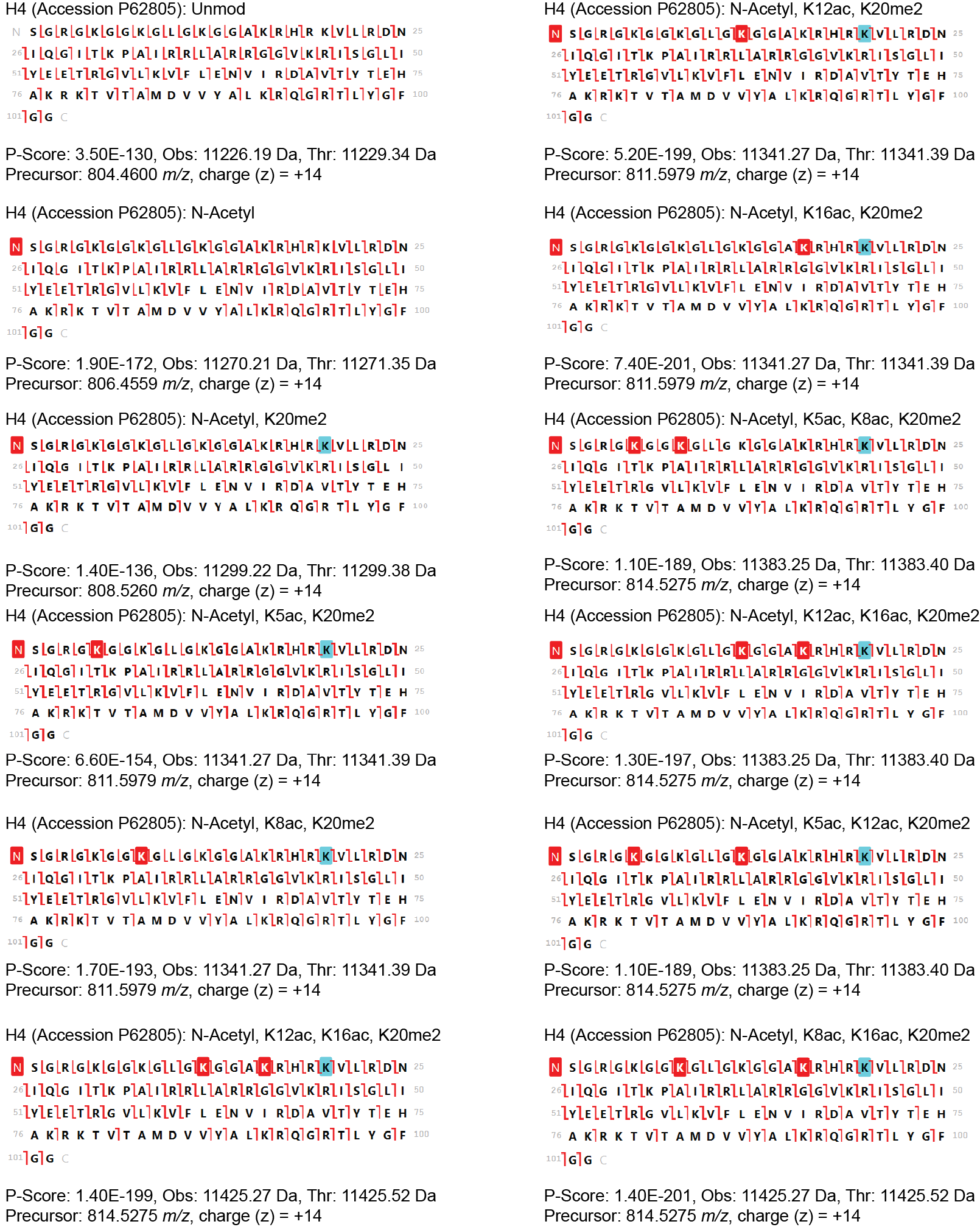


**Figure S7: Characterization of H4 proteoforms from BRD4-enriched endogenous nucleosomes.** Representative graphical fragment maps of tandem MS fragmentation of histone H4 proteoforms in BRD4 enriched nucleosomes. Tandem MS fragmentation was performed using electron-transfer dissociation (ETD) of isolated H4 proteoforms (precursor ions (m/z) used at charge state (z) +14 represent the isolated intact H4 proteoforms used for tandem MS). Forward and reverse red flags respectively represent c and z ions. Measurements were performed with three independent biological replicates. Observed (Obs) and theoretical (Thr) are represented as monoisotopic masses (Da). Fragments were manually validated using TDValidator and corresponding P-scores calculated using ProSight Lite.


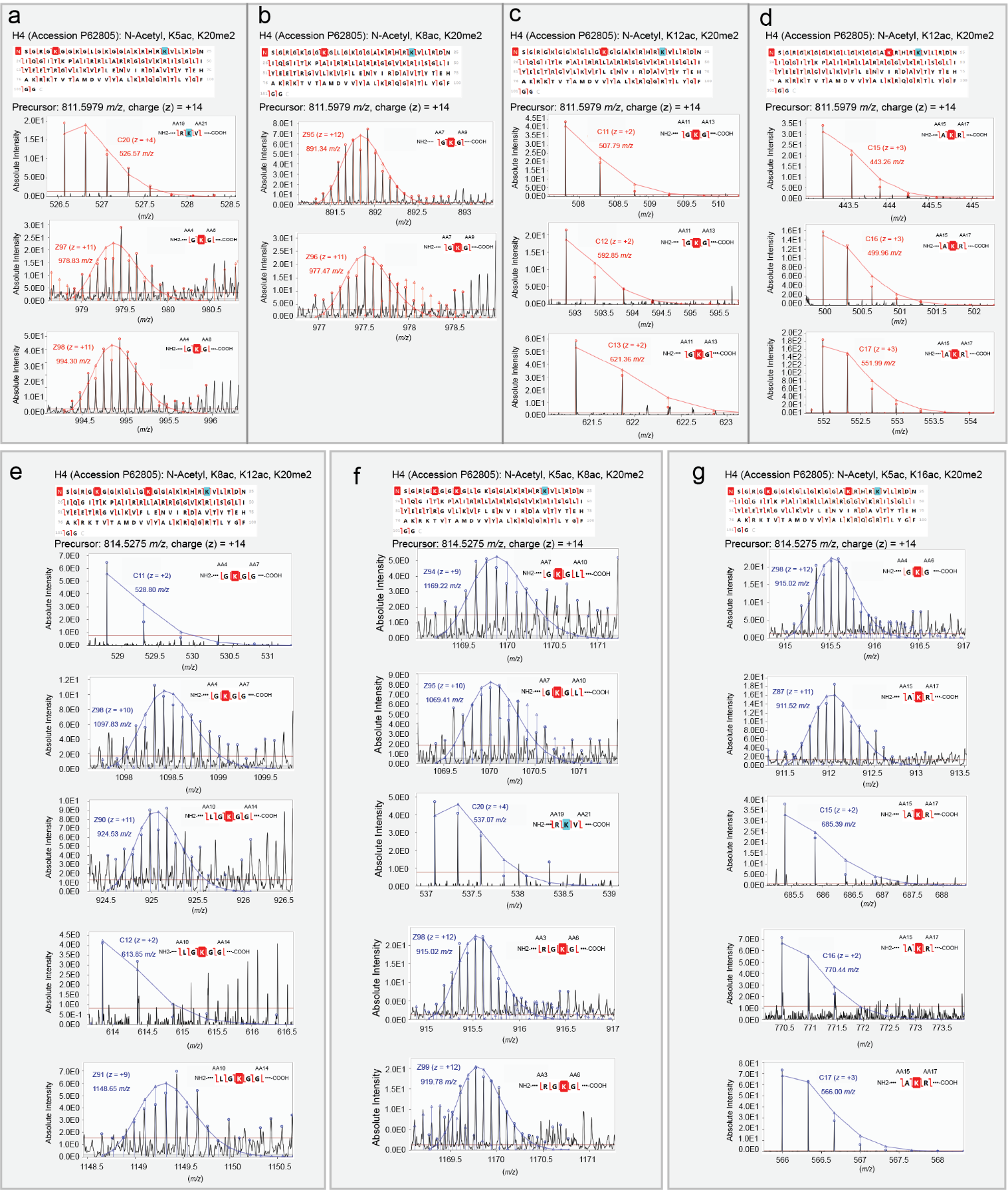


**Figure S8: Tandem MS characterization of H4 proteoforms from BRD4-enriched endogenous nucleosomes.** Representative tandem MS fragmentation spectra of the following H4 proteoforms: **a)** {H4K5acK20me2}, **b)** {H4K8acK20me2}, **c)** {H4K12acK20me2}, **d)** {H4K16acK20me2}, **e)** {H4K5acK12acK20me2}, **f)** {H4K5acK8acK20me2}, and **g)** {H4K5acK16acK20me2} in BRD4 enriched nucleosomes. Tandem MS fragmentation was performed using electron-transfer dissociation (ETD) of H4 proteoforms (precursor ions (m/z) used at charge state (z) +14 represent the isolated intact H4 proteoforms used for tandem MS). Forward and reverse red flags respectively represent c and z ions. Measurements were performed with three independent biological replicates. Observed (Obs) and theoretical (Thr) are represented as monoisotopic masses (Da).


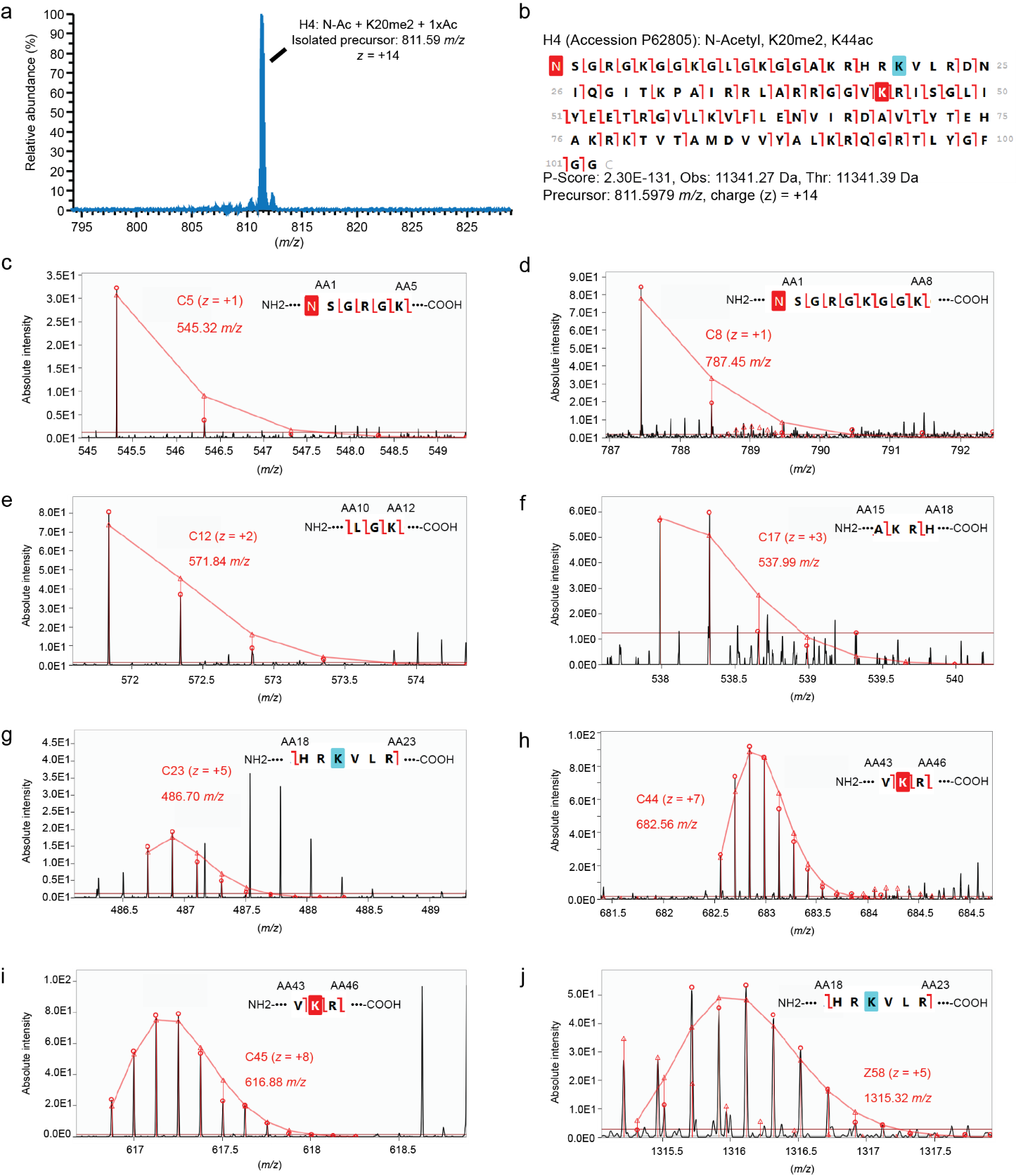


**Figure S9: Tandem MS characterization of {H4K20me2K44ac} proteoform from BRD4-enriched endogenous nucleosomes. a)** Representative MS spectrum of isolated H4: N-Acetyl + K20me2 + 1x Ac using 811.59 m/z precursor ion with charge state (z) = +14 for tandem MS. **b)** Representative graphical fragment map of tandem MS fragmentation of {H4K20me2K44ac} proteoform. **c-j)** Representative tandem MS fragmentation spectra of {H4K20me2K44ac}. Tandem MS fragmentation was performed using electron-transfer dissociation (ETD) of H4: N-Acetyl + K20me2 + 1x Ac (precursor ion (m/z) used at charge state (z) +14 represent the isolated intact H4 proteoform used for tandem MS). Forward and reverse red flags respectively represent c and z ions. Measurements were performed with three independent biological replicates. Observed (Obs) and theoretical (Thr) are represented as monoisotopic masses (Da).


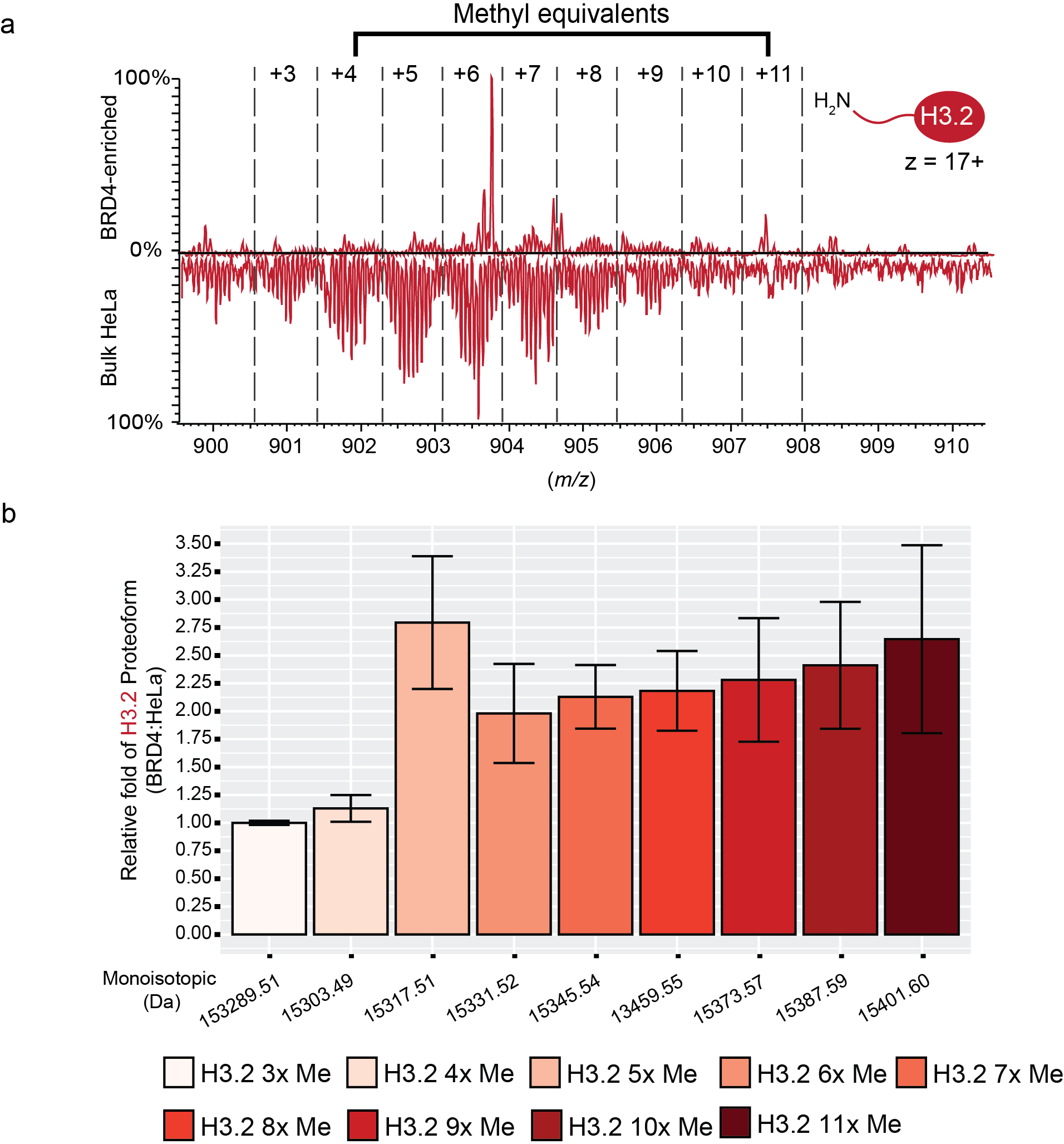


**Figure S10: Relative quantification of H3.2 proteoforms from BRD4-enriched endogenous nucleosomes. a-b)** Representative MS1 spectra **(a)** and quantification **(b)** of H3.2 proteoforms. Methyl equivalents are represented as (nxMe). Quantification of enrichment was calculated by determining the relative abundances of each histone proteoform and normalizing to H3.2: 3xMe. The relative ratio of BRD4:HeLa bulk is the normalized values of histone proteoform enriched by BRD4 divided by histone H3.2 proteoform in HeLa bulk. Error bars represent standard deviation from the mean.


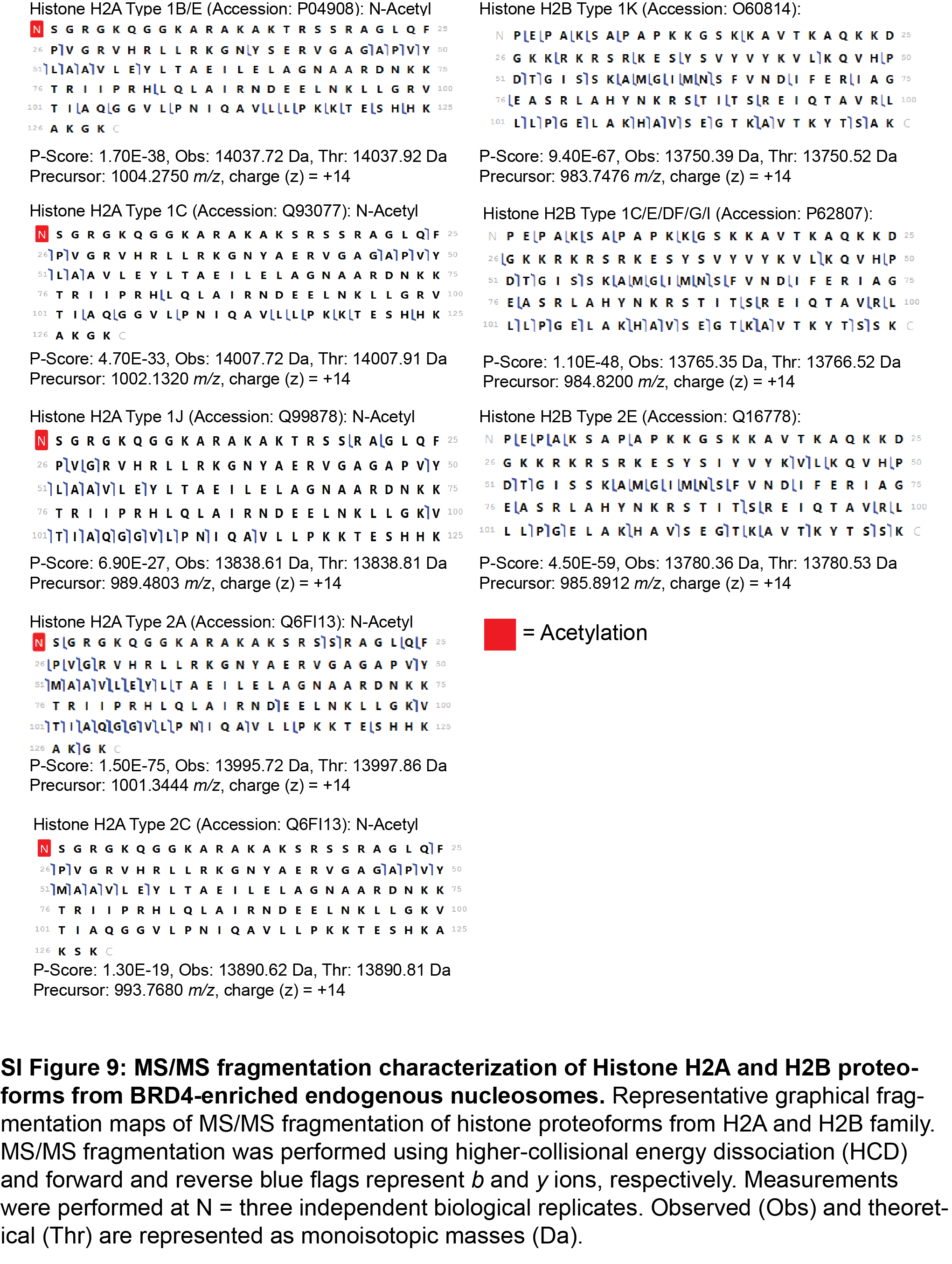


**Figure S11: Characterization of H2A and H2B proteoforms from BRD4-enriched endogenous nucleosomes.** Representative graphical fragment maps of tandem MS fragmentation of H2A and H2B proteoforms in BRD4 enriched nucleosomes. Tandem MS fragmentation was performed using higher-collisional energy dissociation (HCD) across a distribution of H2A and H2B proteoforms (precursor ions (m/z) at charge state (z) +14 represent the intact H2A and H2B proteoforms used for tandem MS). Forward and reverse blue flags respectively represent b and y ions. Measurements were performed with three independent biological replicates. Observed (Obs) and theoretical (Thr) are represented as monoisotopic masses (Da). Fragments were manually validated using TDValidator and corresponding P-scores were calculated using ProSight Lite.


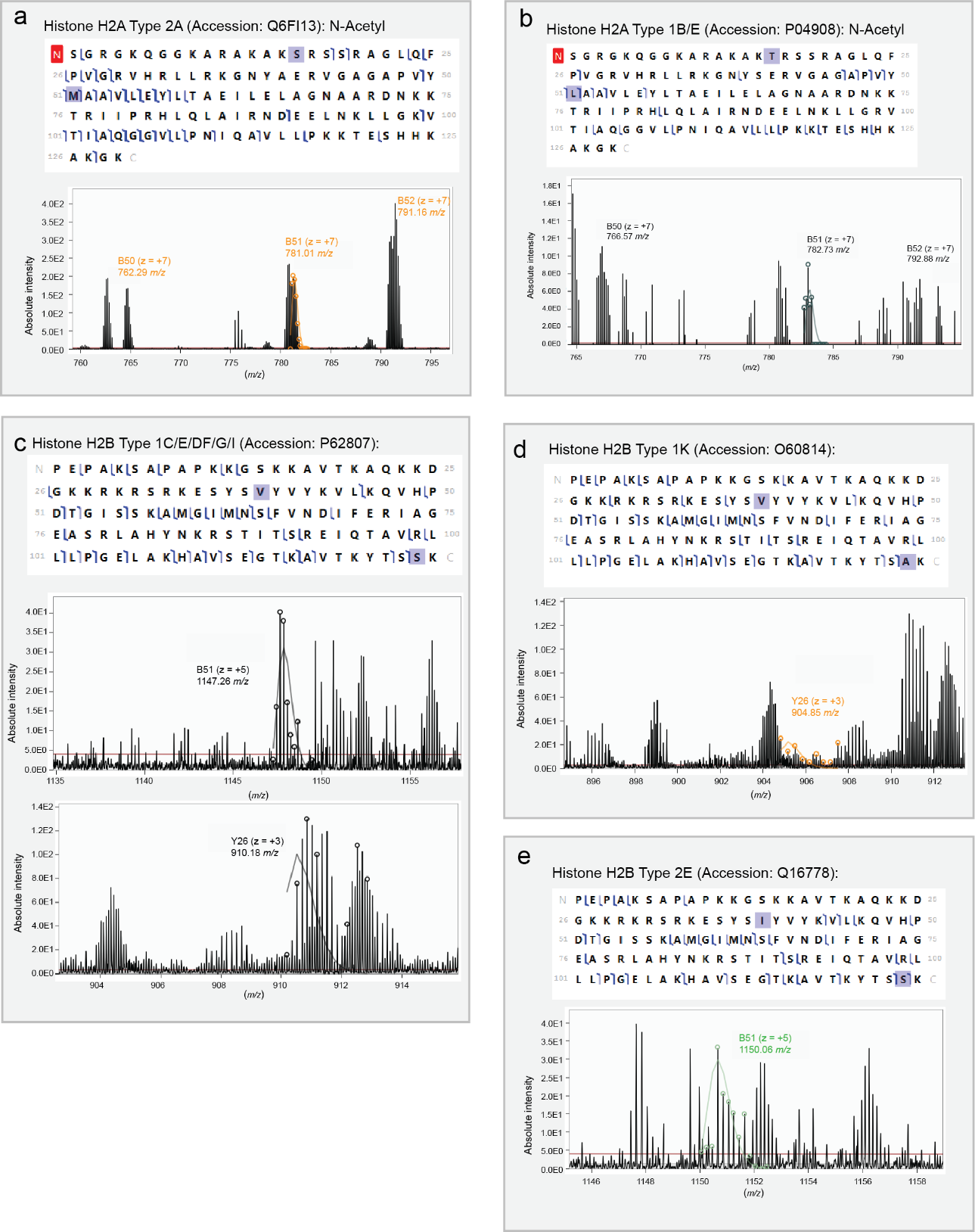


**Figure S12: Tandem MS characterization of H2A and H2B proteoforms from BRD4-enriched endogenous nucleosomes.** Representative graphical fragment maps and tandem MS fragmentation spectra of **a)** H2A Type 2A, **b)** H2A Type 1B/E, **c)** H2B Type 1C/E/F/G/I, **d)** H2B Type 1K, and **e)** H2B Type 2E proteoforms in BRD4 enriched nucleosomes. Tandem MS fragmentation was performed using higher-collisional energy dissociation (HCD) across a distribution of H2A and H2B proteoforms (precursor ions (m/z) at charge state (z) +14 represent the intact H2A and H2B proteoforms used for tandem MS). Forward and reverse blue flags respectively represent b and y ions. Measurements were performed with three independent biological replicates. Observed (Obs) and theoretical (Thr) are represented as monoisotopic masses (Da). Purple shade represents single amino acid differences.


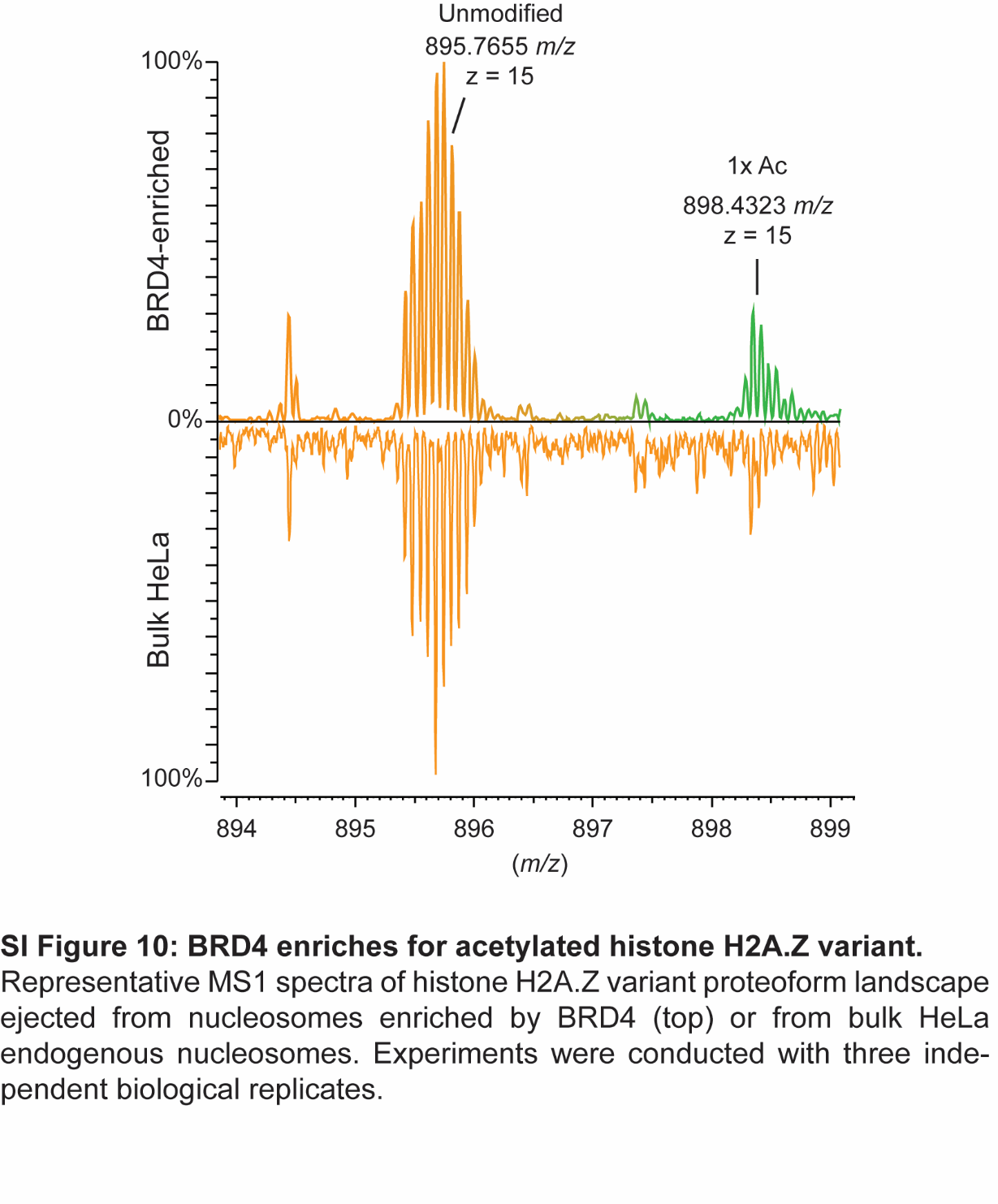


**Figure S13: BRD4 enriches for acetylated histone variant H2A.Z.** Representative MS1 spectrum of intact H2A.Z proteoform landscape ejected from nucleosomes enriched by BRD4 (top) or from bulk HeLa endogenous nucleosomes (bottom). Highlighted in green is an acetylated H2A.Z. Experiments were conducted with three independent biological replicates.


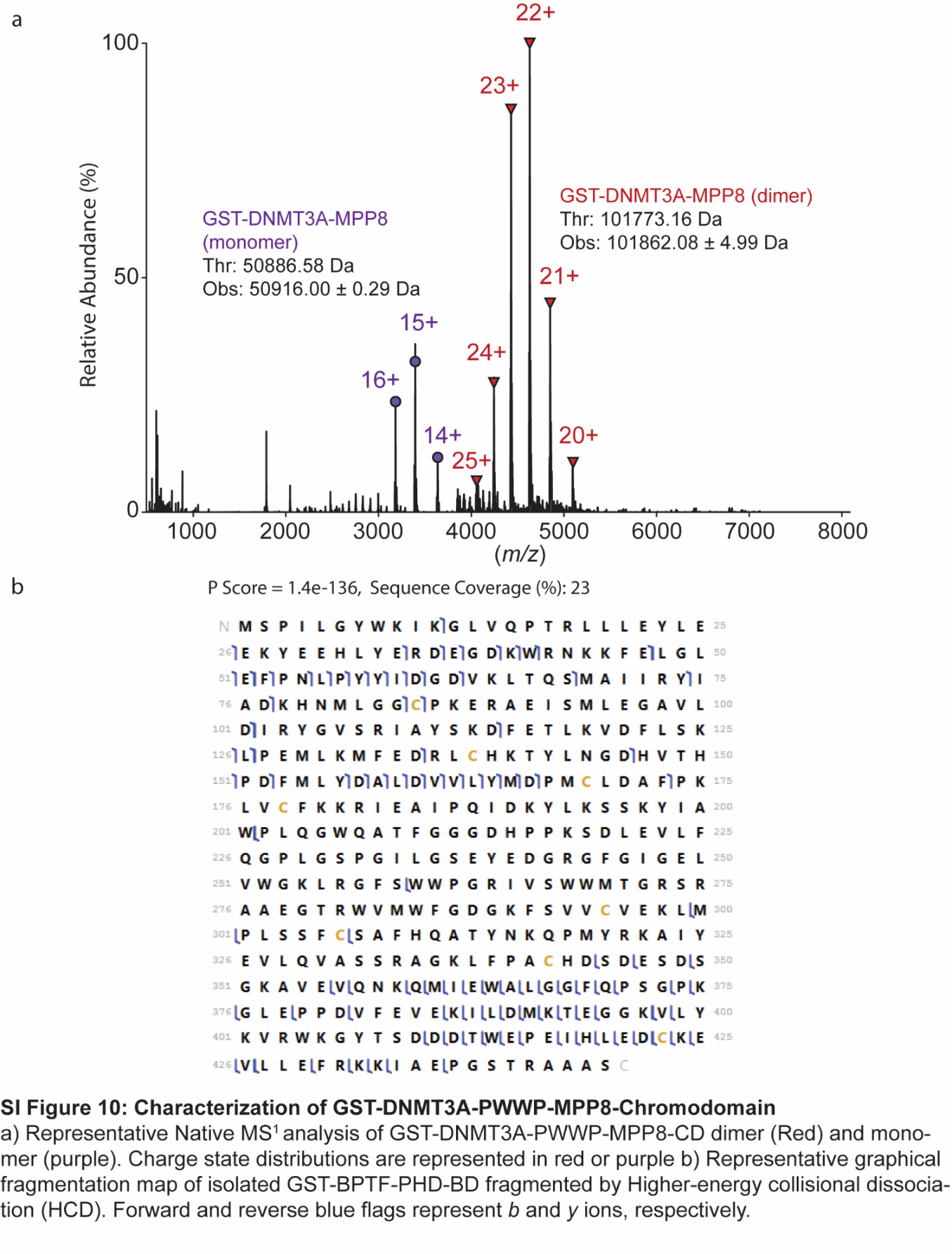


**Figure S14: Characterization of GST-DNMT3A-MPP8 PWWP-CD chimeric tandem reader. a)** Representative Native MS1 analysis of GST-DNMT3A-MPP8 PWWP-Chromodomain chimera tandem reader dimer (possibly via GST-tag) and monomer showing respective charge state distributions in red (+20 to +25) or purple (+14 to +16), respectively. **b)** Representative graphical fragment map of isolated GST-DNMT3A-MPP8 PWWP-CD (precursor ion: 4630.00 m/z) fragmented by higher-energy collisional dissociation (HCD). Forward and reverse blue flags respectively represent b and y ions. Experiments were conducted with three independent biological replicates. Fragments were manually validated using TDValidator and corresponding P-scores calculated using ProSight Lite.


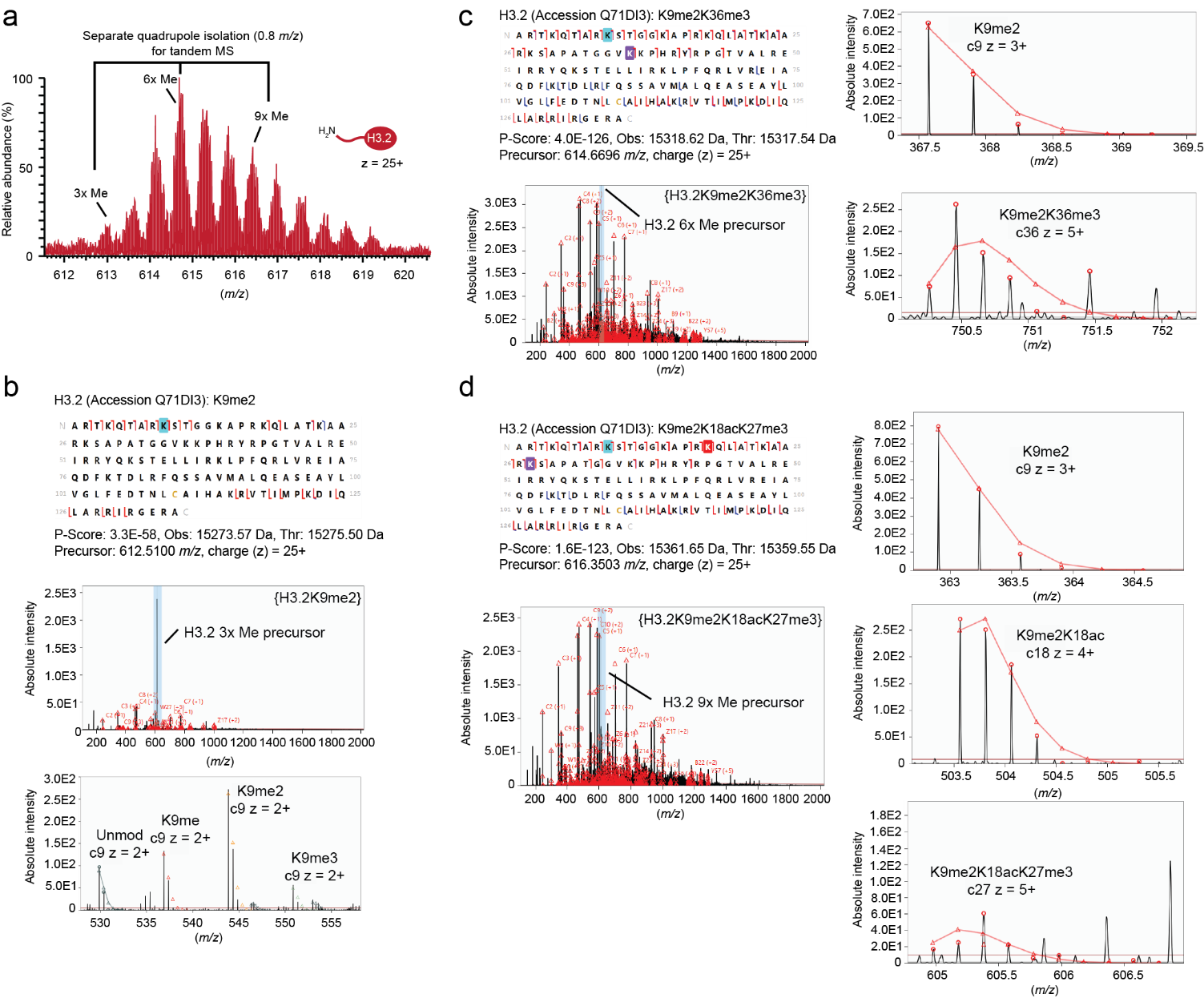


**Figure S15: LC-MS characterization of major H3 proteoforms from DNMT3A-MPP8-enriched endogenous nucleosomes. a)** Representative MS1 spectrum of intact H3.2 proteoform landscape from DNMT3A-MPP8-enriched endogenous nucleosomes by LC-MS. **b)** Representative graphical fragment map of tandem MS fragmentation of {H3.2K9me2}, overall MS2 spectra of {H3.2K9me2} (middle), and representative fragment c ion at K9 isolated from a mixture of H3.2 proteoforms containing +3 methyl equivalents. **c)** Representative graphical fragment map of tandem MS fragmentation of {H3.2K9me2K36me3}, overall MS2 spectra of {H3.2K9me2K36me3}, and representative fragment c ions at K9 and K36 isolated from a mixture of H3.2 proteoforms containing +6 methyl equivalents. **d)** Representative graphical fragment map of tandem MS fragmentation of {H3.2K9me2K18acK27me3}, overall MS2 spectra of {H3.2K9me2K18acK27me3}, and representative fragment c ions at K9, K18, and K27 isolated from a mixture of H3.2 proteoforms containing +9 methyl equivalents. Tandem MS fragmentation was performed using electron-transfer/higher-energy collisional dissociation (EThcD). Forward and reverse red flags respectively represent c and z ions, whereas b and y ions are represented as blue flags. Measurements were performed at three independent biological replicates. Observed (Obs) and theoretical (Thr) are represented as monoisotopic masses (Da). Fragments were manually validated using TDValidator and corresponding P-scores calculated using ProSight Lite.

**
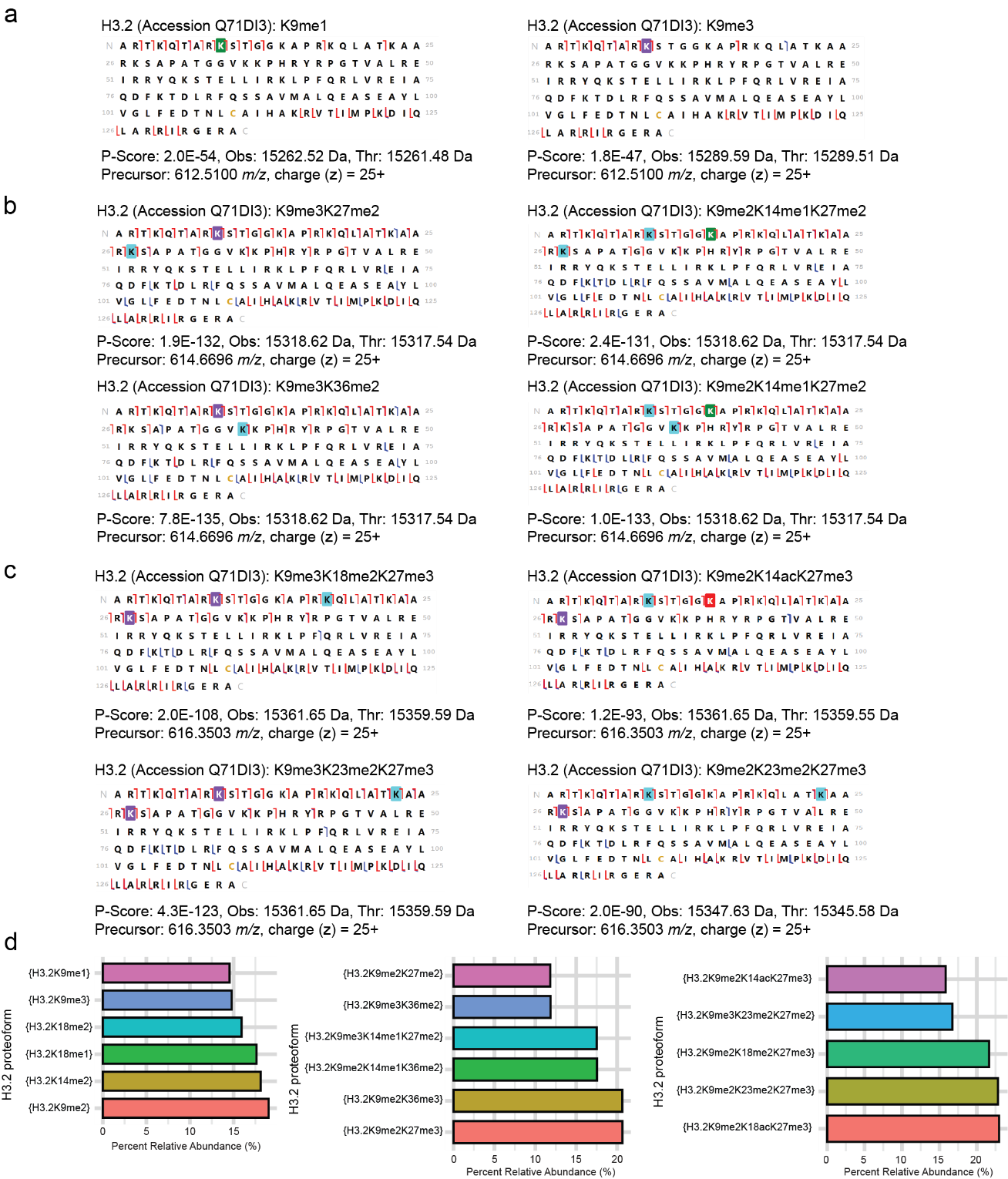
**

**Figure S16: LC-MS characterization of additional H3 proteoforms from DNMT3A-MPP8-enriched endogenous nucleosomes. a)** Representative graphical fragment maps of tandem MS fragmentation of H3.2 proteoforms from isolated from a mixture of H3.2 proteoforms containing +3 methyl equivalents. **b)** Representative graphical fragment maps of tandem MS fragmentation of H3.2 proteoforms from isolated from a mixture of H3.2 proteoforms containing +6 methyl equivalents. **c)** Representative graphical fragment maps of tandem MS fragmentation of H3.2 proteoforms from isolated from a mixture of H3.2 proteoforms containing +9 methyl equivalents. Tandem MS fragmentation was performed using electron-transfer/higher-energy collisional dissociation (EThcD). Forward and reverse red flags respectively represent c and z ions, whereas b and y ions are represented as blue flags. Measurements were performed at three independent biological replicates. Observed (Obs) and theoretical (Thr) are represented as monoisotopic masses (Da). Fragments were manually validated using TDValidator and corresponding P-scores calculated using ProSight Lite. **d)** Percent Relative Abundance of H3.2 proteoforms from H3.2 +3x methyl (left), H3.2 +6x methyl (middle), and H3.2 +9x methyl (right) based on AUC determined by Proteoform Finder.

**
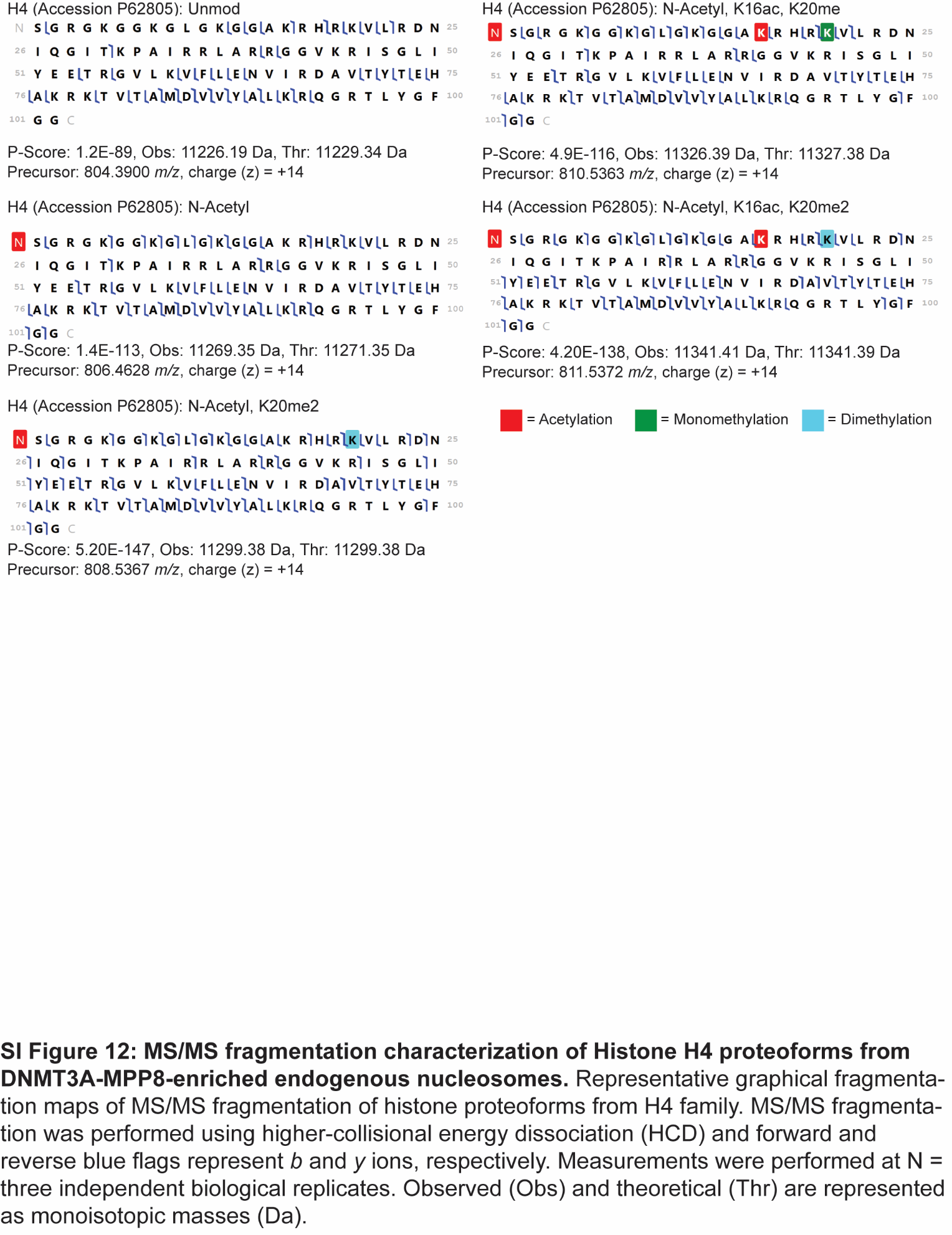
**

**Figure S17: Characterization of H4 proteoforms from DNMT3A-MPP8-enriched endogenous nucleosomes by MS/MS fragmentation.** Representative graphical fragment maps of tandem MS fragmentation of H4 proteoforms in DNMT3A-MPP8 enriched nucleosomes. Tandem MS fragmentation was performed using higher-collisional energy dissociation (HCD) across a distribution of H4 proteoforms (precursor ions (m/z) at charge state (z) +14 represent the intact H4 proteoforms used for tandem MS). Forward and reverse blue flags respectively represent b and y ions. Measurements were performed at three independent biological replicates. Observed (Obs) and theoretical (Thr) are represented as monoisotopic masses (Da). Fragments were manually validated using TDValidator and corresponding P-scores calculated using ProSight Lite.


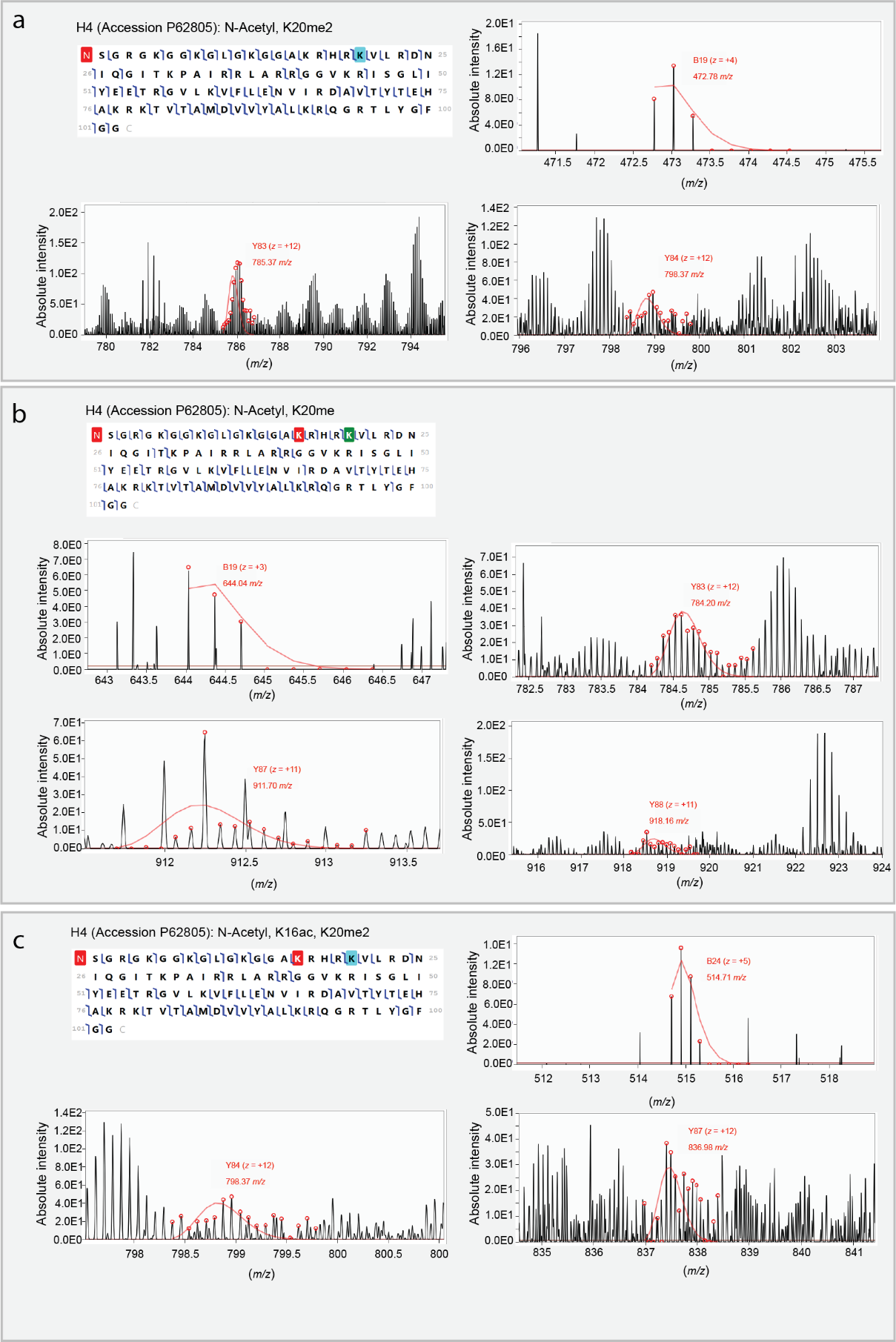


**Figure S18: Tandem MS characterization of H4 proteoforms from DNMT3A-MPP8-enriched endogenous nucleosomes.** Representative tandem MS fragmentation spectra of H4 proteoforms: **a)** {H4K20me2}, **b)** {H4K16acK20me}, and **c)** {H4K16acK20me2} in DNMT3A-MPP8 enriched nucleosomes. Tandem MS fragmentation was performed using higher-collisional energy dissociation (HCD) across a distribution of H4 proteoforms (precursor ions (m/z) at charge state (z) +14 represent the intact H4 proteoforms used for tandem MS). Forward and reverse blue flags respectively represent b and y ions. Measurements were performed with three independent biological replicates. Observed (Obs) and theoretical (Thr) are represented as monoisotopic masses (Da).


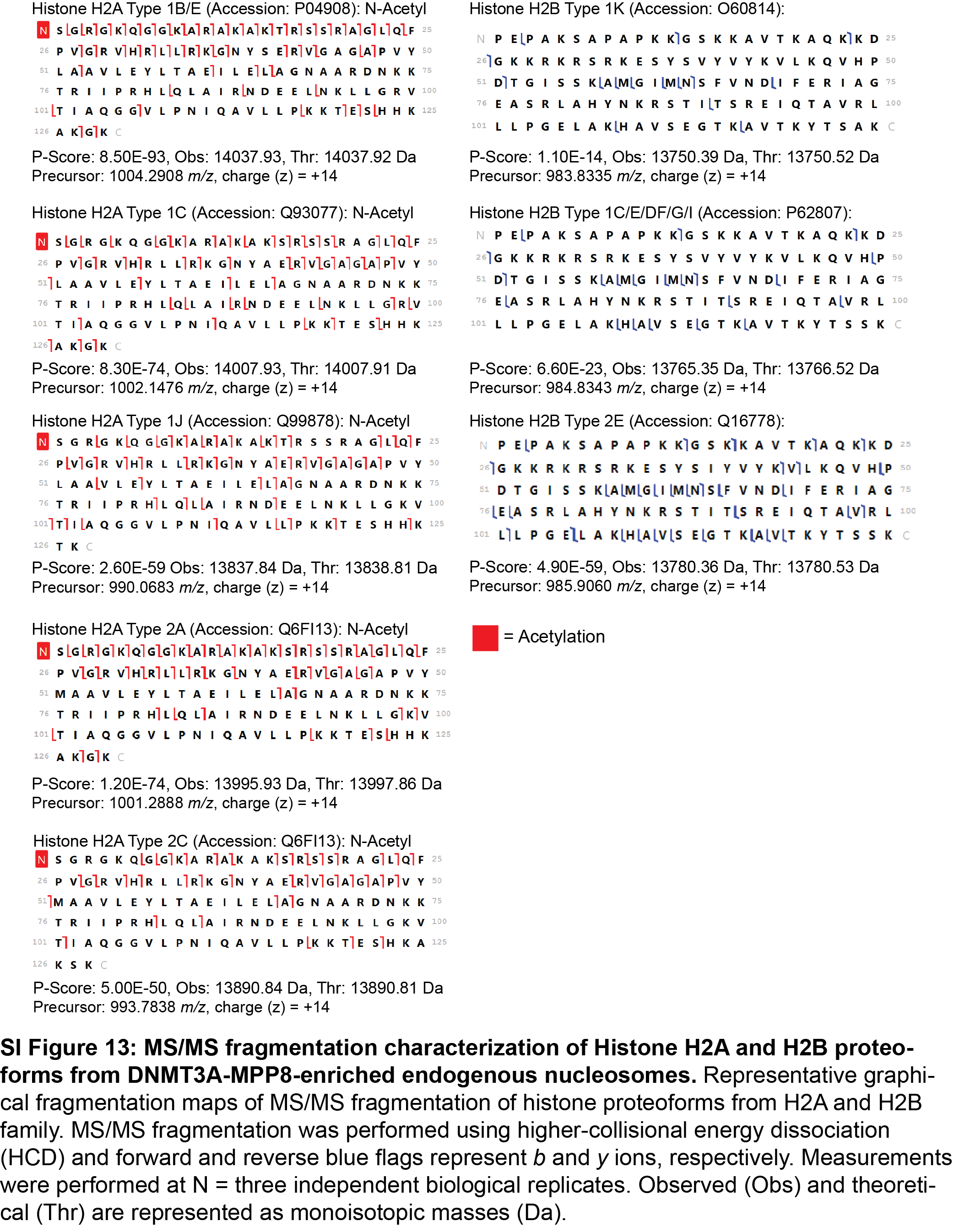


**Figure S19: Characterization of H2A and H2B proteoforms from DNMT3A-MPP8-enriched endogenous nucleosomes.** Representative graphical fragment maps of tandem MS fragmentation of H2A and H2B proteoforms in DNMT3A-MPP8 enriched nucleosomes. Tandem MS fragmentation was performed using Electron-transfer dissociation (ETD) or higher-collisional energy dissociation (HCD) across a distribution of H2A and H2B proteoforms (precursor ions (m/z) at charge state (z) +14 represent the intact H2A and H2B proteoforms used for tandem MS). Forward and reverse red flags respectively represent c and z ions. Forward and reverse blue flags respectively represent b and y ions. Measurements were performed with three independent biological replicates. Observed (Obs) and theoretical (Thr) are represented as monoisotopic masses (Da). Fragments were manually validated using TDValidator and corresponding P-scores calculated using ProSight Lite.


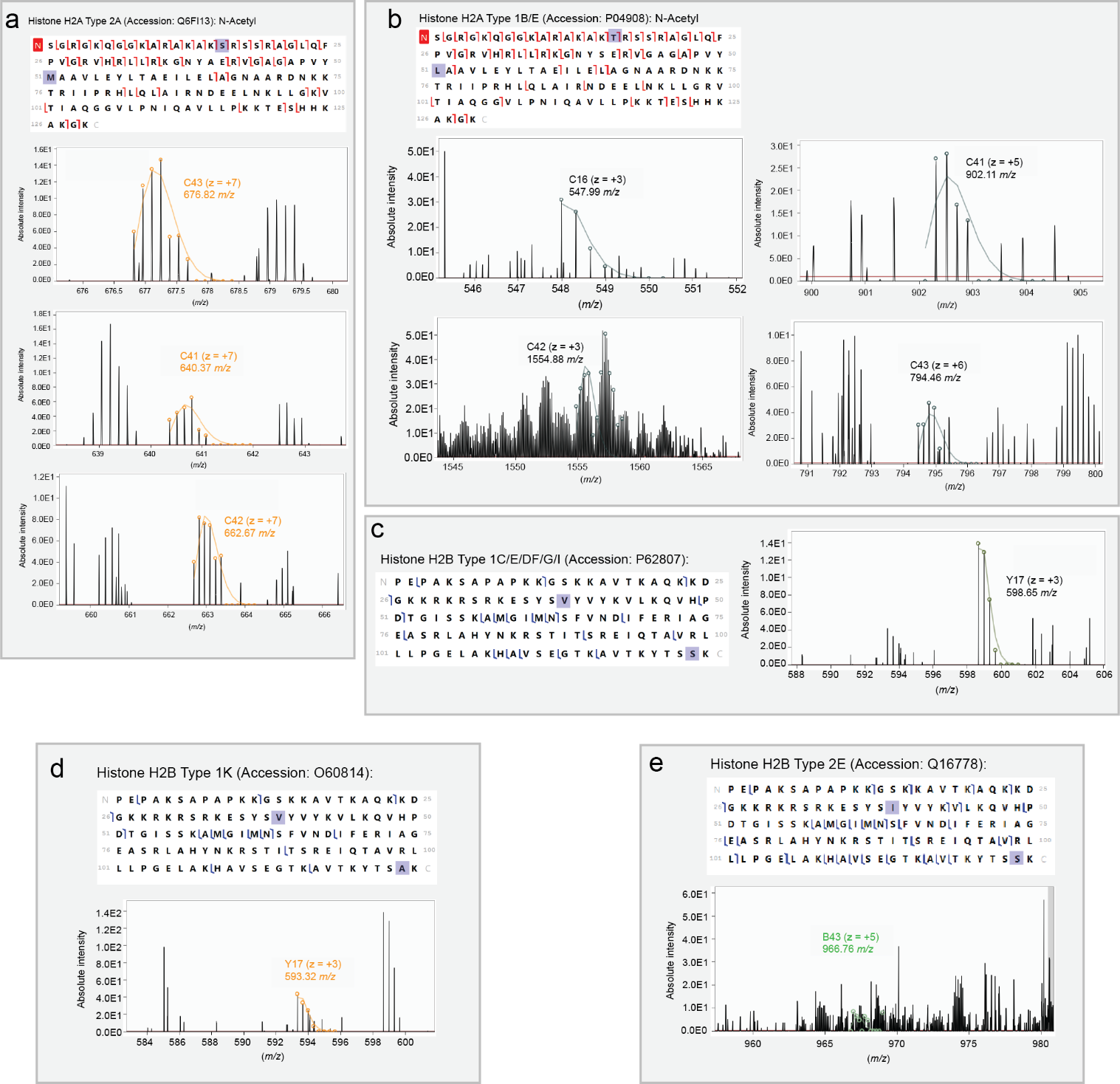


**Figure S20: Tandem MS characterization of H2A and H2B proteoforms from DNMT3A-MPP8-enriched endogenous nucleosomes.** Representative graphical fragment maps and tandem MS fragmentation spectra of: **a)** H2A Type 2A, **b)** H2A Type 1B/E, **c)** H2B Type 1C/E/F/G/I, **d)** H2B Type 1K, and **e)** H2B Type 2E proteoforms in BRD4 enriched nucleosomes. Tandem MS fragmentation was performed using either electron-transfer dissociation (ETD) or higher-collisional energy dissociation (HCD) across a distribution of H2A and H2B proteoforms (precursor ions (m/z) at charge state (z) +14 represent the intact H2A and H2B proteoforms used for tandem MS). Forward and reverse red flags respectively represent *c* and *z* ions*.* Forward and reverse blue flags respectively represent *b* and *y* ions. Measurements were performed with three independent biological replicates. Observed (Obs) and theoretical (Thr) are represented as monoisotopic masses (Da). Purple shade represents single amino acid differences.


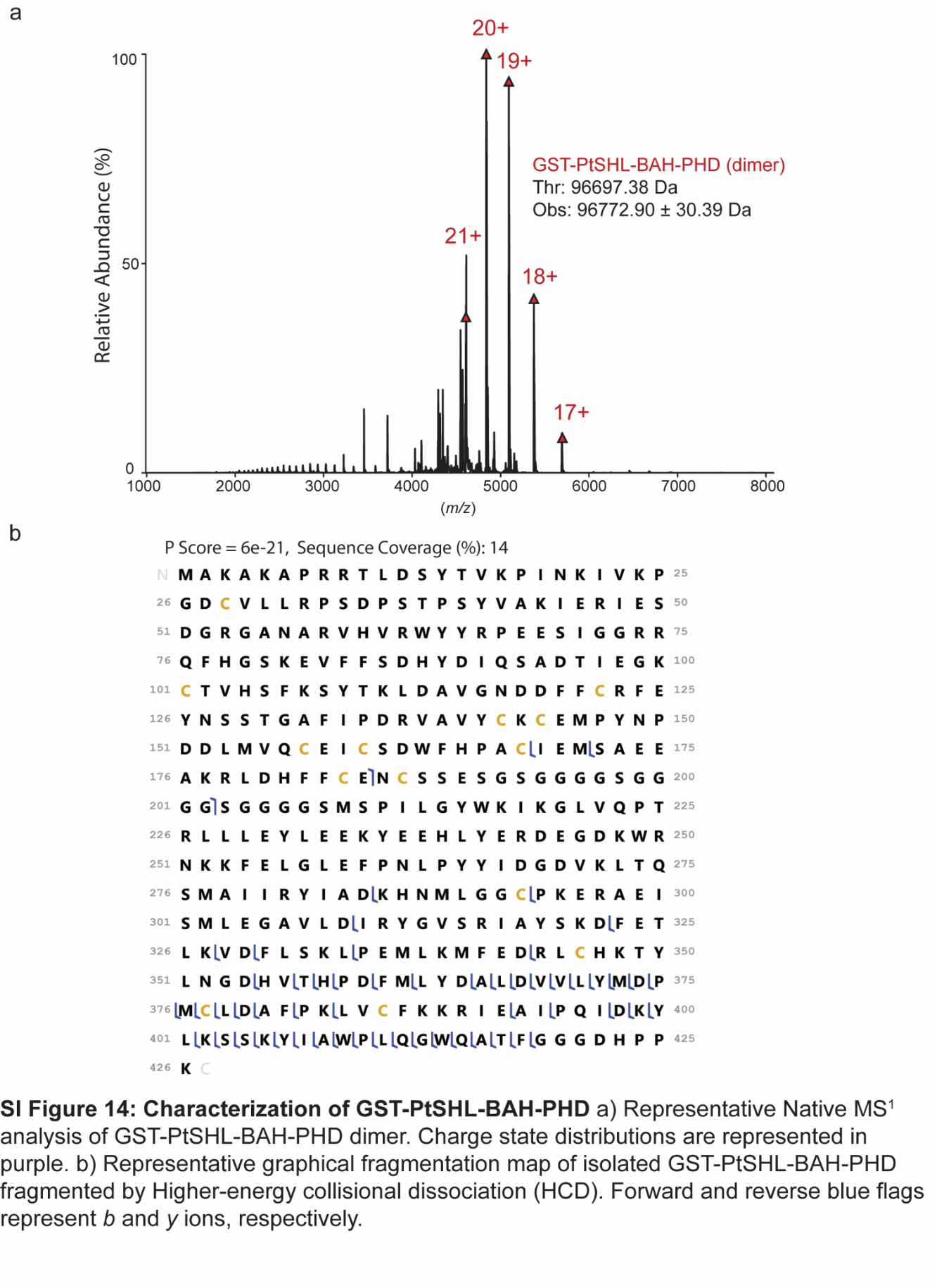


**Figure S21: Characterization of GST-PtSHL BAH-PHD native tandem reader. a)** Representative Native MS1 analysis of GST-PtSHL BAH-PHD dimer (possibly via GST-tag). Charge state distribution of intact GST-PtSHL BAH-PHD dimer is represented in red (+17 to +21). **b)** Representative graphical fragment map of isolated GST-PtSHL BAH-PHD fragmented by higher-energy collisional dissociation (HCD). Forward and reverse blue flags respectively represent b and y ions. Experiments were conducted with three independent biological replicates. Fragments were manually validated using TDValidator and corresponding P-scores calculated using ProSight Lite.

**
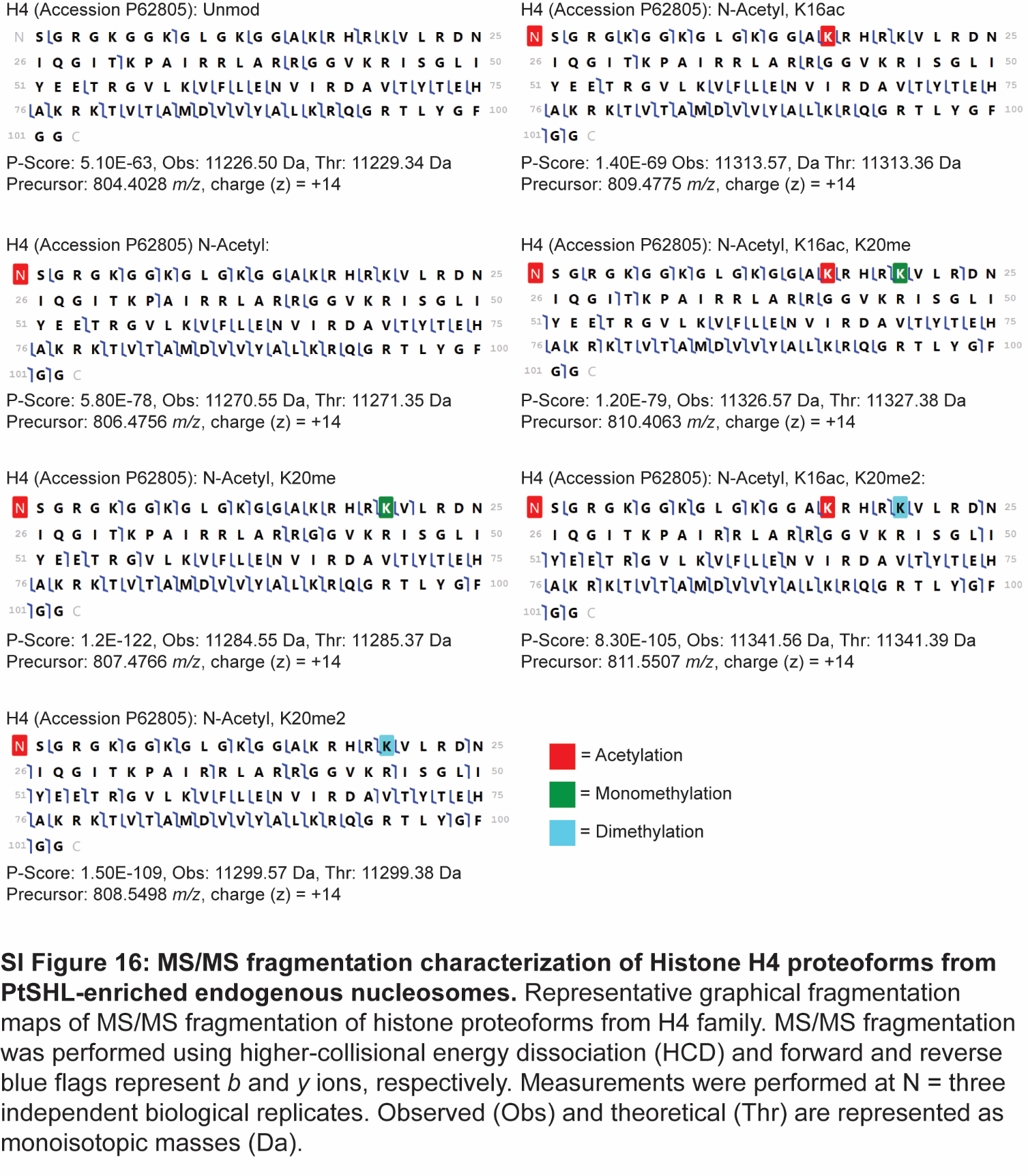
**

**Figure S22: Characterization of H4 proteoforms from PtSHL-enriched endogenous nucleosomes.** Representative graphical fragment maps of tandem MS fragmentation of histone H4 proteoforms in PtSHL-enriched nucleosomes. Tandem MS fragmentation was performed using higher-collisional energy dissociation (HCD) across a distribution of H4 proteoforms (precursor ions (m/z) at charge state (z) +14 represent the intact H4 proteoforms used for tandem MS). Forward and reverse blue flags respectively represent b and y ions. Measurements were performed with three independent biological replicates. Observed (Obs) and theoretical (Thr) are represented as monoisotopic masses (Da). Fragments were manually validated using TDValidator and corresponding P-scores were calculated using ProSight Lite.

*
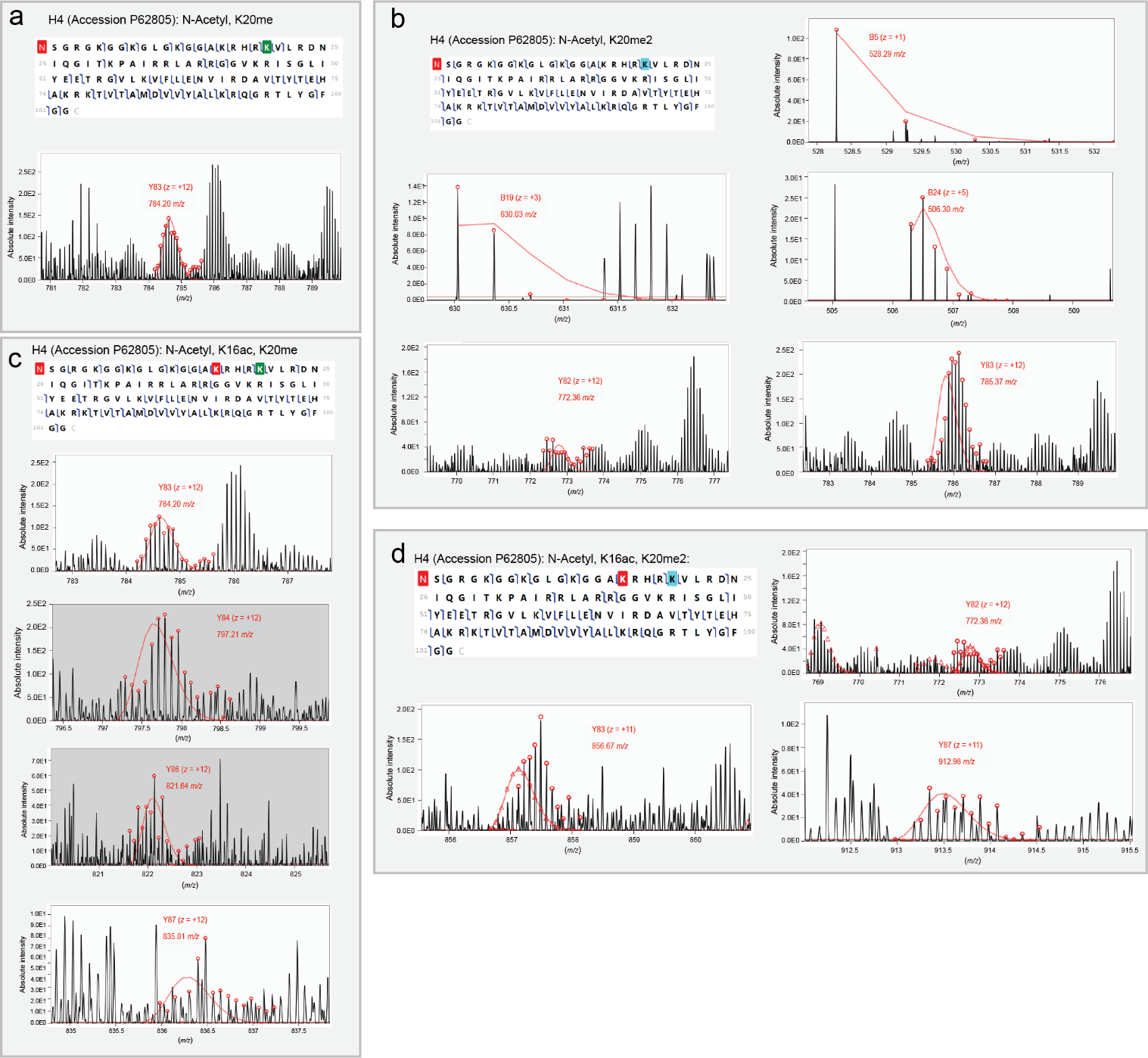
*

**Figure S23: Tandem MS characterization of H4 proteoforms from PtSHL-enriched endogenous nucleosomes.** Representative tandem MS fragmentation spectra of the following H4 proteoforms: **a)** {H4K20me}, **b)** {H4K20me2}, **c)** {H4K16ac20me}, and **d)** {H4K16acK20me2} in PtSHL enriched nucleosomes. Tandem MS fragmentation was performed using higher-collisional energy dissociation (HCD) across a distribution of H4 proteoforms (precursor ions (m/z) at charge state (z) +14 represent the intact H4 proteoforms used for tandem MS). Forward and reverse blue flags respectively represent b and y ions. Measurements were performed with three independent biological replicates. Observed (Obs) and theoretical (Thr) are represented as monoisotopic masses (Da).


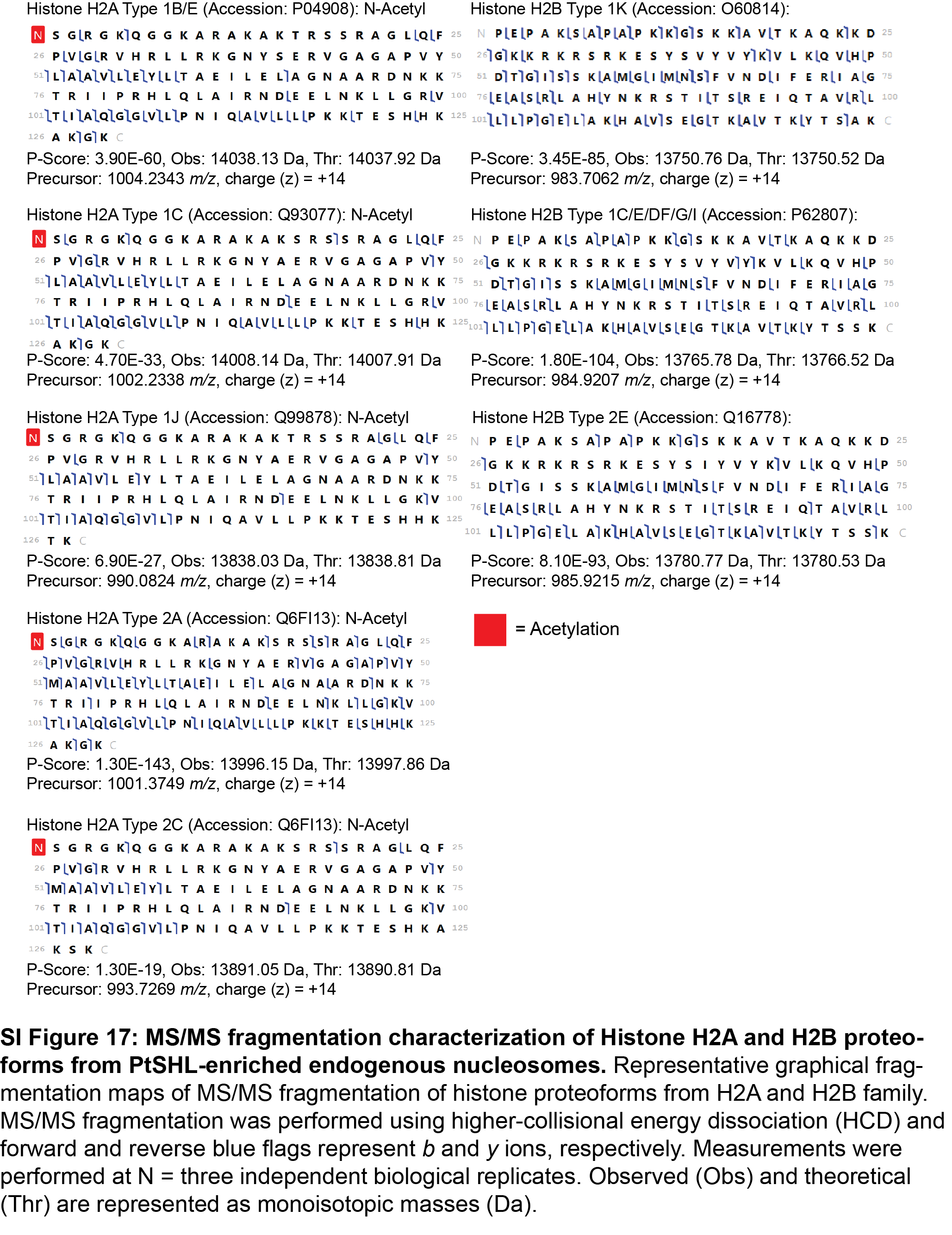


**Figure S24: Characterization of H2A and H2B proteoforms from PtSHL-enriched endogenous nucleosomes.** Representative graphical fragment maps of tandem MS fragmentation of H2A and H2B proteoforms in PtSHL enriched nucleosomes. Tandem MS fragmentation was performed using higher-collisional energy dissociation (HCD) across a distribution of H2A and H2B proteoforms (precursor ions (m/z) at charge state (z) +14 represent the intact H2A and H2B proteoforms used for tandem MS). Forward and reverse blue flags respectively represent b and y ions. Measurements were performed with three independent biological replicates. Observed (Obs) and theoretical (Thr) are represented as monoisotopic masses (Da). Fragments were manually validated using TDValidator and corresponding P-scores calculated using ProSight Lite.


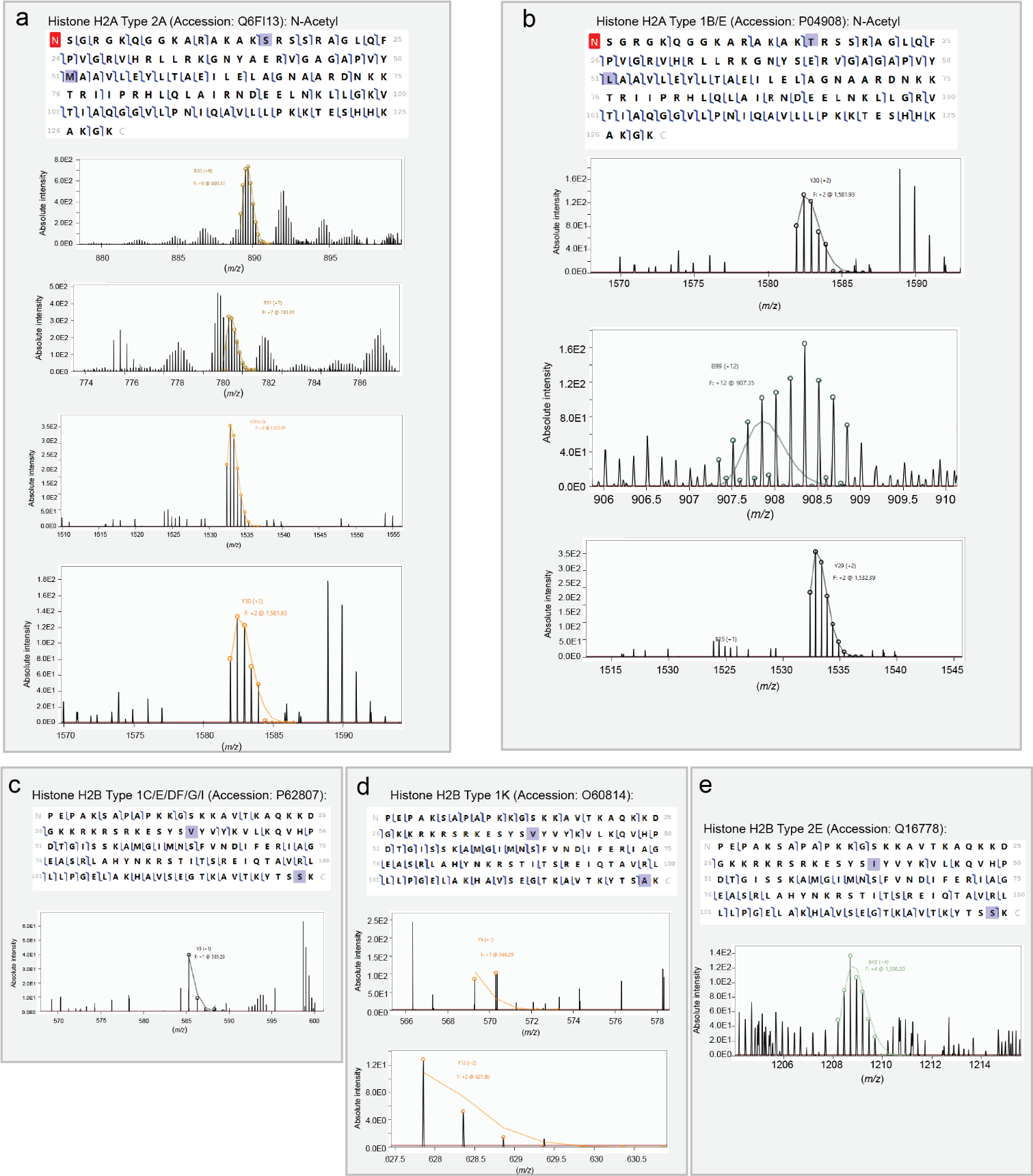


**Figure S25: Tandem MS characterization of H2A and H2B proteoforms from PtSHL-enriched endogenous nucleosomes.** Representative graphical fragment maps and tandem MS fragmentation spectra of **a)** H2A Type 2A, **b)** H2A Type 1B/E, **c)** H2B Type 1C/E/F/G/I, **d)** H2B Type 1K, and **e)** H2B Type 2E proteoforms in BRD4-enriched nucleosomes. Tandem MS fragmentation was performed using higher-collisional energy dissociation (HCD) across a distribution of H2A and H2B proteoforms (precursor ions (m/z) at charge state (z) +14 represent the intact proteoforms used). Forward and reverse red flags respectively represent *c* and *z* ions*.* Forward and reverse blue flags respectively represent *b* and *y* ions. Measurements were performed with three independent biological replicates. Observed (Obs) and theoretical (Thr) are represented as monoisotopic masses (Da). Purple shade represents single amino acid differences.

**
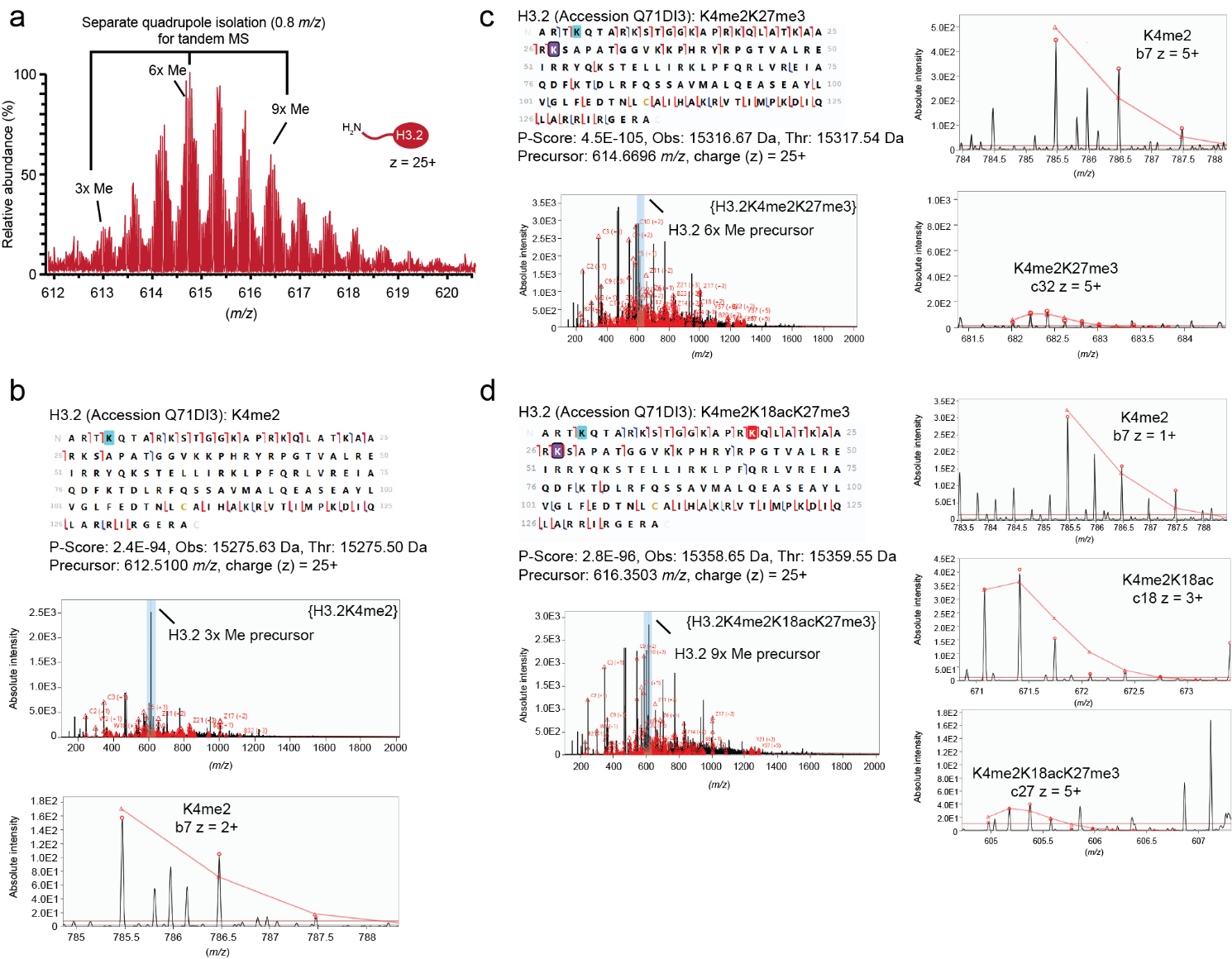
**

**Figure S26: Characterization of major H3 proteoforms from PtSHL-enriched endogenous nucleosomes by LC-MS. a)** Representative MS1 spectrum of intact histone H3.2 proteoform landscape from PtSHL-enriched endogenous nucleosomes by LC-MS. **b)** Representative graphical fragment map of tandem MS fragmentation of {H3.2K4me2}, overall MS2 spectra of {H3.2K4me2} (middle), and representative fragment *c* ion at K4 isolated from a mixture of H3.2 proteoforms containing +3 methyl equivalents. **c)** Representative graphical fragment map of tandem MS fragmentation of {H3.2K4me2K27me3}, overall MS2 spectra of {H3.2K4me2K27me3}, and representative fragment *c* ions at K4 and K27 isolated from a mixture of H3.2 proteoforms containing +6 methyl equivalents. **d)** Representative graphical fragment map of tandem MS fragmentation of {H3.2K4me2K18acK27me3}, overall MS2 spectra of {H3.2K4me2K18acK27me3}, and representative fragment c ions at K4, K18, and K27 isolated from a mixture of H3.2 proteoforms containing +9 methyl equivalents. Tandem MS fragmentation was performed using electron-transfer/higher-energy collisional dissociation (EThcD). Measurements were performed with three independent biological replicates. Observed (Obs) and theoretical (Thr) are represented as monoisotopic masses (Da). Fragments were manually validated using TDValidator and corresponding P-scores calculated using ProSight Lite.

**
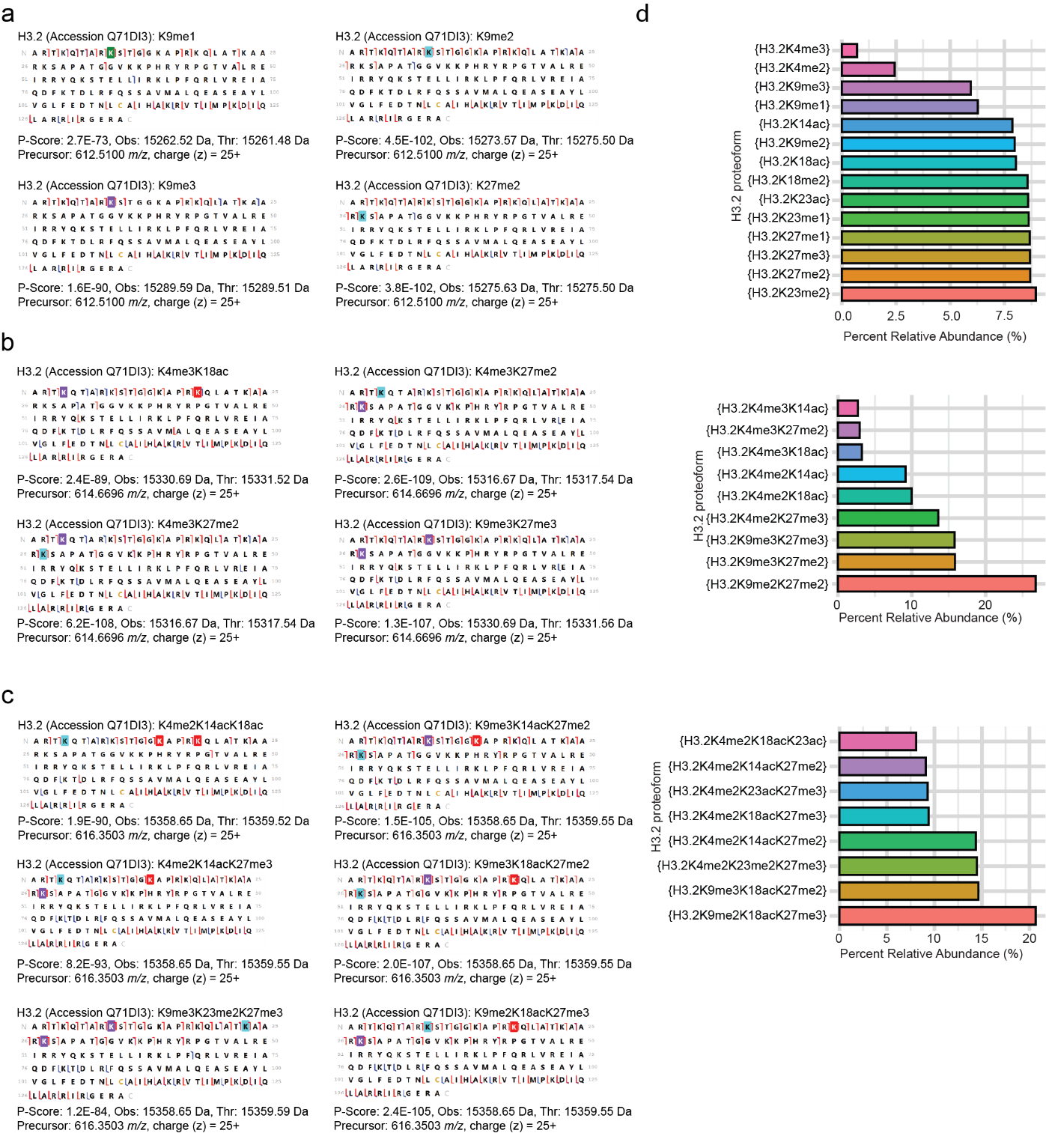
**

**Figure S27: Characterization of additional H3 proteoforms from PtSHL-enriched endogenous nucleosomes by LC-MS. a)** Representative graphical fragment maps of tandem MS fragmentation of H3.2 proteoforms from isolated from a mixture of H3.2 proteoforms containing +3 methyl equivalents. **b)** Representative graphical fragment maps of tandem MS fragmentation of H3.2 proteoforms from isolated from a mixture of H3.2 proteoforms containing +6 methyl equivalents. **c)** Representative graphical fragment maps of tandem MS fragmentation of H3.2 proteoforms from isolated from a mixture of H3.2 proteoforms containing +9 methyl equivalents. Tandem MS fragmentation was performed using electron-transfer/higher-energy collisional dissociation (EThcD). Forward and reverse red flags respectively represent c and z ions, whereas b and y ions are represented as blue flags. Measurements were performed at three independent biological replicates. Observed (Obs) and theoretical (Thr) are represented as monoisotopic masses (Da). Fragments were manually validated using TDValidator and corresponding P-scores calculated using ProSight Lite. **d)** Percent Relative Abundance of H3.2 proteoforms from H3.2 +3x methyl (top), H3.2 +6x methyl (middle), and H3.2 +9x methyl (bottom) based on AUC determined by Proteoform Finder.

**Table S1:** Relative Effective Concentration (EC_50_^Rel^) in Luminex assays to measure the interaction between GST-BPTF PHD-BD constructs and PTM-defined histone H3 peptides.

| Construct | H3_[1-20]_ | | | H3_[1-20]_K4me3 | | | H3_[1-20]_K4acK9ac, K14acK18ac | | | H3_[1-20]_K4me3 K9acK14acK18ac | | |
| --- | --- | --- | --- | --- | --- | --- | --- | --- | --- | --- | --- | --- |
|  | 1 | 2 | 3 | 1 | 2 | 3 | 1 | 2 | 3 | 1 | 2 | 3 |
| GST-BPTF PHD-BD | 75 | 56 | 83 | 16.6 | 16.6 | 23.4 | 60.1 | 56.4 | 76.7 | 3.2 | 3.3 | 6.4 |
| GST-BPTF PHD*-BD | 267 | 177 | 271 | 419 | 313 | 333 | 533 | 397 | 491 | 238 | 161 | 235 |
| GST-BPTF PHD-BD* | 169 | 133 | 138 | 18.8 | 29.5 | 16.6 | 553 | 508 | 307 | 8.1 | 13.7 | 9 |
| GST-BPTF PHD*-BD* | 118 | 88 | 116 | 442 | 391 | 262 | 639 | 554 | 311 | 416 | 390 | 276 |

**Table S2:** Averaged (AVG) and Standard Deviation (ST.DEV) of EC_50_^Rel^ in Luminex assays to measure the interaction between GST-BPTF-PHD-BD constructs and PTM-defined histone H3 peptides.

| Construct | H3_[1-20]_ | | H3_[1-20]_K4me3 | | H3_[1-20]_K4acK9ac, K14acK18ac | | H3_[1-20]_K4me3K9ac, K14acK18ac | |
| --- | --- | --- | --- | --- | --- | --- | --- | --- |
|  | AVG | ST.DEV | AVG | ST.DEV | AVG | ST.DEV | AVG | ST.DEV |
| GST-BPTF-PHD-BD | 71.3 | 13.9 | 18.9 | 3.9 | 64.4 | 10.8 | 4.3 | 1.8 |
| GST-BPTF-PHD*-BD | 238.3 | 53.2 | 355.0 | 56.3 | 473.7 | 69.6 | 211.3 | 43.6 |
| GST-BPTF-PHD-BD* | 146.7 | 19.5 | 21.6 | 6.9 | 456.0 | 131.0 | 10.3 | 3.0 |
| GST-BPTF PHD*-BD* | 107.3 | 16.8 | 365.0 | 92.8 | 501.3 | 170.2 | 360.7 | 74.5 |

**Table S3:** EC_50_^Rel^ from Luminex assay between GST-tagged BPTF constructs and PTM-defined semi-synthetic nucleosomes.

| Construct | ([unmodified]) | | | ([H3K4me3]_2_) | | | ([H3K4acK9ac K14acK18ac]_2_) | | | ([H3K4me3K9ac K14acK18ac]_2_) | | |
| --- | --- | --- | --- | --- | --- | --- | --- | --- | --- | --- | --- | --- |
|  | 1 | 2 | 3 | 1 | 2 | 3 | 1 | 2 | 3 | 1 | 2 | 3 |
| GST-BPTF-PHD-BD | >1000 | 993 | >1000 | 128 | 85 | 123 | 324 | 240 | 378 | 25 | 16.4 | 28.7 |
| GST-BPTF-PHD*-BD | 762 | 671 | 738 | >1000 | >1000 | >1000 | >1000 | >1000 | >1000 | 340 | 300 | 382 |
| GST-BPTF-PHD-BD* | >1000 | >1000 | 776 | 356 | 509 | 234 | >1000 | >1000 | >1000 | 51 | 73 | 62 |
| GST-BPTF PHD*-BD* | 225 | 192 | 212 | 587 | 481 | 309 | 778 | 646 | 428 | 202 | 167 | 177 |

**Table S4:** Averaged (AVG) and Standard Deviation (ST.DEV) of EC_50_^Rel^ from Luminex assay between GST-tagged BPTF constructs and PTM-defined semi-synthetic nucleosomes.

| Construct | ([unmodified]) | | ([H3K4me3]_2_) | | ([H3K4acK9ac K14acK18ac]_2_) | | ([H3K4me3K9ac K14acK18ac]_2_) | |
| --- | --- | --- | --- | --- | --- | --- | --- | --- |
|  | AVG | ST.DEV | AVG | ST.DEV | AVG | ST.DEV | AVG | ST.DEV |
| GST-BPTF-PHD-BD | >1000 | ND | 112.0 | 23.5 | 314.0 | 69.5 | 23.4 | 6.3 |
| GST-BPTF-PHD*-BD | 723.7 | 47.2 | >1000 | ND | >1000 | ND | 340.7 | 41.0 |
| GST-BPTF-PHD-BD* | >1000 | ND | 366.3 | 137.8 | >1000 | ND | 62.0 | 11.0 |
| GST-BPTF PHD*-BD* | 209.7 | 16.6 | 459.0 | 140.3 | 617.3 | 176.8 | 182.0 | 18.0 |

**Nuc-MS parameters**

- The Orbitrap Q Exactive Ultra High Mass Range (UHMR) and Orbitrap Ascend Tribrid MS are capable of scan ranges upwards to 8000 *m/z*. These MS instruments are most suitable for Nuc-MS analyses of readers and nucleosomes.
- The Orbitrap Ascend or Eclipse Tribrid MS was used specifically for the characterization of nucleosome-CAP complexes and their proteoforms due to multi-modal fragmentation approaches including Higher Collisional Dissociation (HCD), and Electron-Transfer Dissociation (ETD) allowing for increase in sequence coverage and PTM localization. **See Tables S5-S9.**

**Table S5:** Settings used for intact nMS analysis of BPTF:nucleosome complexes (MS1).

| **MS instrument** | Orbitrap Q Exactive UHMR MS |
| --- | --- |
| **Ion source type** | NSI |
| **Positive ion spray voltage (V)** | 1800-2500 |
| **Ion transfer tube temp (°C)** | 310 |
| **Pressure setting** | High Pressure Mode |
| **Scan type** | MS |
| **Detector type** | Orbitrap |
| **Orbitrap resolution at 400 *m/z*** | 6250 |
| **Mass range** | High |
| **Scan range (*m/z*)** | 500-15000 |
| **Microscan** | 2-20 |
| **RF lens (%)** | 150 |
| **AGC target** | 1x10^6^ |
| **Maximum injection time (ms)** | 20-175 |
| **Source fragmentation (V)** | 0 |
| **In-source trapping (IST) desolvation (V)** | -100 |
| **In-source trapping time (ms)** | 4 |

**Table S6:** Settings used for intact nMS analysis of CAPs (MS1).

| **MS instrument** | Orbitrap Ascend Tribrid MS |
| --- | --- |
| **Application mode** | Intact Protein |
| **Ion source type** | NSI |
| **Positive ion spray voltage (V)** | 1400-1800 |
| **Ion transfer tube temp (°C)** | 320 |
| **Pressure setting** | High Pressure Mode |
| **Scan type** | MS |
| **Detector type** | Orbitrap |
| **Orbitrap resolution** | 7500 |
| **Mass range** | High |
| **Scan range (*m/z*)** | 500-8000 |
| **Microscan** | 2 |
| **RF lens (%)** | 150 |
| **Normalized AGC target (%)** | 150 |
| **Maximum injection time (ms)** | 300 |
| **Source fragmentation (V)** | 40-80 |

**Table S7:** Settings used for MS/MS fragmentation analysis of CAPs (MS2).

| **MS instrument** | Orbitrap Ascend Tribrid MS |
| --- | --- |
| **Application mode** | Intact Protein |
| **Ion source type** | NSI |
| **Positive ion spray voltage (V)** | 1400-1800 |
| **Ion transfer tube temp (°C)** | 320 |
| **Pressure setting** | High Pressure Mode |
| **Scan type** | MS^2^ |
| **Isolation width (m/z)** | BRD4: 500 *m/z*, DNMT3A-MPP8: 1000 *m/z*, PtSHL: 50 *m/z* |
| **Activation type** | HCD |
| **Collision energy type** | Normalized |
| **HCD collision energy (%)** | 20-55 |
| **Detector** | Orbitrap |
| **Orbitrap resolution** | 120000 |
| **Mass range** | High |
| **Scan range (*m/z*)** | 500-8000 |
| **RF lens (%)** | 150 |
| **Normalized AGC target (%)** | 1000 |
| **Maximum injection time (ms)** | 1000 |
| **Microscans** | 2 |
| **Source fragmentation (V)** | 40-80 |

**Table S8:** Settings used for characterization of ejected intact histone proteoforms from CAP-nucleosome complexes (MS1).

| **MS instrument** | Orbitrap Ascend Tribrid MS |
| --- | --- |
| **Application mode** | Intact Protein |
| **Ion source type** | NSI |
| **Positive ion spray voltage (V)** | 1400-1800 |
| **Ion transfer tube temp (°C)** | 320 |
| **Pressure setting** | Low Pressure Mode |
| **Scan type** | MS |
| **Detector type** | Orbitrap |
| **Orbitrap resolution** | 60000-120000 |
| **Mass range** | High |
| **Scan range (*m/z*)** | 500-8000 |
| **Microscan** | 1-2 |
| **RF lens (%)** | 150 |
| **Normalized AGC target (%)** | 150 |
| **Maximum injection time (ms)** | 500 |
| **Source fragmentation (V)** | 250 |

| **MS instrument** | Orbitrap Ascend Tribrid MS |
| --- | --- |
| **Application mode** | Intact Protein |
| **Ion source type** | NSI |
| **Positive ion spray voltage (V)** | 1400-1800 |
| **Ion transfer tube temp (°C)** | 320 |
| **Pressure setting** | Low Pressure Mode |
| **Scan type** | MS^2^ |
| **Isolation width (*m/z*)** | H2A: 20-30 *m/z*, H2B: 20-30 *m/z*, H3.2: 3.3 *m/z*, H4: 20-30 *m/z*  For BRD4 H4: 3 m/z |
| **Activation type** | HCD |
| **Collision energy type** | Normalized |
| **HCD collision energy (%)** | 30-60 |
| **Activation type: For BRD4 H4** | ETD |
| **ETD reaction time (ms)** | 4 |
| **ETD reagent target** | 2.5e5 |
| **Max ETD reagent injection time (ms)** | 200 |
| **Detector** | Orbitrap |
| **Orbitrap resolution** | 120000 |
| **Mass range** | High |
| **Scan range (m/z)** | 500-8000 |
| **RF lens (%)** | 150 |
| **Normalized AGC target (%)** | 1000 |
| **Maximum injection time (ms)** | 1000 |
| **Microscans** | 1-2 |
| **Source fragmentation (V)** | 250 |

**Table S9:** Settings used for MS/MS fragmentation analysis of histone proteoforms (MS2).

**Table S10:** Targeted Mass List for Parallel Reaction Monitoring of H3.2 proteoforms for denatured Top-Down LC-MS.

| **Precursor** | ***m/z*** | ***z*** | **t start (min)** | **t start (max)** | **ETD reaction time (ms)** | **SA Collision Energy (%)** |
| --- | --- | --- | --- | --- | --- | --- |
| H3.2 3x Methyl | 612.5100 | 25 | 36 | 44 | 14 | 35 |
| H3.2 6x Methyl | 614.6696 | 25 | 36 | 44 | 14 | 35 |
| H3.2 9x Methyl | 616.3503 | 25 | 36 | 44 | 14 | 35 |

**Table S11:** EC_50_^Rel^ for PtSHL binding to defined homotypic and heterotypic nucleosomes.

| Nucleosomes (**Homotypic** and *Heterotypic*) | EC_50_^Rel^, nM |
| --- | --- |
| **([H3.1]_2_)** | ND |
| **([H3K4me3]_2_)** | 138 |
| **([H3K9me3]_2_)** | 147 |
| **([H3K27me3]_2_)** | 26.0 |
| **([H3K4me3K9me3]_2_)** | 68.2 |
| **([H3K4me3K27me3]_2_)** | 0.17 |
|  |  |
| *([H3]•[H3K4me3])* | 149 |
| *([H3]•[H3K27me3])* | 77.1 |
| *([H3]•[H3K4me3K27me3])* | 0.12 |
| *([H3K4me3]•[H3K27me3])* | 43.0 |
