# Supplementary material for: Direct Readout of Multivalent Chromatin Reader-Nucleosome Interactions by Nucleosome Mass Spectrometry
Source: ACS Cent Sci. 2026 Feb 5;12(2):208–21. doi: 10.1021/acscentsci.5c00736 (PMC12947556; doi:10.1021/acscentsci.5c00736)
Supplement: Supplementary file 2 [file oc5c00736_si_002.pdf]

oc-2025-00736j.R1

Name: Peer Review Information for "Direct Readout of Multivalent Chromatin Reader-Nucleosome Interactions by Nucleosome Mass Spectrometry"

First Round of Reviewer Comments

Reviewer: 1

Comments to the Author

This is an interesting paper that shows the use of Nuc:MS to directly characterize CAP:nuc complex composition, with combinatorial PTM information, in a single readout, with Nuc:MS allowing discovery of the modifications driving binding, and therefore primary candidates to explore for structural biology and genomic studies.

There are a few changes needed before this manuscript can be published:

1) make clearer the distinctions between this study and the previous 2021 Nat Meth work and the importance of knowing the modifications/combinatorial PTM information; state these clearly early in the manuscript and repeat them in the conclusions

2) use fewer acronyms in the abstract and define those that are used; the authors are so used to this field that they may not notice how difficult the acronyms are for non-experts but to publish in a broader journal, this needs to be corrected

3) explain/define the percentages mentioned e.g., when stating 75% enrichment

4) consider whether the charge states of the products have meaning (maybe only related to extent of unfolding)

Reviewer: 2

Comments to the Author

The manuscript by Lee et al. presents an elegant and technically rigorous study discussing capabilities of Nuc-MS, a top-down mass spectrometry approach introduced in a prior publication. By applying Nuc-MS to both synthetic and endogenous nucleosomes, the authors demonstrate that multivalent chromatin reader domains (e.g., BPTF, BRD4, DNMT3A–MPP8, and SHL) recognize distinct, combinatorial histone PTM patterns. These findings significantly advance our ability to characterize the "histone code" in native chromatin contexts. The work is of high impact and should be of broad interest to the chromatin, epigenetics, and mass spectrometry communities. I believe this manuscript is suitable for publication, pending minor revisions. Specific comments are below:

1) The Nuc-MS approach is a major technical advance, but the manuscript could briefly address practical considerations such as capture efficiency, sensitivity, and current limitations (e.g., need for high-end instrumentation, potential detection bias against low-abundance proteoforms).

2) For BPTF, the necessity of both PHD and bromodomain is clearly shown. However, for other readers (BRD4, DNMT3A–MPP8, SHL), it is assumed that both domains are required. If available, inclusion of single-domain controls (or discussion thereof) would strengthen the claim that multivalency drives specificity.

3) The top-down MS/MS spectra are impressive and well-supported. However, we know that top-down struggles in mapping with high confidence modifications when isobaric forms are present (and histones have a lot!). Is it possible to understand how much of a risk is the presence of endogenous HeLa histones? It is a bit confusing to me how much in the sample is the CAP:Nuc and how much is the endogenous. The text claims that they are mixed. Maybe I did not understand.

4) The work is analytically focused, but some contextualization of the proteoform combinations (e.g., H3K9me3 + K36me3 for DNMT3A–MPP8, or H3K4me3 + K27me3 + K79me2 for SHL) would strengthen the biological relevance. Prior reports of such bivalent marks or chromatin states could be cited to guide interpretation.

5) The observation of a 2:1 BPTF:nucleosome ratio is intriguing. Could this be due to GST dimerization or independent binding on each H3 tail? If untagged or cleaved protein was tested, please clarify. Otherwise, acknowledging this uncertainty would be sufficient.

6) Please, make the raw data available in MassIVE. I could not check the files.

Reviewer: 3

Comments to the Author

In this paper the authors use the recently developed capability of analyzing intact nucleosomes beyond the context than their previous work that demonstrated proof of principle. They primarily focus on the binding of multivalent readers amongst the chromatin associated proteins. BRD4 is a classic well studied and important acetyl lysine reader with two bromodomains. DNMT3A-MPP8 is an interesting fusion protein that also contains multiple reader domains. PtSHL is another multi reader domain CAP.

It is not made clear the difference between different bracketing schemes. It also at times inconsistent with the attached supplemental paper.

"…requires coincident H3K4me3K9acK14acK18ac for effective nucleosome engagement." This statement seems a little too specific. Perhaps adding a qualifier "of the configurations tested?" I believe this is one of the proteoforms but there are undoubtedly others that are similarly preferred or even more.

It should be more clearly stated that K44ac probably would not have been considered for investigation using other approaches. Also, show the spectra in supplemental that localizes this PTM. There are ways to be wrong about this. Others have noted various adducts (sulfate adducts that survive ETD reactions) and oxidations in this region.

Define "methyl equivalents" on first use. Also, this should be brought into the discussion about the limitations of the methods. Sometimes the authors are not truly discriminating proteoforms but more proteoform families based on shared precursor mass, which isn't particularly biologically meaningful.

H4K16acK20me2 is quite common. Please consider/discuss this when addressing H4K16acK20me2.

In SI Fig 7 why are so many N-terminal fragments missing? These should be the most prominent. While there are sufficient y ions for PTM localization it would stronger with both b- and y- ions to confirm proteoform ID. In SI fig 13 these near the N-terminus b- ions are present.

SI Fig 16

The majority of the strongest peaks are not assigned. Without any chromatographic separation of the endogenous proteoform I would expect these spectra would be near uninterpretable since many possible PTM localizations for each precursor mass (structural isomers). Of course it could be that the CAP binding is highly selective and only one (or a few) proteoforms may be present. This figure and the absence of other figures does not show well that the mixture of proteoforms is simplified.

I would have liked to see a few more examples of annotated MS3 spectra showing localization of PTMs to define proteoforms. I expect that there would be some contradictory evidence and perhaps some mixture of proteoforms present in the spectra. Certainly this would not imply misinterpretation but I would like to see for myself in supplemental information typical spectra and how mixed these spectra are. It should also

be discussed in text, again claiming that the spectra is primarily one proteoform is completely valid even when revealing that it is not pure.

The number of H2B Sequences seems unusually limited compared to prior work from the Mag alb and others that addressed that the precursor masses are often isobaric and thus generate chimeric spectra. Is this because you are not distinguishing some variants and they are being grouped with others (but not delayed as such) or are you limited in dynamic range? Could you provide tandem MS that makes it clear that you are successfully discriminating between the known sequences or otherwise clearly state any ambiguity?

This statement need much more support and perspective: “Of note the latter enriches combinatorial {H3K4me3K27me3} on the same histone tail in HeLa chromatin, and thus expands the potential biology of this widely studied bivalent signature.” While this may be considered a small point about yet another combination of piston PTMs, its very existence in any human or mammal cells has been an issue of debate for more than decade. You would do the world a service by presenting the definitive evidence in a clear and easily understood form. You also need to put this in perspective. I do not think this result invalidates the general truth that K4me3-K27me3 ChIP-seq tracks are almost exclusively in trans. What is the relative abundance of this combination? What other marks are they with? It has been shown over and over that K4me3 prefers hyper acetylation. Is this not true for K4me3-K27me3. A paper could and possibly should be written on this one result. I would consider putting the spectral evidence for this form in a main figure.

Overall, while this work is important, well written, and methodologically solid, the manuscript in its current form is unsuitable for publication. It is lacking primarily in the clear presentation of evidence that support the author’s claims without requiring substantial work of the reader who will often not have the tools or the knowledge to retrieve the data and do their own analysis. It is also lacking in realistic perspective that clearly represents the limitations of this exciting new methodology and the proper implications of their findings such that others will not over simplify or over estimate. Think of the ways that a less informed reader might over reach in their assumptions. “K4me3-K27me3 is in cis not trans.” “There are only a few H2B sequences in HeLA cells.” These readers only enrich one specific proteoform and has little affinity for others. The method produces clean unmixed tandem mass spectra.

Author's Response to Peer Review Comments:

Walter and Mary Elizabeth Glass Professor in the Life Sciences
Director, Proteomics Center of Excellence
Director, Chemistry of Life Processes Institute
Professor in the Departments of Chemistry and Molecular Biosciences and the Feinberg School of Medicine

Office Address
3613 Silverman Hall
2170 Campus Drive
Evanston, IL 60208

Mailing Address
2-100 Hogan Hall
2205 Tech Drive
Evanston, IL 60208

Phone: 847-467-4362
Fax: 847-467-3276
Email: n-kelleher@northwestern.edu
Research group website: www.kelleher.northwestern.edu
Center website: http://pce.northwestern.edu

August 1st, 2025

Executive Editor
American Chemical Science Central Science

Manuscript: Direct Readout of Multivalent Chromatin Reader-Nucleosome Interactions by Nucleosome Mass Spectrometry

Authors: Alexander S. Lee, Nickolas P. Fisher, Matthew R. Marunde, Pei Su, Laiba F. Khan, Bria Graham, Hailey F. Taylor, Ugochi C. Onuoha, Taojunfeng Su, Kevin Jooß, Luis F. Schachner, Harrison A. Fuchs, Kelsey Noll, Marcus A. Cheek, Jonathan M. Burg, Zu-Wen Sun, Catherine A. Musselman, Michael-Christopher Keogh, Neil L. Kelleher*

Dear Editor,

Thank you for considering our manuscript, "Direct Readout of Multivalent Chromatin Reader-Nucleosome Interactions by Nucleosome Mass Spectrometry" at *ACS Central Science*. We greatly appreciate the considered and constructive reviews and opportunity to provide our point-by-point responses (blue text), with the manuscript modified accordingly.

Reviewer #1 (1.#)

1.1) make clearer the distinctions between this study and the previous 2021 Nat Meth work and the importance of knowing the modifications/combinatorial PTM information; state these clearly early in the manuscript and repeat them in the conclusions"

We thank the reviewer for highlighting this key point. Our 2021 study described the general method of **NucMS.** The current manuscript further optimizes the approach and extends to affinity capture with **Chromatin associated proteins (CAPs)** to determine the histone proteoforms that drive endogenous interactions. Of particular note, nucleosome engagement is invariably multivalent, so this is directly explored with native and synthetic 'tandem readers' mixed with fully-defined semi-synthetic or native nucleosomes. Establishing this proof of principle with multiple binding entities will

encourage the broader application of Nuc-MS to explore CAP : chromatin engagement in the normal and disease state.

1.2) use fewer acronyms in the abstract and define those that are used; the authors are so used to this field that they may not notice how difficult the acronyms are for non-experts but to publish in a broader journal, this needs to be corrected."

Apologies for the extensive use of domain names through the manuscript (including the abstract) but this is to avoid still-further confusion. Chromatin effector proteins are often littered with (presumed) functional domains. As an example, BPTF PHD-BD communicates these domains at the protein C-terminus (28673031) *vs.* the full-length protein (UniProt Q12830; 3,046aa / 338 kDa) that contains an additional PHD domain (390-437). We have addressed accessibility throughout the manuscript to enable a wider audience while still conveying the precision of our findings. Nomenclature in the chromatin field is highly challenging, with limited consensus on how to describe histone proteoforms, nucleoforms and experimental nuance. Indeed, the senior authors on this study are part of a larger community drive to adopt a nomenclature that enables more effective communication on these matters (revised manuscript attached):

> **Keogh M-C**, Almouzni G, Andrews AJ, Armache K-J, Arrowsmith CH, Baek SH, Bedford MT, Bernstein E, Côté J, David Y, Denu JM, Fierz B, Garcia BA, Glass KG, Gozani O, Helin K, Henikoff S, Jensen ON, **Kelleher NL**, Kutateladze TG, Lindner H, Lu C, Josefowicz SZ, Luger K,
> Mallack P, Musselman CA, Muir TW, Paša-Tolić L, Schneider R, Shi X, Shi Y, Sidoli S, Smith LM, Tyler JK, Wolberger C, Workman JL, Strahl BD & Young NL (in revision) A Needed Nomenclature for Nucleosomes. [MOLECULAR-CELL-D-25-00709]

1.3) explain/define the percentages mentioned e.g., when stating 75% enrichment

Addressed.

1.4) consider whether the charge states of the products have meaning (maybe only related to extent of unfolding)"

The reported charge state of each histone proteoform refers to the specific form used for characterization by tandem MS/MS and relative quantification based on intact mass. This is addressed in the revised figure legends and emphasized in the **Methods** section.

Reviewer #2 (2.#)

2.1) The Nuc-MS approach is a major technical advance, but the manuscript could briefly address practical considerations such as capture efficiency, sensitivity, and current limitations (e.g., need for high-end instrumentation, potential detection bias against low-abundance proteoforms)."

We thank the reviewer for acknowledging the technological advance of CAP-based affinity capture within Nuc-MS. Re. the practical questions for perspective re. current capabilities of the method, any limitations, and opportunities for improvement: these are now addressed through the **results and discussion** (see also **response 3.11**). In brief the hardware is a high-end Orbitrap-based mass spectrometer that would be classed as widely accessible (cost ~ $1M with ~1,500 so-called "tribrid Orbitraps" available in North America and Europe). As would be expected, CAP-mediated nucleosome enrichment would need to cross an efficiency threshold if material is to be available for analysis. We deliberately focus on tandem readers since combinatorial engagement is almost certainly the most biologically meaningful (and will invariably be of greatest affinity) [see also **response 2.2**]. We also note the ever-growing data on nucleosome binders from biochemical and genomics approaches [*e.g.*, PMIDs 34819353, 38319148; 10.1101/2025.04.29.651129], with such insights immediately transferable to Nuc-MS. The limitations of the method would be conveyed as detection limits of proteoform copy number and number of cells required for this enrichment-based approach. As there are no absolute reference for proteoform and PTM copy number, we are left with describing these limitations based on estimates.

2.2) For BPTF, the necessity of both PHD and bromodomain is clearly shown. However, for other readers (BRD4, DNMT3A–MPP8, SHL), it is assumed that both domains are required. If available, inclusion of single-domain controls (or discussion thereof) would strengthen the claim that multivalency drives specificity."

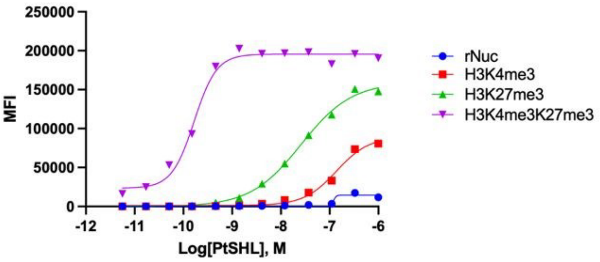

In the case of BPTF, the individual contribution of each reader domain (PHD and BD) and their synergy in the tandem context is in **Figure 2** and has previously been explored by biochemical and genomic approaches (*e.g.*, PMID 38319148). Similar observations of synergy have been made for DNMT3-MPP8 PWWP-CD [*e.g.*, PMID 28946896], BRD4 BD1-BD2 [*e.g.*, PMID 39938804] and PtSHL BAH-BD [where the tandem reader shows >100-fold enhanced binding to ($[H3K4me3K27me3]_2$) nucleosomes vs. those containing each PTM alone (the Figure shown here is from a manuscript in preparation; the approach is **Captify Luminex** as **Figure S1**].

2.3) The top-down MS/MS spectra are impressive and well-supported. However, we know that top-down struggles in mapping with high confidence modifications when isobaric forms are present (and histones have a lot!). Is it possible to understand how much of a risk is the presence of endogenous HeLa histones? It is a bit confusing to me how much in the sample is the CAP:Nuc and how much is the endogenous. The text claims that they are mixed. Maybe I did not understand."

Providing a full description of every isobaric proteoform in a mix of endogenous HeLa histones is indeed challenging. However, we have really upgraded the top-down MS/MS data quality in this revised submission, to the point of being able to clearly distinguish enriched histone H3 forms with 3-6 methyl equivalents, then isolate those with either three or six forms within a single proteoform window (*e.g.*, **Figures S15** and **S25**). The presented protocol uses bead-immobilized CAPs to

selectively capture their preferred nucleoforms, and several wash steps to isolate from unbound material and remove nonspecific interactions. This reduces sample complexity and permits a focus on distinguishing isobaric forms by top-down, tandem MS.

2.4) The work is analytically focused, but some contextualization of the proteoform combinations (e.g., H3K9me3 + K36me3 for DNMT3A–MPP8, or H3K4me3 + K27me3 + K79me2 for SHL) would
strengthen the biological relevance. Prior reports of such bivalent marks or chromatin states could be cited to guide interpretation."

The biological relevance of these PTM combinations is communicated in the revised text. Of note, both form counter-intuitive 'bivalent' signatures: *e.g.*,

> *'The DNMT3A-MPP8 reader fuses the H3K36me2/3 binding PWWP domain from de novo DNA methyltransferase 3A to the H3K9me3 binding chromodomain of M-phase Phosphoprotein 8 (MPP8)* ***(Figure S14)****.*
> *This chimera has been shown to be an effective tool for genomic and biochemical studies[95,96], and its combinatorial PTM targets a bivalent state ('repressive' H3K9me3 co-incident with 'transcriptionally active' H3K36me3) marking poised*
> *transcriptional enhancers.[97]'*

2.5) The observation of a 2:1 BPTF:nucleosome ratio is intriguing. Could this be due to GST dimerization or independent binding on each H3 tail? If untagged or cleaved protein was tested, please clarify. Otherwise, acknowledging this uncertainty would be sufficient."

We believe dimerization is the more likely possibility [PMID 24038604]. However, we have also shown that while GST-dimerization increases the avidity of BPTF PHD-BD for PTM-defined nucleosomes, this does not change the specificity of the interaction (when comparing GST-PHD-BD and 6HIS-PHD-BD queries: PMID 38319148).

2.6) Please, make the raw data available in MassIVE. I could not check the files."

Apologies for this error. The raw data is now publicly available in MassIVE: MSV000097336.

Reviewer #3 (3.#)

3.1) It is not made clear the difference between different bracketing schemes. It also at times inconsistent with the attached supplemental paper."

We note in the revised text that a new nucleoform nomenclature is being used (and confirmed correctly: our apologies for any inconsistencies). In this regard, the nomenclature proposal has been

resubmitted after review and further community discussions and is attached for reference (see also **response 1.2**).

3.2) "…requires coincident H3K4me3K9acK14acK18ac for effective nucleosome engagement." This statement seems a little too specific. Perhaps adding a qualifier "of the configurations tested?" I believe this is one of the proteoforms but there are undoubtedly others that are similarly preferred or even more."

This refers to BPTF PHD-BD engagement with semi-synthetic nucleosomes and is indeed of those tested in the Nuc-MS experiment (it could be further narrowed down to H3K4me3K14ac or H3K4me3K18ac since K9ac plays no direct role: PMID 38319148). This is clarified in the revised abstract.

3.3) It should be more clearly stated that K44ac probably would not have been considered for investigation using other approaches. Also, show the spectra in supplemental that localizes this PTM. There are ways to be wrong about this. Others have noted various adducts (sulfate adducts that survive ETD reactions) and oxidations in this region."

We have included the improved tandem MS/MS of this H4 proteoform in **Figure S9**. Our initial data was generated using tandem MS fragmentation by higher-collisional dissociation (HCD) across a distribution of H4 proteoforms. In the new **Figure S9**, we now provide spectra localizing the modification sites at K44ac and K20me2 by electron transfer dissociation (ETD) and isolated spectra of the corresponding intact H4 proteoform with no indications of adducts. We also note in passing that H4K44ac is not generally detected in proteoform analysis of bulk chromatin [*e.g.*, PMID 33589837].

3.4) Define "methyl equivalents" on first use. Also, this should be brought into the discussion about the limitations of the methods. Sometimes the authors are not truly discriminating proteoforms but more proteoform families based on shared precursor mass, which isn't particularly biologically meaningful."

This is now defined at first appearance ("*Nuc-MS of CAP:nuc complexes assembled from DNMT3A-MPP8 and endogenous HeLa nucleosomes (**Figure S4**) identified a proteoform landscape ~1.5-fold enriched for histone H3.2 containing three or more methyl equivalents (i.e., multiple additions of CH_3 (+14) to the primary sequence mass) **(Figure 4A)***"). As in the revised **Discussion**, histone proteoforms can often share similar precursor masses, and so care should be taken in data interpretation. To this end, we provide updated tandem MS/MS in the new **Figures S8**, **S9**, **S12**, **S15**, **S17**, **S19**, **S22**, **S24** and **S25** further supporting our assignment of designated histone PTMs within studied proteoforms.

3.5) H4K16acK20me2 is quite common. Please consider/discuss this when addressing H4K16acK20me2."

This is explicitly mentioned in the revised manuscript: '*Characterization of the other histone proteoforms revealed enrichment of {H4K16acK20me2}, a highly abundant H4 proteoform in bulk chromatin with both PTMs associated with active transcription **(Figures 4B** and **S16-17)**[53,56,57], …*'.

3.6) In SI Fig 7 why are so many N-terminal fragments missing? These should be the most prominent. While there are sufficient y ions for PTM localization it would stronger with both b- and y- ions to confirm proteoform ID. In SI fig 13 these near the N-terminus b- ions are present."

Consistent with **response 3.4**, we have provided updated graphical fragment maps of isolated BRD4 BD1BD2 enriched H4 proteoforms containing both c- and z- type ions localizing PTM sites in **Figure S7**. Tandem MS/MS spectra of selected H4 proteoforms provide additional support for these modifications (**Figure S8**).

3.7) SI Fig 16: The majority of the strongest peaks are not assigned. Without any chromatographic separation of the endogenous proteoform I would expect these spectra would be near uninterpretable since many possible PTM localizations for each precursor mass (structural isomers). Of course, it could be that the CAP binding is highly selective and only one (or a few) proteoforms may be present. This figure and the absence of other figures does not show well that the mixture of proteoforms is simplified."

As noted, the Nuc-MS used in this approach does not employ chromatographic separation: rather we use
CAP-mediated affinity purification of nucleoforms to reduce sample complexity. In the original **Figure S16** (now **Figure S25**) the resulting enriched histone proteoforms are distinct to each CAP. Further, there are indeed more histone proteoforms than directly discussed in the text. In this example, PtSHL BAH-BD yields {H3.2K4me3}, {H3.2K27me3}, and most interestingly {**H3.2K4me3K27me3**} in *cis* (*i.e.* on the same histone molecule: see also **response 2.2**). This will be of <u>extreme</u> interest to readers since the canonical
'bivalent signature' and all discussions of its biology only accommodates H3K4me3 and H3K27me3 in *trans* (*i.e.* in the same nucleosome but opposite H3 tails). This proteoform is further confirmed by additional tandem MS/MS in **Figure S25**.

3.8) I would have liked to see a few more examples of annotated MS3 spectra showing localization of PTMs to define proteoforms. I expect that there would be some contradictory evidence and perhaps some mixture of proteoforms present in the spectra. Certainly this would not imply misinterpretation but I would like to see for myself in supplemental information typical spectra and how mixed these spectra are. It should also be discussed in text, again claiming that the spectra is primarily one proteoform is completely valid even when revealing that it is not pure."

We provide upgraded tandem MS/MS in **Figure S8**, **S9**, **S12**, **S15**, **S17**, **S19**, **S22, S24** and **S25** localizing PTM sites to further support our assignments of designated histone proteoforms. We also emphasize new and improved data in **Figures S7**, **S8**, **S9**, **S15** and **S25**.

3.9) The number of H2B Sequences seems unusually limited compared to prior work from the Mag alb and others that addressed that the precursor masses are often isobaric and thus generate chimeric spectra. Is this because you are not distinguishing some variants and they are being grouped with others (but not delayed as such) or are you limited in dynamic range? Could you provide tandem MS that makes it clear that you are successfully discriminating between the known sequences or otherwise clearly state any ambiguity?"

This comment highlights the challenge in proper assignment of H2B proteoforms due to their high sequence similarity. We provide tandem MS/MS spectra of several H2B and H2A proteoforms which localize the key amino acid differences (**Figures S12**, **S18** and **S23**). While it is possible we under-estimate the possible H2B and H2A proteoforms in our samples, we only formally assigned if sufficient tandem MS/MS was available. In our minds, this issue is less of a limitation than the isobaric challenge histone H3 proteoforms present; these require the highest quality tandem MS information and we make this point in the revised manuscript.

3.10) This statement need much more support and perspective: "Of note the latter enriches combinatorial {H3K4me3K27me3} on the same histone tail in HeLa chromatin, and thus expands the potential biology of this widely studied bivalent signature." While this may be considered a small point about yet another combination of piston PTMs, its very existence in any human or mammal cells has been an issue of debate for more than decade. You would do the world a service by presenting the definitive evidence in a clear and easily understood form. You also need to put this in perspective. I do not think this result invalidates the general truth that K4me3-K27me3 ChIP-seq tracks are almost exclusively in trans. What is the relative abundance of this combination? What other marks are they with? It has been shown over and over that K4me3 prefers hyper acetylation. Is this not true for K4me3-K27me3. A paper could and possibly should be written on this one result. I would consider putting the spectral evidence for this form in a main figure."

This comment (see also **responses 2.2** and **3.7**) highlights how previous studies only describe the bivalent H3K4me3 and H3K27me3 signature in chromatin in *trans* (with supporting data a mix of sequential IP, PTMMS and enzymology where PRC activity is inhibited on H3K4me3 substrates). Remember here we are looking at the pool enriched by PtSHL BAH-BD, which strongly prefers the dual modification (**response 2.2 figure**). To explore further we isolated precursors corresponding to either H3.2 3x methyl equivalents (*i.e.* H3.2K4me3 or H3K27me3) or 6x methyl equivalents (*i.e.* H3K4me3K27me3) followed by tandem MS/MS (revised **Figure S25**, which includes updated graphical fragment maps and tandem MS/MS spectra for each proteoform). To directly address the relative abundances for each proposed proteoform in the PtSHL enriched fraction, we calculated relative abundances based on commonly positioned fragment ions (*i.e.* C41). This allows us to distinguish proteoforms and calculate the ratio of {H3.2K4me3} or {H3.2K27me3} : {H3K4me3K27me3} as ~3:1. We conducted similar isolation and tandem MS/MS experiments to characterize the H3 proteoforms enriched by DNMT-MPP8 in revised **Figure S15**.

3.11) Overall, while this work is important, well written, and methodologically solid, the manuscript in its current form is unsuitable for publication. It is lacking primarily in the clear presentation of evidence that support the author's claims without requiring substantial work of the reader who will often not have the tools or the knowledge to retrieve the data and do their own analysis. It is also lacking in realistic perspective that clearly represents the limitations of this exciting new methodology and the proper implications of their findings such that others will not over simplify or over estimate. Think of the ways that a less informed reader might over reach in their assumptions. "K4me3-K27me3 is in cis not trans." "There are only a few H2B sequences in HeLA cells." These readers only enrich one specific proteoform and has little affinity for others. The method produces clean unmixed tandem mass spectra."

In the revised manuscript we clarify statements that might mislead the wider audience in their interpretation of our findings. We also provide perspective regarding potential limitations of Nuc-MS technology (as in **response 2.1**).

Thank you collectively for such deep consideration of this work. We look forward to hearing of the responses to our substantially revised and better substantiated manuscript.

With best regards,

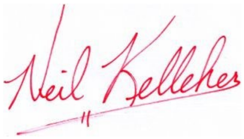

Neil Kelleher
Departments of Chemistry and Molecular Biosciences, Northwestern University
2145 Sheridan Rd, Evanston, IL
60208 Ph: 847-467-4362 n-kelleher@northwestern.edu

oc-2025-00736j.R2

Name: Peer Review Information for "Direct Readout of Multivalent Chromatin Reader-Nucleosome Interactions by Nucleosome Mass Spectrometry"

First/Second/Third Round of Reviewer Comments

Reviewer: 3

Comments to the Author

The authors have responded sincerely to the previous comments. Particularly, the inclusion of evidence supporting their claims is helpful. However, this greater transparency has exposed some significant concerns. I hope that the authors appreciate that careful attention to rigor is a benefit the authors. It would be detrimental to publish poorly supported or mistaken conclusions.

Thank you for providing more detailed evidence supporting your claims, notably supporting {H3K4me3K27me3} identification. My first instinct was to accept that there was sufficient

rigor applied since the evidence in now present. Unfortunately, I have new concerns and need to ask additional questions.

1) The evidence supporting {H3K4me3K27me3} appears to be specific to H3.2 and includes an N-terminal acetylation. It is my understanding from reading your methods section carefully that these native nucleosomes are derived from HeLa cells, which are well studied. To my knowledge this is also the first report of N-terminal acetylation of H3.2. A very thorough review by the Kirmisiz lab, who are experts in NAT biology from 2023 states "H3 is predicted to be Nt-acetylated based on its sequence, although this has not been experimentally demonstrated yet". (J Cell Sci (2023) 136 (7): jcs260801.) There are additional recent review articles that state that H3 is not N-terminally acetylated. The summary of the literature consensus is that H4 and all H1 sequences are nearly completely always N-terminally acetylated, most H2As are N-terminally acetylated with the exception of H2AZ, but no H2B sequences nor H3 sequences are N-terminally acetylated. There are many research papers, including from the Kelleher lab that show that H3 is not N-terminally acetylated. While it's possible that there are prior reports of H3.2 acetylation, it is well established that N-terminal acetylation of H3.2 is far from common and specifically from the species and cell line used. I do not understand how N-α-ac would be enriched from essentially non-existent to detectable using your approach. It should be said that events co-occuring at a rate much higher or lower than expected by random chance is indeed the central concept as to why native/top down proteomics has such immense promise. However, these events seem vanishingly unlikely. Even if this is true it is absolutely remarkable and should be discussed in depth. I suspect that you may have inadvertently fixed N-ac, thereby constraining the problem to biologically unlikely solutions; however, I could not find your description of search parameters or constraints on PTM localisation. Without this constraint of fixed N-ac the c-ion series will likely be recovered.

2) Your spectra in S25 supporting this assignment appear to be entirely lacking the early c-ion series that are almost universally present in other analyses. These ions are usually very abundant and provide the strongest evidence for PTM localization near the N-terminus. For example you localize acetyl to the N-terminus but you actually can only state with positive evidence (ions with the acetyl present in the ion) that it is within the first 36 or 41 residues. There are many known N-ε-ac sites included in the first 36 or 41 residues. Furthermore there are several alternate me3 sites. It is also notable that the c-ion series is only

recovered after K36 where additional methyl groups may be possibly located. The z-ion series only localizes by absence.

3) The deep z-ions used to support this assignment are weak and prone to misidentification, especially when deconvoluted.

4) Might it be possible to distinguish ac from me3 by mass? Such an analysis may bring clarity? There is insufficient digits for me to make this analysis myself.

5) It has been established previously that hyper acetylation is commonly co-occuring and perhaps necessary for H3K4me3. Why is there such limited acetylation in this case? I can accept that perhaps the bivalent state is hypoacetylated (but see below). If true this unusual conclusion should be discussed in depth.

6) The infusion and thus co-fragmentation without chromatography makes it more prone to misinterpretation. The assumption that there are only one or two proteoforms present seems unfounded and unlikely. It is established that correct proteoform identification can become impossible if there is insufficient separation of proteoforms either chromatographically or by mass. E.g. Phanstiel et al. (Proc Natl Acad Sci U S A. 2008 Mar 18;105(11):4093-8) clearly describe how some methyl equivalent states are not mathematically solvable without chromatographic separation. Many of the ions used for identification are shared with other possible proteoforms. There are few fragment ions that are unique and can not be explained by the presence of a mixture of other proteoforms. It would help to show the absence of any ions supporting presence of methyl groups at K9 & K36 without +/-14, +/-28 +/-42 satellite peaks at relevant ions. Often each ion has satellite peaks +/- 14, representing alternate localizations. How good is the match to alternative proteoform designations? What about mixtures?

7) The raw, non-deconvolved spectra is the better evidence for supporting such important claims.

Your quantitative estimate of cis- vs. trans- K4me3 K27me3 appears flawed. Most K4me3 is found in hyperacetylated states, at least when not in cis with K27me3. Failure to include most of the K4me3 in your estimate makes it erroneous. I.e. an actual measurement of this would at least need to include estimates from unenriched samples across the observed precursors masses not and arbitrarily selected limited set of precursors from an enriched sample. This is due both to precursor mass selection excluding these higher modified states but also due to your enrichment. What this *might* represent is how much your enrichment strategies are picking up K4me3 alone. At the very least this needs clarification.

If your identification of {H3K4me3K27me3} is indeed correct it would be helpful to the chromatin field to accurately comment on the relative abundance of {H3K4me3K27me3}. I expect that some readers may interpret these results as indicating that K4me3 and K27me3 exist primarily in cis. Your claimed {H3K4me3K27me3} comes out of very strong enrichment. While these results are not quantitative it notable that ({H3K4me3}•{H3K27me3}) and the constituent proteins are readily observed without enrichment for over a decade. Your results are the first truly convincing evidence of {H3K4me3K27me3} and it required very unique enrichment approaches. I would at the very least suggest language in the discussion section such as “Identification of {H3K4me3K27me3}, in cis, required a unique and highly specific enrichment strategy. This suggests that it is far less common than the trans configuration, ({H3K4me3}•{H3K27me3}), evidence of which is readily detected without enrichment.”

There is also some lack of clarity as to what is meant by 3-6 methyl equivalents. Does N-ac count as three methyl equivalents?

In the figure 3 caption I think you mean {H2A.ZK15ac} not {H2A.K20me2K44ac}.

The consistency of the new nomenclature continues to confuse me a bit. It seems a little over simplified in this paper. It is presented as native vs semi-synthetic; however, the attached paper indicates that it is partially characterized (Some PTMs localized) vs fully characterized (All PTMs localized).

I note that I have primarily focused on one specific claim of great significance. Similar rigor should be applied elsewhere.

Reviewer: 2

Comments to the Author

The manuscript of Lee et al. presents an innovative application of Nucleosome Mass Spectrometry (Nuc-MS) to directly characterize interactions between chromatin-associated proteins (CAPs) and intact nucleosomes. By combining semi-synthetic nucleosomes with defined modifications and endogenous HeLa-derived nucleosomes, the authors convincingly demonstrate the ability of Nuc-MS to (i) detect multivalent reader–nucleosome interactions and (ii) resolve the specific histone proteoforms that drive binding. Interestingly, the authors are already adopting the new nucleosome nomenclature that was recently accepted in Molecular Cell, for which many mass spectrometrists working on top-down and chromatin biology collaborated to forge.

The manuscript is well written, methodologically rigorous, and broadly relevant to the chromatin and proteomics communities. I just have a few very minor comments.

1) The cis {H3K4me3K27me3} finding is intriguing, as this “bivalent” state has been traditionally described in trans. Can the authors comment on this aspect, maybe briefly discussing the implication with what has been known regarding these bivalent domains in stem cells?

2) While Nuc-MS enrichment profiles are clear, some comparisons to established biochemical approaches (peptide pull-downs, ChIP) would help contextualize sensitivity and specificity. Maybe there is no need for additional experiments, but at least the authors can comment on limitations of Nuc-MS compared to widely used pull-downs.

3) For BPTF, two molecules were detected per nucleosome, but the mode of binding was uncertain. Further discussion (or experiments such as crosslinking MS or structural modeling) would clarify whether this reflects genuine cooperativity or artifact from the GST-tag.

4) The methods are described clearly, but because Nuc-MS is a specialized technique, it would be valuable to provide guidance on sample requirements, sensitivity, and potential limitations (e.g., nucleosome yield, MS instrument availability).

Reviewer: 1

Comments to the Author

The authors made several important changes to the manuscript. The work is of high quality and significant, so I'd like to see it published in ACS Central Science. Unfortunately, I still believe that the authors missed the mark on the comments of reviewer 1, points 1.1 and 1.2, and the more clearly stated request of reviewer 3, point 3.11. In its current form, the manuscript is only accessible to those who have background knowledge in nucleosomes. If the authors want general readers of ACS Central Science to read the manuscript, the language in the abstract, text, and conclusions needs to be broadened. Proteoform and nucleoform need to be defined early on in the manuscript and the field in general needs to be described in layperson's language.

Although the following may not be accurate, this type of language could go a long way to help the general reader.

Scientists are trying to understand how certain proteins (called chromatin-associated proteins, or CAPs) interact with DNA to control which genes are turned on or off. These proteins often recognize chemical modifications on histone proteins — the proteins that DNA wraps around. Proteins with different post-translational modification (PTM) patterns are called proteoforms.

Most studies look at the modifications using pieces of protein histones, but that doesn’t show the full picture. In real life, histones exist inside nucleosomes — full structures made of DNA and histones — and they often have multiple modifications at once, with the overall different nucleosome compositions referred to as nucleoforms. The complexity of proteoforms and nucleoforms matters because small changes such as x, z, cause z. .

Author's Response to Peer Review Comments:

Walter and Mary Elizabeth Glass Professor in the Life Sciences
Director, Proteomics Center of Excellence
Director, Chemistry of Life Processes Institute
Professor in the Departments of Chemistry and Molecular Biosciences and the Feinberg School of Medicine

Office Address
3613 Silverman Hall
2170 Campus Drive
Evanston, IL 60208

Phone: 847-467-4362
Fax: 847-467-3276

Mailing Address
2-100 Hogan Hall
2205 Tech Drive
Evanston, IL 60208

Email: n-kelleher@northwestern.edu
Research group website: www.kelleher.northwestern.edu
Center website: http://pce.northwestern.edu

January 7th, 2026

Executive Editor
American Chemical Science Central Science

Manuscript: Direct Readout of Multivalent Chromatin Reader-Nucleosome Interactions by Nucleosome Mass Spectrometry

Authors: Alexander S. Lee, Nickolas P. Fisher, Matthew R. Marunde, Pei Su, Laiba F. Khan, **Ryan J. Ezell**, **Zachary B. Gillespie**, Bria Graham, Hailey F. Taylor, Ugochi C. Onuoha, Taojunfeng Su, Kevin Jooß, Luis F. Schachner, Harrison A. Fuchs, Kelsey Noll, **Matthew J. Meiners**, Marcus A. Cheek, Jonathan M. Burg, Zu-Wen Sun, Catherine A. Musselman, Michael-Christopher Keogh, Neil L. Kelleher*

Dear Editor,

Thank you for considering our manuscript, "Direct Readout of Multivalent Chromatin Reader-Nucleosome Interactions by Nucleosome Mass Spectrometry" at *ACS Central Science*. We greatly appreciate the constructive reviews, supportive comments regarding the importance of our work, and opportunity to provide point-by-point responses. The manuscript text has been modified accordingly. Please note co-author additions (**red** above) to reflect contributions to this revision.

Reviewer #1 (1.#)

1.1) The authors made several important changes to the manuscript. The work is of high quality and significant, so I'd like to see it published in ACS Central Science. Unfortunately, I still believe that the authors missed the mark on the comments of reviewer 1, points 1.1 and 1.2, and the more clearly stated request of reviewer 3, point 3.11. In its current form, the manuscript is only accessible

to those who have background knowledge in nucleosomes. If the authors want general readers of ACS Central Science to read the manuscript, the language in the abstract, text, and conclusions needs to be broadened. Proteoform and nucleoform need to be defined early on in the manuscript and the field in general needs to be described in layperson's language.

We truly appreciate the positive comments and suggestions. Balancing general accessibility at the cutting edge of molecular precision has become truly challenging in the chromatin field, and indeed two of the senior authors on this study recently published a nomenclature in *Molecular Cell* to communicate specificity and nuance at the nucleosome-level [PMID 41043390] (we gratefully note the Reviewers use of this).

To address clarity and flow in the revised manuscript we explicitly define histone proteoform and nucleoform early and other concepts throughout. Here we were guided by several recent papers in *ACS Central Science* centered around chromatin and nucleosomes [PMIDs 31041386, 34235262 and 35233450] and aimed our language accordingly.

Reviewer #2 (2.#)

2.1) The cis {H3K4me3K27me3} finding is intriguing, as this "bivalent" state has been traditionally described in trans. Can the authors comment on this aspect, maybe briefly discussing the implication with what has been known regarding these bivalent domains in stem cells?

In the chromatin context 'bivalency' refers to the spatial co-localization of two histone post-translational modifications (PTMs) that are usually in functional opposition: the canonical being **H3K4me3** (associated with active promoters) and **H3K27me3** (associated with polycomb repressed regions). Containing this within the same nucleosome requires the responsible enzymes to write onto substrates they would rather avoid (due to impeded recruitment and/or enzymology), and further what could the PTMs achieve when co-incident? The traditional description that they are distributed across the two H3 molecules in a single nucleosome (in *trans*) provides biological possibilities for an ESC: at cell division one daughter inherits the positive configuration / one the negative and an epigenetic commitment has been made [PMID 16630819]. **Here we demonstrate an additional complexity**. In newly added data we show that when presented with a range of fully defined nucleosomes the plant PtSHL PHD-BAH tandem-reader preferentially engages ([H3]•[H3K4me3K27me3]) >> ([H3K4me3]•[K27me3]) (*i.e.*, *cis* >> *trans*) (**Figure 5D-E**); and further, enriches from native HeLa nucleosomes an MS signature compatible with {H3K4me3K27me3} (**Figures 5A & S26-S27**]. This is a novel configuration / observation and future studies will determine if it is restricted to HeLa (a cell line first cultured in 1951) or represents a new developmental opportunity for primary cells, including ESCs.

2.2) While Nuc-MS enrichment profiles are clear, some comparisons to established biochemical approaches (peptide pull-downs, ChIP) would help contextualize sensitivity and specificity. Maybe

there is no need for additional experiments, but at least the authors can comment on limitations of Nuc-MS compared to widely used pull-downs.

In the **Introduction**, we compare and contrast Nuc-MS with alternate approaches. In brief (and as in PMID 41043390), immunoblots, ChIP-seq or CUT&RUN use antibodies that detect a single PTM and rarely inform on any of its relationships in *cis* (*i.e.* the proteoform). Bottom-up proteomics can describe proximal PTMs on the same peptide but generally loses any connections to distal marks. At the next level, the configuration of histone PTMs and variants within the same nucleosome (*i.e.* the nucleoform) are presumed functionally important. Histone peptide pulldowns cannot fully represent the multiple binding surfaces this entity offers (*e.g.*, nucleosomal DNA, acidic patch, primary sequences or PTMs on the same or adjacent histones) for CAP interactions. Current methods to interrogate bulk samples are either unable to distinguish (*e.g.*, immunoblot, ChIP-seq, CUT&RUN), can discriminate some possibilities (*e.g.*, sequential ChIP and middle-down MS), or have the potential to be definitive (*e.g.*, combinatorial enrichment and **Nuc-MS**). For context we now indicate the sample input requirement for each approach.

To address the specificity point: the main limitation of Nuc-MS lies in alternate interpretations of the proteoform landscape presented (as with all experiments it is often difficult to be definitive, and thus best used with supporting approaches). This is clearly stated in the discussion.

2.3) For BPTF, two molecules were detected per nucleosome, but the mode of binding was uncertain. Further discussion (or experiments such as crosslinking MS or structural modeling) would clarify whether this reflects genuine cooperativity or artifact from the GST-tag.

We have extensively characterized the influence of GST dimerization [PMID 24038604] on reader domain binding to PTM-defined nucleosomes. In a recent study we observed m/z peaks corresponding to dimerized GST-RAG2 PHD bound to ($[H3K4me3]_2$) nucleosomes [PMID 41182902]. GST.BPTF PHD-BD (potentially dimeric) has an increased binding affinity for PTM-defined nucleosomes over 6xHis.PHD-BD (invariably monomeric), though each displays the same target preference [PMID 38319148]. As such GST dimerization can provide an avidity boost but does not drive binding specificity for multiple reader : PTM combinations.

This issue distracts from the central demonstration that combinatorial engagement of PHD-BD with ($[H3K4me3K9acK14acK18ac]_2$) requires both reader domains and both PTM classes (**Figure 2** and PMID 38319148). **Figure 2** demonstrates the capability of Nuc-MS with fully defined reagents before embarking on studies with undefined native nucleosomes. Of note, crosslinking MS is a powerful way to identify proteinprotein interfaces but does not generally yield insight on stoichiometry (would only do with stable isotope labelled peptide standards and extensive assay development).

2.4) The methods are described clearly, but because Nuc-MS is a specialized technique, it would be valuable to provide guidance on sample requirements, sensitivity, and potential limitations (e.g., nucleosome yield, MS instrument availability).

In the revised submission we note that Nuc-MS can be implemented with widely available Orbitrap-based mass spectrometers, add context on its limitations (as in response **2.2**), and give additional technical details (see main text and Methods).

Reviewer #3 (3.#)

3.1) The evidence supporting {H3K4me3K27me3} appears to be specific to H3.2 and includes an N-terminal acetylation. It is my understanding from reading your methods section carefully that these native nucleosomes are derived from HeLa cells, which are well studied. To my knowledge this is also the first report of Nterminal acetylation of H3.2. A very thorough review by the Kirmisiz lab, who are experts in NAT biology from 2023 states "H3 is predicted to be Nt-acetylated based on its sequence, although this has not been experimentally demonstrated yet". (J Cell Sci (2023) 136 (7): jcs260801.) There are additional recent review articles that state that H3 is not N-terminally acetylated. The summary of the literature consensus is that H4 and all H1 sequences are nearly completely always N-terminally acetylated, most H2As are N-terminally acetylated with the exception of H2AZ, but no H2B sequences nor H3 sequences are N-terminally acetylated. There are many research papers, including from the Kelleher lab that show that H3 is not N-terminally acetylated. While it's possible that there are prior reports of H3.2 acetylation, it is well established that Nterminal acetylation of H3.2 is far from common and specifically from the species and cell line used. I do not understand how N-α-ac would be enriched from essentially non-existent to detectable using your approach. It should be said that events co-occuring at a rate much higher or lower than expected by random chance is indeed the central concept as to why native/top down proteomics has such immense promise. However, these events seem vanishingly unlikely. Even if this is true it is absolutely remarkable and should be discussed in depth. I suspect that you may have inadvertently fixed N-ac, thereby constraining the problem to biologically unlikely solutions; however, I could not find your description of search parameters or constraints on PTM localisation. Without this constraint of fixed N-ac the c-ion series will likely be recovered.

We thank the reviewer for catching this error and agree that histone H3 is **not** commonly N-terminally acetylated [as in PMIDs 38960040, 26272979 and 33818074]. Our revised H3.2 analyses does not contain this modification.

3.2) Your spectra in S25 supporting this assignment appear to be entirely lacking the early c-ion series that are almost universally present in other analyses. These ions are usually very abundant and provide the strongest evidence for PTM localization near the N-terminus. For example, you localize acetyl to the N-terminus but you actually can only state with positive evidence (ions with the acetyl present in the ion) that it is within the first 36 or 41 residues. There are many known N-ε-ac sites included in the first 36 or 41 residues.
Furthermore there are several alternate me3 sites. It is also notable that the c-ion series is only recovered after K36 where additional methyl groups may be possibly located. The z-ion series only localizes by absence. Corrected (see also response **3.1**).

3.3) The deep z-ions used to support this assignment are weak and prone to misidentification, especially when deconvoluted.

Corrected (see also response **3.6**).

3.4) Might it be possible to distinguish ac from me3 by mass? Such an analysis may bring clarity? There is insufficient digits for me to make this analysis myself.

Addressed (see also response to **3.10**)

3.5) It has been established previously that hyper acetylation is commonly co-occuring and perhaps necessary for H3K4me3. Why is there such limited acetylation in this case? I can accept that perhaps the bivalent state is hypoacetylated (but see below). If true this unusual conclusion should be discussed in depth.

The reviewer is correct in that H3K4me3 (in its 'standard' role as an activating mark) most often co-occurs with various hyperacetylated states [PMIDs 38960040, 37204295, and 26474904] and indeed the mechanism of this has been identified: histone tail acetylation directly disengages the H3 tail from the nucleosome surface to enable MLL1 writer activity [PMIDs 37204295]. Indeed we observe (through new **denatured LC-TopDown MS** (dLC-TD-MS) data) fragment ions that support the presence of H3K4me2/3 co-occurring with acetylation K14 and K18 [**Figure S26-27**] (see also response to **3.6**).

It should be noted it has not been thoroughly described if a 'classical' bivalent nucleosome ({H3K4me3}•{K27me3}) follows the *cis*-acetylation-enables-H3K4me3 pathway (an important consideration since ({H3}•{K4me3}) is a poor substrate for PRC to write H3K27me3) (see also response to **2.1**).

3.6) The infusion and thus co-fragmentation without chromatography makes it more prone to misinterpretation. The assumption that there are only one or two proteoforms present seems unfounded and unlikely. It is established that correct proteoform identification can become impossible if there is insufficient separation of proteoforms either chromatographically or by mass. E.g. Phanstiel et al. (Proc Natl Acad Sci U S A. 2008 Mar 18;105(11):4093-8) clearly describe how some methyl equivalent states are not mathematically solvable without chromatographic separation. Many of the ions used for identification are shared with other possible proteoforms. There are few fragment ions that are unique and can not be explained by the presence of a mixture of other proteoforms. It would help to show the absence of any ions supporting presence of methyl groups at K9 & K36 without +/-14, +/-28 +/-42 satellite peaks at relevant ions. Often each ion has satellite peaks +/- 14, representing alternate localizations. How good is the match to alternative proteoform designations? What about mixtures?

The reviewer is correct in the difficulty deconvoluting proteoforms in an unresolved complex mixture (*e.g.*, HeLa bulk nucleosomes), but this can be addressed by an initial separation step (*e.g.*, affinity, ion exchange or size-exclusion chromatography). Indeed, this is **precisely** what we do with

each tandem reader query: affinity enrichment prior to Nuc-MS of the captured material (now dramatically reduced in complexity).

To add confidence in our assignment of H3.2 proteoforms, we have conducted **dLC-TD-MS** of (3x, 6x, and 9x) methyl equivalents from DNMT3A-MPP8 and PtSHL-enriched endogenous nucleosomes. We further conducted a parallel reaction monitoring (PRM) workflow tailored for the analysis of these proteoforms [PMID 38354049]. By coupling LC-based separation and PRM for the targeted isolation (quadrupole isolation window of 0.8 *m/z*) and subsequent EThcD fragmentation of defined precursors ions, we can confidently assign each H3.2 proteoform from ever more complex mixtures. This is somewhat like the situation in bottom-up MS, where histone peptides carrying specific marks can be confidently reported, yet their absolute abundance in bulk chromatin is difficult to estimate.

3.7) The raw, non-deconvolved spectra is the better evidence for supporting such important claims.

In the revised submission (and as in response **3.6**), we provide key non-deconvoluted spectra of *b* and *c* ions from our dLC-TD-MS experiments, thus highlighting key modification sites of H3.2 proteoforms.

3.8) Your quantitative estimate of cis- vs. trans- K4me3 K27me3 appears flawed. Most K4me3 is found in hyperacetylated states, at least when not in cis with K27me3. Failure to include most of the K4me3 in your estimate makes it erroneous. I.e. an actual measurement of this would at least need to include estimates from unenriched samples across the observed precursors masses not and arbitrarily selected limited set of precursors from an enriched sample. This is due both to precursor mass selection excluding these higher modified states but also due to your enrichment. What this *might* represent is how much your enrichment strategies are picking up K4me3 alone. At the very least this needs clarification.

The revised submission includes dLC-TD-MS data of (3x, 6x, and 9x) methyl equivalents (see also response **3.6**). This search was limited to generate a histone H3.2 proteoform search list with highly matched manually validated fragment ions (*i.e.* all major N-terminal lysine residues from amino acids 1-40). For this search list we implemented Proteoform Finder [PMID 38354049], a software tool that provides relative quantification of proteoforms by analyzing fragment ions resulting from PRM-based experiments. Proteoform Finder uses matched fragment ions for each histone proteoform to output XIC and AUC values to quantify each histone proteoform, which represent their overall abundance. **In this manner**: while we cannot precisely determine the CAP-specific enrichment of a histone proteoform relative to bulk chromatin, we can assert the histone proteoforms enriched and their relative abundances.

3.9) If your identification of {H3K4me3K27me3} is indeed correct it would be helpful to the chromatin field to accurately comment on the relative abundance of {H3K4me3K27me3}. I expect that some readers may interpret these results as indicating that K4me3 and K27me3 exist primarily in cis. Your claimed
{H3K4me3K27me3} comes out of very strong enrichment. While these results are not quantitative it notable that ({H3K4me3}•{H3K27me3}) and the constituent proteins are readily observed

without enrichment for over a decade. Your results are the first truly convincing evidence of {H3K4me3K27me3} and it required very unique enrichment approaches. I would at the very least suggest language in the discussion section such as "Identification of {H3K4me3K27me3}, in cis, required a unique and highly specific enrichment strategy. This suggests that it is far less common than the trans configuration, ({H3K4me3}•{H3K27me3}), evidence of which is readily detected without enrichment."

The reviewer is entirely correct that identifying {H3K4me3K27me3} from Hela cells was a surprise, and yet
PtSHL most effectively binds this nucleoform when presented with fully PTM-defined nucleosomes (new
Figure 5D-E) and further, enriches a compatible MS signature from HeLa cell bulk nucleosomes (**Figures 5A** & **S26-27**]. As in **2.1**, this is a novel configuration / observation and future studies will determine if it is restricted to HeLa (a cell line first cultured in 1951) or also represents a new developmental opportunity for ESCs. How the field could have 'missed' this new PTM combinatorial for so long is addressed in the revised discussion.

3.10) There is also some lack of clarity as to what is meant by 3-6 methyl equivalents. Does N-ac count as three methyl equivalents?

A greater description of 'methylation equivalents' is provided in the revised text and further explained in cited PMIDs 27371874 and 26272979. The reviewer is correct that N-ac [42.0106 Da] is close in mass to three methyl equivalents [42.0471 Da] which can provide a challenge to assign (is 6 = [ac + me3] or [2 x me3] or [2 x ac]). However, it is possible to distinguish the two PTM classes which relies on quadrupole isolation with a stringent isolation window (0.6-0.8 m/z) [PMID 26272979].

3.11) In the figure 3 caption I think you mean {H2A.ZK15ac} not {H2A.K20me2K44ac}.

We thank the reviewer for catching this mistake and have corrected it.

3.12) The consistency of the new nomenclature continues to confuse me a bit. It seems a little over simplified in this paper. It is presented as native vs semi-synthetic; however, the attached paper indicates that it is partially characterized (Some PTMs localized) vs fully characterized (All PTMs localized).

The new nomenclature to communicate specificity and nuance at the nucleosome-level was recently published [PMID 41043390] and is used through this revision. As therein: '*Square brackets "[H3K4me3]"*
indicate a fully defined proteoform, as in a semi-synthetic histone, where other sites of potential modification not denoted can be understood as definitively unmodified. Braces "{H3K4me3}" indicate a partially understood native proteoform, where one PTM is experimentally known to exist (as by immunoprecipitating H3K4me3 from a cell extract), but other sites of potential modification not denoted are understood to be of undefined status: the general situation with native material'. Within this, the suggested approach is: **'if any uncertainty {} should be used**' : native material under study could have additional heterogeneity beyond experimental

resolution and so this uncertainty should be expressed. Communicating this effectively has caused some of the back-and-forth through this review and we are grateful to the reviewers for their diligence.

As an example, we use dLC-TD-MS (see response **3.6**) for the targeted analysis of H3.2 proteoforms with 3x, 6x, and 9x methyl equivalents from both DNMT3A-MPP8 and PtSHL-enriched endogenous nucleosomes. Detailed analysis of the 6x pool (by LC-MS/MS) suggested many H3.2 proteoforms containing methylation and acetylation equating to 5x methyl, suggesting the resolution of our targeted analysis and a modification site we were unable to fully localize and identify. A technology capable of the complete readout and quantitation of the >5,000 proteoforms in a sample of endogenous H3.2 does not yet exist. Here we show enrichment is clearly possible, and can reduce sample complexity to the stage where more insights to the proteoform landcape can truly inform potential biology. We make reference to this in the limitations section of our revised manuscript to help the generalist understand the current state of play in protein sequencing.

Thank you collectively for such deep consideration of this work. We look forward to hearing of the response to our substantially revised manuscript.

With best regards,

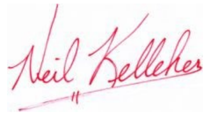

Neil Kelleher
Departments of Chemistry and Molecular Biosciences, Northwestern University
2145 Sheridan Rd, Evanston, IL
60208 Ph: 847-467-4362 n-
kelleher@northwestern.edu
